# Integrated Structural, Physicochemical, Pharmacokinetic and Pharmacodynamic Evaluation of Substituted Phenylcarbonyloxypiperazinopropanols as Antimycobacterial Agents

**DOI:** 10.3390/life16101593

**Published:** 2026-09-23

**Authors:** Dominika Nádaská, Tomáš Sádecký, Lucia Račková, Martina Hrčka Dubničková, Fils Andriamainty, Petr Mokrý, Simona Gover, Vladimír Garaj, Jozef Csöllei, Ivan Malík

**Affiliations:** 1 Department of Pharmaceutical Chemistry, Faculty of Pharmacy, Comenius University Bratislava, Odbojárov 10, P.O.Box 90, SK-820 18 Bratislava, Slovakia; sadecky8@uniba.sk (T.S.); andriamainty@fpharm.uniba.sk (F.A.); garaj1@uniba.sk (V.G.); 2 Institute of Chemistry, Slovak Academy of Sciences, Dúbravská Cesta 9, SK-845 38 Bratislava, Slovakia; lucia.rackova@savba.sk; 3 Department of Pharmacognosy and Botany, Faculty of Pharmacy, Comenius University Bratislava, Odbojárov 10, P.O.Box 90, SK-820 18 Bratislava, Slovakia; 4 Centre of Experimental Medicine, Institute of Experimental Pharmacology and Toxicology, Slovak Academy of Sciences, Dúbravská Cesta 9, SK-841 04 Bratislava, Slovakia; 5 Department of Cell and Molecular Biology of Drugs, Faculty of Pharmacy, Comenius University Bratislava, Odbojárov 10, P.O.Box 90, SK-820 18 Bratislava, Slovakia; martina.hrcka.dubnickova@uniba.sk; 6 Department of Chemical Drugs, Faculty of Pharmacy, Masaryk University in Brno, Palackého Třída 1946/1, CZ-612 00 Brno, Czech Republic; mokryp@pharm.muni.cz (P.M.); csolleij@pharm.muni.cz (J.C.)

**Keywords:** arylcarbonyloxypiperazinopropanols, *N*-arylpiperazines, antimycobacterial activity, ATP synthase

## Abstract

This research aimed to evaluate structural, physicochemical and pharmacokinetic characteristics of racemic 2-hydroxy-3-(4-phenylpiperazin-1-yl)propyl 4-[(alkoxycarbonyl)amino]benzoates (**Ib**)–(**IVb**) and 2-hydroxy-3-[4-(*R*-substituted phenyl)piperazin-1-yl]propyl 4-[(alkoxycarbonyl)amino]benzoates (**1b**)–(**12b**), while further extending the in vitro screening of their racemic salts, 1-(3-{4-[(alkoxycarbonyl)amino]benzoyloxy}-2-hydroxypropyl)-4-(*R*-substituted phenyl)piperazin-1-ium chlorides (**I**)–(**IV**) and (**1**)–(**12**) (alkoxy = methoxy to butoxy in all of these series, and *R* = H, 4′-F, 2′-F or 2′-OCH_3_), against several mycobacterial species, including a slow-growing virulent *Mycobacterium tuberculosis* (*Mtb*) H_37_R_v_ strain. The values of structural, physicochemical and several pharmacokinetic descriptors of the compounds (**Ib**)–(**IVb**) and (**1b**)–(**12b**) were generated in silico, employing a palette of interactive web-based engines and standalone software packages. The obtained data allowed for exploration of their mutual relationships through a chemometric principal component analysis method. The predicted outputs were compared to the values of respective descriptors defining antimycobacterial drugs approved worldwide for therapeutic use in human medicine. Molecular docking (MD) analyses were carried out to explore the possibilities of whether particular (*R*)- and (*S*)-stereoisomers of non-protonated derivatives, as well as (*R*)- and (*S*)-stereoisomeric forms of their salts (**I**)–(**IV**) and (**1**)–(**12**) would effectively interact with a proposed pharmacological target, adenosine 5′-triphosphate (ATP) synthase. This membrane–protein complex was considered essential for energy metabolism and survival of various mycobacterial species. Respecting the fundamental structural arrangement of more efficient salts, the importance of their structural and spatial features was highlighted to achieve relative effective interactions with particular subunits of ATP synthase present in *Mtb* H_37_R_v_. The results of the MD evaluations could contribute to the understanding of how these compounds inhibited growth of *Mtb* H_37_R_v_. 1-(3-{4-[(Butoxycarbonyl)amino]benzoyloxy}-2-hydroxypropyl)-4-phenylpiperazin-1-ium chloride (**IV**) with a minimum inhibitory concentration (*MIC*) value of 62.5 μM estimated in vitro and 1-(3-{4-[(butoxycarbonyl)amino]benzoyloxy}-2-hydroxypropyl)-4-(2-methoxyphenyl)piperazin-1-ium chloride (**12**) with *MIC* = 16 μM, as the most effective anti-*Mtb* H_37_R_v_ agents of each series of the salts, were screened in vitro to investigate their antiproliferative or (eventual) cytotoxic properties using a normal human dermal fibroblast cell line. The derivative (**IV**) decreased cell viability slightly more compared to the molecule (**12**).

## 1. Introduction

*N*-Arylpiperazines (NAPs) represent a very notable class of compounds in the field of medicinal chemistry. A variety of molecules containing this NAP scaffold were characterized to exhibit several pharmacological properties beneficial for human health [1], including ability to effectively combat infections caused by specific mycobacterial strains. In fact, the precisely designed and structurally optimized framework of these so-called privileged structures that contained various polar functional group(s), including an ester, (substituted) amino, (substituted) amido or convenient heterocyclic fragments(s), as well as (precisely chosen) *N*-(substituted phenyl)piperazin-1-yl moieties, allowed for several of them to be considered highly efficient antimycobacterial agents. Following particular in vitro experimental evaluations, some of such designed compounds were described as more active antimycobacterial agents or showed more favorable values of a selectivity index compared to potency or selectivity of first-line antituberculosis (anti-TB) drugs represented by isoniazid (isonicotinic acid hydrazide; INH), ethambutol as a (substituted) ethylenediamine-based derivative or rifampicin (RIF), a drug belonging to an ansamycin class of antibiotics [2,3,4,5].

The ester group of 1-(3-{4-[(alkoxycarbonyl)amino]benzoyloxy}-2-hydroxypropyl)-4-(substituted phenyl)piperazin-1-ium chlorides (**1**)–(**12**) (alkoxy = methoxy to butoxy) was not preliminarily supposed to be a structural factor that would compromise their in vitro antimycobacterial properties [6] (Figure 1). Several derivatives from this series (Section A, Table S1; Supplementary Materials) relatively effectively combat *Mycobacterium tuberculosis* CNCTC My 331/88 which was identical to the H_37_R_v_ (abbreviation used: *Mtb* H_37_R_v_) and ATCC 2794 strains, *M. kansasii* CNCTC My 235/80 (identical to ATCC 12478; *MK* My 235/80), *M. kansasii* 6509/96 clinical isolate (*MK*_ci_ 6509/96) and *M. avium* CNCTC My 330/80 (identical to ATCC 25291; *MA* My 330/80), respectively, under in vitro experimental conditions. 1-(3-{4-[(Butoxycarbonyl)amino]benzoyloxy}-2-hydroxypropyl)-4-(2-methoxyphenyl)piperazin-1-ium chloride (**12**), as the most prospective antimycobacterial agent from the set (**1**)–(**12**) (Figure 1), inhibited growth of *Mtb* H_37_R_v_ at a minimum inhibitory concentration (*MIC*) of 16 μM [6]. On the other hand, its efficacy was several times lower compared to activity of INH (*MIC* = 0.5 μM; Table S1).

A study [7] concluded that introduction of halogens on an aromatic ring within a 4-(2-/3-/4-*R*-substituted phenyl)piperazin-1-yl moiety (*R* = OCH_3_ or halogen) (Figure 2) would be rather questionable in an attempt to achieve effective antimycobacterial agents. The results of in vitro antimycobacterial evaluation of such *R*-substituted molecules were supported by a relevant in silico analysis. These naphthalene–NAP hybrid compounds contained some structural motifs as the molecules [6] mentioned above (Figure 1).

**Figure 1:**
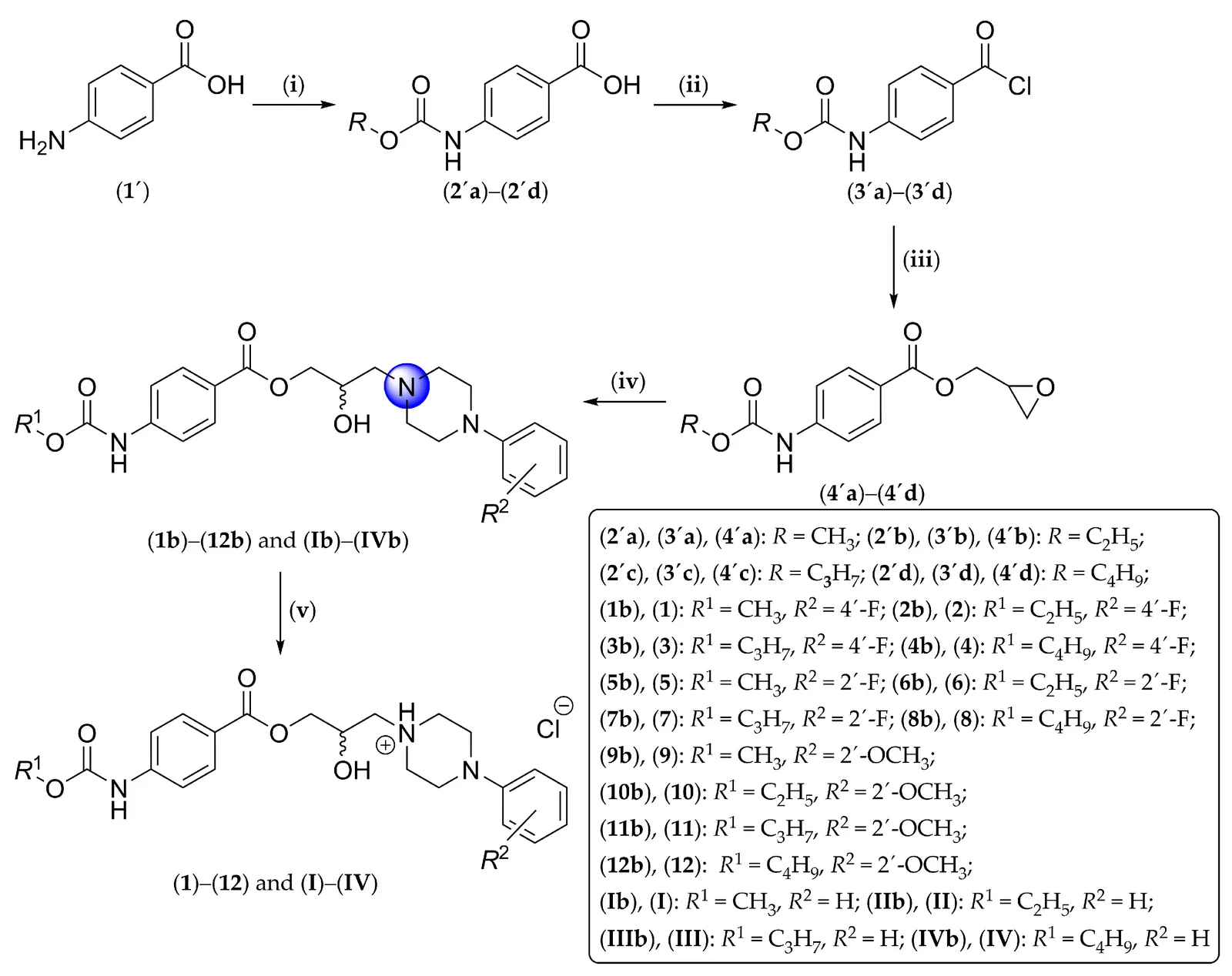
Synthesis of presently investigated arylcarbonyloxypiperazinopropanols, that is, non-protonated derivatives (**Ib**)–(**IVb**) and (**1b**)–(**12b**), as well as their salts (**I**)–(**IV**) and (**1**)–(**12**). Reagents and conditions: (i) addition of particular alkyl chloroformates (alkyl = methyl to butyl; *n* = 0.10 mol), isolation of 4-[(alkoxycarbonyl)amino]benzoic acids (**2′a**)–(**2′d**) (alkoxy = methoxy to butoxy), crystallization from ethanol 96%; (ii) thionyl chloride (*n* = 0.20 mol) gradually added to individual suspensions of (**2′a**)–(**2′d**) in toluene (*V* = 120 mL), heating (2 h) under reflux, isolation of alkyl 4-[(chlorocarbonyl)phenyl]carbamates (**3′a**)–(**3′d**) (alkyl = methyl to butyl), crystallization from toluene; (iii) intermediates (**3′a**)–(**3′d**) (*n* = 0.25 mol) dissolved in tetrahydrofuran (THF; *V* = 250 mL) and cooled to −5 °C, particular solutions added dropwise at −5 °C to a mixture, consisting of (±)-(oxiran-2-yl)methanol and triethylamine (TEA; *n* = 0.05 mol) dissolved in THF (*V* = 50 mL). Respective solutions were stirred (3 h), formed precipitate was filtered, (±)-(oxiran-2-yl)methyl 4-[(alkoxycarbonyl)amino]benzoates (**4′a**)–(**4′d**) (alkoxy = methoxy to butoxy) were isolated, crystallization from propan-2-ol; (iv) individual mixtures, consisting of (**4′a**)–(**4′d**) (*n* = 0.25 mol) and particular basic secondary amines (*n* = 0.25 mol), that is, 1-(4-fluorophenyl)piperazine, 1-(2-fluorophenyl)piperazine, 1-(2-methoxyphenyl)piperazine and 1-phenylpiperazine, in propan-2-ol (*V* = 150 mL) were heated (4 h) at 80 °C, oily compounds (**1b**)–(**12b**) and (**Ib**)–(**IVb**) were isolated; (v) individual derivatives (**1b**)–(**12b**) and (**Ib**)–(**IVb**) dissolved in diethyl ether, addition of an etheric solution of hydrochloric acid provided their chloride salts (**1**)–(**12**) and (**I**)–(**IV**), crystallization from particular alcohol [8,9]. The possibility to form respective enantiomers (considering the spatial arrangement of substituents attached to a stereogenic center (*C*-atom)) is indicated by a wavy bond. The *N*-atom that was chosen to be protonated in current in silico analyses is color-coded.

**Figure 2:**
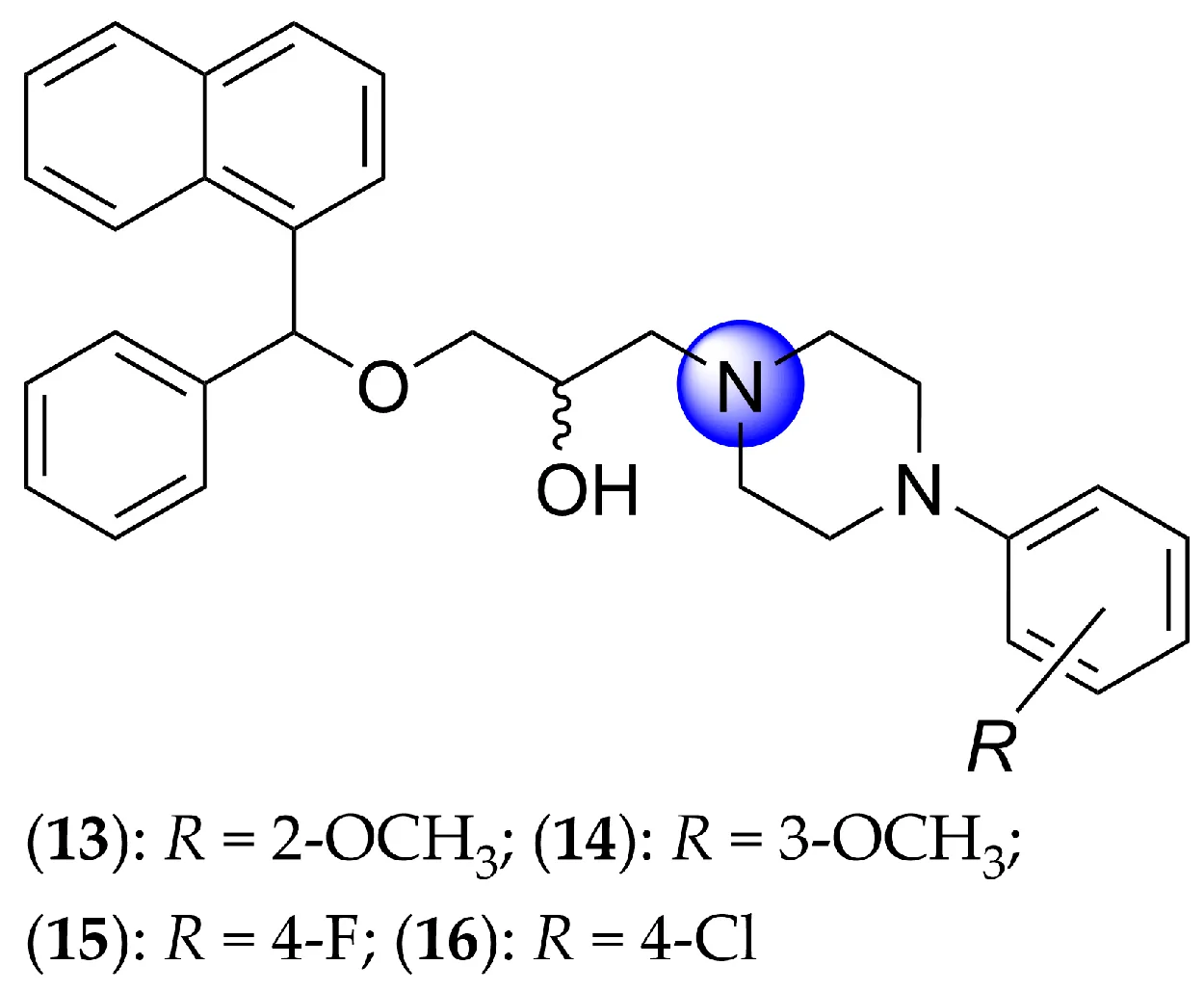
Chemical structure of naphthalene–NAP hybrid derivatives (**13**)–(**16**) screened in vitro against a virulent *Mtb* H_37_R_v_ strain [7]. The possibility to form respective enantiomers (considering the spatial arrangement of substituents attached to a stereogenic center (*C*-atom)) is indicated by a wavy bond. The *N*-atom that was chosen to be protonated in current in silico analyses is color-coded.

Upadhayaya et al. [7] also observed that both OCH_3_-substituted derivatives (**13**) and (**14**) combatted *Mtb* H_37_R_v_ more efficiently (Figure 2) than the 4-halogen substituted molecule (**15**) or (**16**) (halogen = F or Cl). Similarly, the replacement of the 4-(2-/3-/4-*R*-substituted phenyl)piperazin-1-yl core with 4-(2-pyridyl)piperazin-1-yl caused the loss of ability of a resulting compound to effectively inhibit growth of a given mycobacterial strain under conditions in vitro [7].

Taking all these observations in mind, one objective of the current research was to carry out the in vitro antimycobacterial screening of 1-(3-{4-[(alkoxycarbonyl)amino]benzoyloxy}-2-hydroxypropyl)-4-phenylpiperazin-1-ium chlorides (**I**)–(**IV**) (alkoxy = methoxy to butoxy) (Figure 1). This evaluation would contribute to complex investigation of the impact of substituents’ properties attached to an aromatic portion within a 4-(substituted phenyl)piperazin-1-yl fragment, as present in both series (**I**)–(**IV**) and (**1**)–(**12**), on their antimycobacterial efficiency. The synthesis of the derivatives (**I**)–(**IV**) (Figure 1), that were presently tested against *Mtb* H_37_R_v_, *MK* My 235/80, *MK*_ci_ 6509/96 and *MA* My 330/80, respectively, was already published [8,9].

Prior to in vitro evaluation of the compounds (**I**)–(**IV**), 2-hydroxy-3-(4-phenylpiperazin-1-yl)propyl 4-[(alkoxycarbonyl)amino]benzoates (**Ib**)–(**IVb**) (alkoxy = methoxy to butoxy), as non-protonated derivatives (Brønsted–Lowry bases) of these salts (Figure 1), were subjected to in silico characterization, employing various interactive web-based engines, namely, SwissADME [10], pkCSM [11], MolGpKa [12], DataWarrior ver. 06.05.04 [13] and Molsoft L.L.C. [14], respectively, together with the standalone ChemDraw ver. 22.2.0.3300 software package (Table 1 and Table 2). The differences in antimycobacterial activity of respective derivatives within the series (**I**)–(**IV**), that might be expected, could be closely connected with their different structural, physicochemical and pharmacokinetic properties. In fact, these characteristics of particular non-protonated compounds could be reflected in the features of individual salts.

The current research also involved the in silico analysis of the molecules (**1b**)–(**12b**) [6], as well as the derivatives (**13**) and (**14**) [7] (Table 1 and Table 2, Figure 1 and Figure 2) considering respective characteristics mentioned above. The reason for the selection of both (**13**) and (**14**) was that these molecules were relatively more or less structurally similar to (**Ib**)–(**IVb**) and (**1b**)–(**12b**). The estimated *MIC*s [6] for the set (**1**)–(**12**) can be found in Table S1 and *MIC* values determined in vitro for both molecules (**13**) and (**14**) against *Mtb* H_37_R_v_ were 12.95 μM (6.25 μg/mL) as listed in [7].

Molecular docking (MD) analyses were also carried out using the Small-Molecule Discovery Suite ver. 14.7 software package (Schrödinger Inc., New York, NY, USA), mainly concerning the most effective antimycobacterial agents from both sets (**I**)–(**IV**) and (**1**)–(**12**). In fact, the in silico evaluation of the naphthalene–NAP hybrids (Figure 2) [7] was inspiration for the direction of the current research to explore possible interactions between these small-molecule ligands and adenosine 5′-triphosphate (ATP) synthase in *Mtb* H_37_R_v_. This enzyme would be a very prospective biological target for the presently screened derivatives with the aim to effectively combat infections caused by a given slow-growing pathogen [15].

In addition, the antimycobacterially most promising compounds from both sets (**I**)–(**IV**) and (**1**)–(**12**) as well as INH reference drug were investigated in vitro to evaluate their antiproliferative and potential cytotoxic effects on normal human dermal fibroblasts.

Finally, in silico analyses were also performed to ‘roughly’ predict the possibilities of whether the non-protonated compounds and (indirectly) corresponding salts might effectively act against a broader spectrum of mycobacterial species.

## 2. Materials and Methods

### 2.1. Synthesis and Several Physicochemical Properties of the Compounds (***I***)–(***IV***) and (***1***)–(***12***)

The synthetic procedures providing non-protonated derivatives (**Ib**)–(**IVb**) as well as respective salts (**I**)–(**IV**), prepared as racemates (Figure 1), can be found in [8,9] together with their nuclear magnetic resonance (^1^H NMR and ^13^C NMR) and infrared (IR) spectral characterization. More detailed information connected with particular reagents and reaction conditions, as well as ^1^H NMR, ^13^C NMR and IR spectral data of individual salts, can be found in Section B (Supplementary Materials). Suitable descriptors that defined some fundamental physicochemical properties of (**I**)–(**IV**), that is, melting point (mp) values, solubility in various solvents, surface activity, electronic (spectroscopic electronic transition), acid–base features (described by values of their dissociation constant (p*K*_a_) parameter) and lipophilic characteristics (described by values of decadic logarithms of a retention (capacity) factor *k* (log *k*) estimated through a reversed-phase high-performance liquid chromatography method), were estimated as well [16]. The values of chosen parameters are summarized in Table S2 (Supplementary Materials).

The synthesis (Figure 1) of non-protonated 3-[4-(4-fluoro-/2-fluoro-/2-methoxyphenyl)piperazin-1-yl]-2-hydroxypropyl 4-[(alkoxycarbonyl)amino]benzoates (**1b**)–(**12b**) (alkoxy = methoxy to butoxy) and their salts (**1**)–(**12**) was already published [17,18] together with IR spectral characterization and elemental analysis data for (**1**)–(**12**). More detailed information related to particular reagents and reaction conditions, as well as the ^1^H NMR, ^13^C NMR and IR spectral outputs of final derivatives, can be found in Section B (Supplementary Materials). Several physicochemical characteristics of (**1**)–(**12**) were also experimentally determined, that is, mps, solubility in distilled water, p*K*_a_ data and values of the log *k* parameter [17,18]. The values of chosen parameters are summarized in Table S2.

The multi-step preparation of final compounds (**1**)–(**12**) and (**I**)–(**IV**) (Figure 1) was regioselective. Specifically concerning the syntheses leading to non-protonated derivatives (**Ib**)–(**IVb**) and (**1b**)–(**12b**) from individual (±)-(oxiran-2-yl)methyl 4-[(alkoxycarbonyl)amino]benzoates (**4′a**)–(**4′d**) (alkoxy = methoxy to butoxy) and respective basic amines, the sterically demanding 1-(substituted phenyl)piperazines attacked, as nucleophiles, a less hindered terminal *C*-atom of a CH_2_-moiety of an oxiran-2-yl (so-called non-symmetrically substituted ‘epoxide’) ring within a structure of (**4′a**)–(**4′d**) [19] under basic conditions (here, TEA was used). Additionally, the regioselectivity of such nucleophilic substitution reactions was also observed in other research, aiming at preparation and evaluation of derivatives belonging to various pharmacodynamic classes [7,20,21,22].

The experimental ^1^H NMR, ^13^C NMR and IR spectral values of the compounds (**I**)–(**IV**) and (**1**)–(**12**) were consistent with their proposed structure.

The reason to employ different numerals (Arabic and Latin) for labeling of investigated arylcarbonyloxypiperazinopropanols (**I**)–(**IV**) and (**1**)–(**12**) was to distinguish them (and their non-protonated forms as well) according to whether or not they were already antimycobacterially screened. Thus, the evaluations of particular structural, physicochemical, pharmacokinetic and pharmacodynamic properties of non-protonated compounds (**Ib**)–(**IVb**) and their salts (**I**)–(**IV**) are listed before those of (**1b**)–(**12b**) and (**1**)–(**12**). More details can be found in Section B (Supplementary Materials).

### 2.2. In Silico Investigation of Non-Protonated Molecules (***Ib***)–(***IVb***), (***1b***)–(***12b***), (***13***) and (***14***)—Prediction of Some Structural and Physicochemical Properties

The freely available SwissADME interactive tool [10] was employed to generate required linear notations of particular Simplified Molecular Input Line Entry System (SMILES) codes [23] for the derivatives (**Ib**)–(**IVb**), (**1b**)–(**12b**), (**13**) and (**14**) (Figure 1 and Figure 2). These codes are provided in Table S3 (Supplementary Materials). The same interactive engine together with the DataWarrior ver. 06.05.04 software [13] was used to predict several fundamental structural and physicochemical characteristics of all of these molecules.

The calculated descriptors (Table 1 and Table 2) were as follows: formula, molecular weight (*MW*; expressed in g/mol units), fraction of *sp*^3^-hybridized *C*-atoms (F*sp*^3^), number of rotatable bonds (*n*_rotb_), number of hydrogen-bond acceptors (*n*_ON_), number of hydrogen-bond donors (*n*_OHNH_), total surface area (*TSA*; in Å^2^ units), topological polar surface area (*tPSA*; in Å^2^ units), molecular flexibility (*MolFlex*) calculated as a weighted rotatable-bond model with implementation of several structural corrections, molar refractivity (*MR*; in m^3^/mol units), as well as a very well-known lipophilicity parameter, a decadic logarithm of a partition coefficient (log *P*) calculated for an octan-1-ol/water partition system via various methods, namely, XLOGP3 [24], WLOGP [25], and MLOGP [26].

The log *P* values of individual non-protonated derivatives were also predicted considering the same partition system through a CLOGP algorithm [27] that was implemented in the environment of the commercially available standalone ChemDraw ver. 22.2.0.3300 software package (CambridgeSoft, Cambridge, MA, USA) presently used as a trial version. In addition, lipophilicity of (**Ib**)–(**IVb**), (**1b**)–(**12b**), (**13**) and (**14**) described by their log *P*_pkCSM_ values and MolLogP readouts were calculated through the freely accessible web-based pkCSM engine [11] and Molsoft L.L.C. applet (Molsoft L.L.C., San Diego, CA, USA), respectively. The fundamental characteristics of the latter interactive tool were briefly provided in [14].

The acid–base properties of non-protonated molecules (**Ia**)–(**IVb**), (**1b**)–(**12b**), (**13**) and (**14**), described by values of their p*K*_a_, were also predicted using a freely available MolGpKa platform [12]. The data obtained for (**Ib**)–(**IVb**) and (**1b**)–(**12b**) (Table 2) were compared to those that were determined previously for individual salts from both sets (**I**)–(**IV**) [16] and (**1**)–(**12**) [17,18]. The values of experimental p*K*_a_ descriptors are listed in Table S2. Molecular volume of respective non-protonated derivatives was characterized by their *MolVol* parameters (in Å^3^ units) calculated via the Molsoft L.L.C. applet [14].

Brief definitions of particular structural and physicochemical descriptors and their contribution to drug-like profiles [28] of biologically effective compounds were provided in [29].

### 2.3. In Silico Investigation of Non-Protonated Molecules (***Ib***)–(***IVb***), (***1b***)–(***12b***), (***13***) and (***14***)—Prediction of Some Pharmacokinetic Properties

The web-based pkCSM applet [11] was employed to predict the extent of intestinal absorption (IA parameter; in percentages) for analyzed compounds (**Ib**)–(**IVb**), (**1b**)–(**12b**), (**13**) and (**14**) in humans, as well as their ability to cross via human intestinal cells (Table 3). In the latter case, a theoretical Caco-2 cell model was used to evaluate the absorption of compounds after their administration per os. This feature was described by a decadic logarithm of an apparent permeability coefficient (*P*_app_; values of *P*_app_ were expressed in 10^−6^ cm/s units).

A molecule with absorption (IA) of <30.00% was regarded as poorly absorbed, a compound showing absorption (IA) in a range of 30.00–80.00% was considered moderately absorbed and a derivative with absorption (IA) of >80.00% showed high intestinal absorption extent. Low intestinal permeability of a compound was considered if its log *P*_app_ was < 0.000, that is, if *P*_app_ < 1.00 × 10^−6^ cm/s. The log *P*_app_ value within an interval of 0.000–0.900 indicated that a derivative showed moderate intestinal permeability. Finally, high intestinal permeability of a compound was considered if its log *P*_app_ was >0.900, that is, if *P*_app_ > 8.00 × 10^−6^ cm/s [11].

The SwissADME [10] and pkCSM [11] platforms were used to predict some distribution characteristics of (**Ib**)–(**IVb**), (**1b**)–(**12b**), (**13**) and (**14**), that is, whether they would be able to passively permeate through the blood–brain barrier (BBB). In general, the algorithm implemented within pkCSM [11] categorized the compounds as highly permeable that were characterized by a logarithmic value of blood-to-brain ratio (log BB) > 0.300, moderately permeable by −1.000 < log BB < 0.300 and, finally, poorly permeable by log BB < −1.000.

The central nervous system (CNS) permeability, as another distribution feature, was also predicted (Table 3) according to a permeability–surface area product (log PS) parameter. The compounds able to passively permeate had log PS > −2.000, the molecules with notably limited or no ability to permeate the CNS showed log PS < −3.000 [11].

The freely accessible GLORYx applet [30] was used to predict relevant biotransformation pathways of evaluated derivatives (**Ib**)–(**IVb**) and (**1b**)–(**12b**). This interactive tool provided a palette of metabolites that could be formed in humans in phase I and phase II metabolism (Table S4; Supplementary Materials). Particular predictions were scored and ranked (per input molecule) on the basis of predicted sites of biotransformation probabilities of atoms involved in an appropriate reaction and a simple binary weighting of a corresponding reaction type. The SMILES codes (Table S1) necessary for that kind of analysis of all these non-protonated molecules were generated through the SwissADME tool [10].

The overall quantitative estimate of drug-likeness (*QED* parameter) was calculated to globally evaluate the extent of absorption (passing of a derivative from an area of administration into the bloodstream) and bioavailability (degree and rate at which a derivative was absorbed into the bloodstream) of investigated compounds [31]. The calculation of this parameter was carried out through the freely available interactive web-based DruglikeFilter ver. 1.0 applet [32] and was based on several descriptors, namely, *MW*, ALOGP, as a method of lipophilicity prediction of a compound for the octanol-1-ol/water partitioning system [33], *n*_ON_, *n*_OHNH_, *tPSA*, *n*_rotb_, number of aromatic rings and number of so-called structural alerts (toxicophores). The alerts were various functional groups or fragments (eventually) present in the structure of pharmacologically active compounds (drugs) that were connected with adverse drug reactions.

### 2.4. Relationships Between Structural, Physicochemical and Pharmacokinetic Parameters of Non-Protonated Molecules (***Ib***)–(***IVb***) and (***1b***)–(***12b***)

A more detailed view on relationships between values of particular parameters characterizing structural, physicochemical and pharmacokinetic properties of the derivatives (**Ib**)–(**IVb**) and (**1b**)–(**12b**) was provided, employing a principal component analysis (PCA) method [34,35] in order to reduce the dimensionality of investigated data while maintaining most of the variability in values. This chemometric technique was performed using a trial version of the XLSTAT ver. 2019 2.2 statistical software (Addinsoft, New York, NY, USA). The given add-in worked in the Microsoft Excel environment integrated within the Microsoft 365 software package (Microsoft Corp., Redmont, WA, USA). The details about principles of PCA with brief definitions and explanations of significance of several fundamental terms and parameters, that is, principal components (PCs), pretreatment methods, scree plots, eigenvalues (λ_e_), circle of correlation and Pearson’s correlation coefficient (*r*), which are valid for linear regression analyses, can be found in [29,34,35].

Similarities or differences in these descriptors were visualized in a constructed coordinate system of relevant PCs. In the present analyses, particular PCs did not prove the existence of ‘real’ structural, physicochemical or pharmacokinetic parameters, in fact. These components only indicated a mathematical possibility that such descriptors existed.

The values of *λ*_e_ parameters [34] for individual PCs were calculated and properly selected with respect to the Kaiser–Guttman rule as the most common stopping rule [36] in PCA. The purpose of using that approach was to limit the number of PCs and select the relevant ones. Only PCs defined by *λ*_e_ > 1.00 might be taken into consideration. The relationships between particular structural, physicochemical and pharmacokinetic descriptors defined by the *r* values are provided in Table S5 (Supplementary Materials).

### 2.5. In Vitro Screening of the Compounds (***I***)–(***IV***) Against Particular Mycobaterial Strains

The in vitro efficacy of the derivatives (**I**)–(**IV**) was presently evaluated against drug-sensitive virulent *Mtb* H_37_R_v_ (dilution of this strain was 10^−3^), *MK* My 235/80, *MK*_ci_ 6509/96 (dilution of both strains: 10^−4^) and *MA* My 330/80 (dilution of this strain: 10^−5^), respectively, in the Laboratory for Diagnostic of Mycobacterial Infections and Tuberculosis (Public Health Institute in Ostrava, Czech Republic). The evaluations were carried out following the procedures described in [37].

In particular determinations, INH, as a first-line anti-TB drug, and/or amikacin (AMK), a second-line anti-TB treatment option, were used as reference drugs. Both compounds were available in the Public Health Institute in Ostrava. The dilutions of respective mycobacterial strains, when investigating their susceptibility toward the activity of INH and AMK, were the same as in the case of experiments involving (**I**)–(**IV**).

Incubation periods were established in the current screening as follows: 14 days (abbreviation used: 14-d) and 21 days (21-d) for *Mtb* H_37_R_v_ and *MA* My 330/80, as well as 7 days (7-d), 14 days and 21 days for both tested *MK* strains. All of these incubations were carried out at 37 °C.

The individual values of the *MIC* parameter were determined as the lowest concentration of a compound, leading to complete inhibition of growth of tested mycobacterial strains [37] (Table 4). The evaluations for particular derivatives were performed in triplicate and the obtained results, that is, estimated *MIC* data, were the same.

### 2.6. In Silico Investigation of Non-Protonated Molecules (***Ib***)–(***IVb***) and (***1b***)–(***12b***), as Well as Their Salts (***I***)–(***IV***) and (***1***)–(***12***)—Molecular Docking

The purchased Small-Molecule Discovery Suite ver. 14.7 software package (Schrödinger Inc., New York, NY, USA) was employed to inspect and characterize eventual interactions between analyzed non-protonated compounds (**Ib**)–(**IVb**) and (**1b**)–(**12b**), as well as particular salts (**I**)–(**IV**) and (**1**)–(**12**) and their proposed biological target, ATP synthase. This enzyme is present in various mycobacteria including *Mtb* H_37_R_v_. The software package contained several modules, namely, Maestro (release 2022-3), LigPrep, Protein Preparation Wizard, Epik, PROPKA ver. 2.0, Sitemap and Glide (release 2022-3), respectively, using a Prime Molecular Mechanics–Generalized Born Surface Area (Prime MM-GBSA) method of binding energy calculations [38,39,40,41].

The three-dimensional (3D) crystal structure of ATP synthase, that is, its *a* and *c* (AtpE) subunits [7], was obtained from the Protein Data Bank (code name of this enzyme: 8J57). Protein preparation was performed using the Protein Preparation Wizard model with simulation carried out at p*H* = 7.4. In a preprocess, all missing side chains were filled, bond orders were assigned, hydrogen atoms were replaced, zero-order bonds to metals and disulfide bonds were created and heteroatom states were generated with Epik [42] at p*H* = 7.4 ± 2.0. The hydrogen bond orders were optimized through simple water orientations, considering all ligand states. For optimization, the PROPKA ver. 2.0 [43] module was used and all atoms were minimized to a max root mean square deviation of 0.3 Å. In addition, water molecules were removed within the distance of 5 Å from individual ligands in an Optimized Potentials for Liquid Simulations 4th generation (OPLS4) force field [44].

The two-dimensional (2D) structures of (**Ib**)–(**IVb**), (**1b**)–(**12b**), (**I**)–(**IV**) and (**1**)–(**12**) were obtained using the Maestro’s 2D Sketcher tool [45] ensuring their correct 2D representation. The LigPrep module [46] as a convenient virtual environment for structure-based screening was used for the preparation of these ligands. The respective 2D structures were successfully converted into corresponding 3D forms with each ligand consisting of more conformation and protonation states in space. These states were generated using a maximum ligand size of 500 atoms in the OPLS4 force field.

For ionization, possible states were generated at target p*H* = 7.0 ± 2.0 using the Epik Classic interactive environment [47] with the option of desalting and generating tautomers. The individual (*R*)- and (*S*)-stereoisomers of these compounds were computed.

The Sitemap platform [48] was used to determine (top-ranked) potential binding sites. In these calculations, at least 15 site points per reported site were set up, the report was up to 5 sites. A more restrictive definition of hydrophobicity and a standard grid were used. Crop site maps were 4 Å from the nearest site point and an attempt was made to subdivide sites larger than 800 Å.

The SiteScore and DScore metrics [49] were evaluated and, if applicable, the binding site as the highest scoring site representing a grid center was chosen. Interaction potentials of the protein binding site were precomputed to generate a grid-based energy map that Glide [50] used to rapidly score ligand conformations during the MD procedure.

The receptor grid was generated using the Glide grid generation tool [50]. The grid box dimensions were set so the binding site generated in a previous step was at the center of an inner and outer box. In addition, there was sufficient surrounding space for a ligand to rotate. The binding site was employed as a center of the grid box supplied 30 × 30 × 30 Å for an outer box and 15 × 15 × 15 Å for an inner box. The length of particular ligands was settled at 20 Å.

The docking calculations were carried out using the Glide module [50] as well. The prepared ligands were inserted into a pregenerated receptor grid binding site using simple precision and flexible ligand sampling methods. The postdocking minimization for up to 5 poses per ligand was also performed. The key interactions between individual ligands and amino acid (AA) residues in a binding pocket, such as hydrogen bonds, hydrophobic interactions, *π*–*π* or salt-bridge (ionic) interactions, were visually investigated within a postdocking evaluation process.

Binding energies, that is, the values of the MM-GBSA ΔG Bind parameter (in kcal/mol units) of individual ligands ((*R*)- and (*S*)-stereoisomeric forms of non-protonated derivatives as well as their salts) docked into a structure of ATP synthase were calculated by a Prime MM-GBSA method [51] (Tables S6 and S7; Supplementary Materials). Protein flexibility was measured as the 5 Å distance from a ligand in a variable surface generalized born solvatation model [52] and OPLS4 force field [44]. The sampling method was employed to minimize individual outputs.

The strength of ligands binding to their binding site was evaluated following the calculated GBSA energy values. More negative binding energies indicated stronger interactions between investigated ligands and ATP synthase.

Docking score values (in kcal/mol units) of individual ligands with defined stereochemical properties were generated as well (Tables S8 and S9; Supplementary Materials). More negative values indicated higher stability of interactions and, thus, a higher likelihood that such predicted binding interactions would be formed [53].

Descriptions of particular interactions formed between atoms and groups in a structure of evaluated ligands and interacting AA residues can be found in Table S10 (Supplementary Materials). A more detailed view on interactions that were formed between the most effective anti-*Mtb* H_37_R_v_ derivatives (more specifically, their stereoisomers) and interacting AA residues, as well as lengths (in Å units) of these non-covalent interactions, is provided in Table S11 (Supplementary Materials).

### 2.7. In Vitro Antiproliferative Evaluation of Chosen Compounds

The two most efficient antimycobacterial compounds from both sets (**I**)–(**IV**) and (**1**)–(**12**), that is, one derivative from each series, as well as an INH reference drug, were chosen to evaluate their antiproliferative and cytotoxic effects in normal human dermal fibroblasts through the colorimetric cell viability assay using a 3-(4,5-dimethylthiazol-2-yl)-2,5-diphenyl-2*H*-tetrazolium bromide (MTT) reagent [54].

The cells (Merck, Bratislava, Slovakia) were cultured at 37 °C in 5% CO_2_ atmosphere in Dulbecco’s modified Eagle medium (DMEM; Merck), supplemented by 10% heat-inactivated fetal bovine serum (Sigma, Schnelldorf, Germany), 1% penicillin/streptomycin and 4 mM L-glutamine. The cells were seeded at 1 × 10^4^ per well in 96-well microplates (Greiner Bio-One GmbH, Frickenhausen, Germany) and incubated for 24 h under standard conditions. After incubation, the medium was removed and cells were exposed to varying compound concentrations (*c*s) of 7.8, 15.6, 31.3, 62.5, 125 and 250 μmol/mL (in DMEM) for 24 h.

The stock solutions of evaluated NAPs were prepared in dimethyl sulfoxide (Merck). The concentration of this solvent in the incubation solution did not exceed 0.0125%.

The MTT assay based on the reduction of the MTT reagent (Merck) to formazan by the activity of intracellular dehydrogenases in metabolically active cells was used to assess antiproliferative properties of tested derivatives. After incubation, the medium was replaced with 100 μL of the MTT solution in DMEM (0.5 mg/mL). After 2 h at 37 °C in 5% CO_2_, 100 μL of a solubilization solution (consisting of 10% sodium dodecyl sulfate in 0.01 M HCl) was added and cells were incubated overnight at 37 °C to fully dissolve the dye.

Representative images of the compound-treated cells incubated with MTT were taken before the addition of a solubilization reagent using the Olympus CKX41 inverted phase-contrast microscope (Olympus, Tokyo, Japan) equipped with the QuickPHOTO Micro 3.1 software (Promicra, Prague, Czech Republic). A Bürker chamber was used to estimate the size of the scale bar (200 mm).

Optical density (OD) was measured at a wavelength (*λ*) of 570 nm using the Tecan Infinite 200 Pro reader (Tecan AG, Grödig/Salzburg, Austria), with *λ* = 690 nm as a reference. Cell viability (viability rate; in percentages) was calculated according to Equation (1):

(1) $$Viability\;rate\;(\%)=\;\left(\frac{{OD}_{treated}}{{OD}_{control}}\right)\times 100,$$

where the OD_treated_ and OD_control_ parameters represent optical density (OD) values related to treated cells and non-treated cells, respectively.

Statistical analyses of these evaluations were carried out using the IBM SPSS software ver. 22.0 (SPSS Inc., Chicago, IL, USA). Means of experiments in triplicate were calculated and results were expressed as means ± standard deviation (SD). Data homogeneity was checked with Levene’s test. Homogeneous data were analyzed using analysis of variance (ANOVA) and a post hoc Dunnett’s *t*-test [55] against the control. For non-homogeneous data, ANOVA with post hoc Dunnett’s T3 test [56] was used. Significance levels were indicated by asterisks, that is, * *p* < 0.05 as statistically significant, ** *p* < 0.01 as statistically very significant and *** *p* < 0.001 as extremely significant.

### 2.8. In Silico Investigation of Non-Protonated Molecules (***Ib***)–(***IVb***) and (***1b***)–(***12b***)—Inspection of Their Possibilities to Inhibit Growth of Particular Mycobacteria

The freely available web-based AntiBac-Pred applet [57] integrated in the PASS Online Predictor tool [58] was used to indicate the strains of mycobacteria which could be susceptible to the activity of (**Ib**)–(**IVb**) and (**1b**)–(**12b**). The SMILES codes (Table S1) necessary for that type of in silico evaluation of all these compounds were generated through the SwissADME tool [10].

The chances of the considered non-protonated molecules to affect particular mycobacterial species under experimental settings were described by a score parameter theoretically ranging from 0.0000 to 1.0000 (Table S12; Supplementary Materials). The higher the score, the greater the likelihood that a derivative would efficiently act against particular mycobacterial species under conditions in vitro [57].

## 3. Results

### 3.1. In Silico Investigation of Non-Protonated Molecules (***Ib***)–(***IVb***), (***1b***)–(***12b***), (***13***) and (***14***)—Prediction of Some Structural and Physicochemical Properties

The chemical structures of particular NAP-moiety-containing derivatives (**Ib**)–(**IVb**) and (**1b**)–(**12b**), as well as naphthalene–NAP hybrid molecules (**13**) and (**14**), were successfully transformed into corresponding SMILES codes [23] (Table S3) using the Marvin JS drawing tool effectively integrated in the environment of the SwissADME applet [10]. The successful completion of this process offered the possibility to evaluate all given compounds in silico. In fact, the correct forms of these codes were not generated for the molecules (**I**)–(**IV**) or (**1**)–(**12**), as (not only) this predictor tool did not properly consider positively and negatively charged derivatives. This observation was not brand new and corresponded to previous findings [59]. Therefore, the correctly created SMILES outputs (Table S3) for (**Ib**)–(**IVb**), (**1b**)–(**12b**), (**13**) and (**14**) allowed the relevant discussion regarding differences and similarities that might be expected in compounds’ structural and physicochemical characteristics.

When focusing on the derivatives (**Ib**)–(**IVb**), they differed in the values of *MW*, F*sp*^3^, *n*_rotb_, *TSA*, *MolFlex*, *MR* and *MolVol* descriptors (Table 1), as well as in the log *P* data generated through various predictive methods [11,14,24,25,26,27] (Table 2). The observed differences between these molecules were clearly connected to the nature (length) of their *R*^2^-substituent (Figure 1, Table 1). On the other hand, the same values of *n*_ON_ (7), *n*_OHNH_ (2), *tPSA* (91.34 Å^2^) and p*K*_a_ (6.8) parameters were calculated for (**Ib**)–(**IVb**).

The differences in generated log *P* data for (**Ib**)–(**IVb**), as a parameter describing their lipophilicity, confirmed that the length of the *R*^1^-group affected the values of a given physicochemical descriptor. Thus, the increase in *MW*s provided higher log *P*s as individual outputs from all used predictive algorithms shown (Table 2). Regarding particular methods that were used to calculate corresponding log *P*s of the compounds (**Ib**)–(**IVb**) for the octan-1-ol/water partition system, the lipophilic nature of these non-protonated derivatives decreased in a rank as follows (Table 2): CLOGP 4.0 (highest log *P* values were generated using this approach) > MolLogP > XLOGP3 > pkCSM > MLOGP > WLOGP (lowest log *P* values were generated using this approach).

The investigated derivatives might also be ranked according to average values of their log *P*s (log *P*_av_ data) calculated from particular log *P* outputs. In regard to these readouts, lipophilicity of (**Ib**)–(**IVb**) decreased as follows: (**IVb**), defined by log *P*_av_ = 3.51 > (**IIIb**), described by log *P*_av_ = 3.12 > (**IIb**), defined by log *P*_av_ = 2.63 > (**Ib**), described by log *P*_av_ = 2.25.

**Table 1:** Formula and several structural and physicochemical descriptors, characterizing the compounds (**Ib**)–(**IVb**), (**1b**)–(**12b**), (**13**) and (**14**) which were generated through the interactive SwissADME [10] and DataWarrior ver. 06.05.04 [13] tools.

^1^ Cmpd.	Formula	^3^ *MW* (g/mol)	^4^ F*sp*^3^	^5^ *n*_rotb_	^6^ *n*_ON_	^7^ *n*_OHNH_	^8^ *TSA* (Å^2^)	^9^ *MolFlex*	^10^ *MR* (m^3^/mol)
(**Ib**)	C_22_H_27_O_5_N_3_	413.47	0.36	10	6	2	320.75	0.5174	120.06
(**IIb**)	C_23_H_29_O_5_N_3_	427.49	0.39	11	6	2	334.51	0.5240	124.87
(**IIIb**)	C_24_H_31_O_5_N_3_	441.52	0.42	12	6	2	348.27	0.5190	129.68
(**IVb**)	C_25_H_33_O_5_N_3_	455.55	0.44	13	6	2	362.03	0.5192	134.49
(**1b**)	C_22_H_26_O_5_FN_3_	431.46	0.36	10	7	2	327.10	0.5195	120.02
(**2b**)	C_23_H_28_O_5_FN_3_	445.48	0.39	11	7	2	340.86	0.5216	124.83
(**3b**)	C_24_H_30_O_5_FN_3_	459.51	0.42	12	7	2	354.62	0.5239	129.64
(**4b**)	C_25_H_32_O_5_FN_3_	473.54	0.44	13	7	2	368.38	0.5240	134.44
(**5b**)	C_22_H_26_O_5_FN_3_	431.46	0.36	10	7	2	327.10	0.4882	120.02
(**6b**)	C_23_H_28_O_5_FN_3_	445.48	0.39	11	7	2	340.86	0.4922	124.83
(**7b**)	C_24_H_30_O_5_FN_3_	459.51	0.42	12	7	2	354.62	0.4962	129.64
(**8b**)	C_25_H_32_O_5_FN_3_	473.54	0.44	13	7	2	368.38	0.4978	134.44
(**9b**)	C_23_H_29_O_6_N_3_	443.49	0.39	11	7	2	343.01	0.4853	126.56
(**10b**)	C_24_H_31_O_6_N_3_	457.52	0.42	12	7	2	356.77	0.4893	131.36
(**11b**)	C_25_H_33_O_6_N_3_	471.55	0.44	13	7	2	370.53	0.4965	136.17
(**12b**)	C_26_H_35_O_6_N_3_	485.57	0.46	14	7	2	384.29	0.4980	140.98
^2^ (**13**)	C_31_H_34_O_3_N_2_	482.61	0.29	9	4	1	380.65	0.4244	152.34
^2^ (**14**)	C_31_H_34_O_3_N_2_	482.61	0.29	9	4	1	380.65	0.4574	152.34

^1^ Cmpd. = compound, ^2^ (**13**), (**14**) = both compounds were synthesized and screened in vitro in [7], ^3^ *MW* = molecular weight (expressed in g/mol units), ^4^ F*sp*^3^ = fraction of *sp*^3^-hybridized *C*-atoms, that is, number of *sp*^3^-hybridized *C*-atoms/total *C*-atoms count, ^5^ *n*_rotb_ = number of rotatable bonds, ^6^ *n*_ON_ = number of hydrogen-bond acceptors, ^7^ *n*_OHNH_ = number of hydrogen-bond donors, ^8^ *TSA* = total surface area (in Å^2^ units), ^9^ *MolFlex* = molecular flexibility, ^10^ *MR* = molar refractivity (in m^3^/mol units).

**Table 2:** The values of a decadic logarithm of a partition coefficient (log *P*) of the compounds (**Ib**)–(**IVb**), (**1b**)–(**12b**), (**13**) and (**14**) for the octan-1-ol/water partition system predicted via the XLOGP3 [24], WLOGP [25], MLOGP [26], CLOGP 4.0 [27], MolLogP [14] and log *P*_pkCSM_ [11] methods, generated values of acid–base dissociation constant (p*K*_a_) and molecular volume (*MolVol*). Lipophilicity was predicted through the SwissADME interactive tool [10], integrating the XLOGP3, WLOGP and MLOGP predictive algorithms, as well as ChemDraw ver. 22.2.0.3300 software package (CLOGP 4.0; CambridgeSoft, Cambridge, MA, USA), Molsoft L.L.C. applet [14] and pkCSM engine [11]. The p*K*_a_ data were calculated through the virtual MolGpKa platform [12] and *MolVol* values were predicted via the Molsoft L.L.C. applet [14].

^1^ Cmpd.	^3^ XLOGP3	^4^ WLOGP	^5^ MLOGP	^6^ CLOGP 4.0	^7^ MolLogP	^8^ log *P*_pkCSM_	^9^ p*K*_a_	^10^ *MolVol* (Å^3^)
(**Ib**)	2.37	1.25	1.62	3.20	2.86	2.21	6.8	405.59
(**IIb**)	2.73	1.64	1.84	3.73	3.24	2.60	6.8	422.60
(**IIIb**)	3.26	2.03	2.05	4.26	4.12	2.99	6.8	441.00
(**IVb**)	3.62	2.42	2.26	4.79	4.56	3.38	6.8	458.91
(**1b**)	2.47	1.81	2.00	3.52	3.07	2.34	6.7	411.50
(**2b**)	2.83	2.20	2.21	4.05	3.45	2.73	6.7	428.51
(**3b**)	3.36	2.59	2.42	4.58	4.33	3.12	6.1	446.91
(**4b**)	3.72	2.98	2.63	5.11	4.77	3.51	6.1	464.82
(**5b**)	2.47	1.81	2.00	3.52	3.04	2.34	6.6	411.42
(**6b**)	2.83	2.20	2.21	4.05	3.41	2.73	6.6	428.43
(**7b**)	3.36	2.59	2.42	4.58	4.30	3.12	6.6	446.83
(**8b**)	3.72	2.98	2.63	5.11	4.73	3.51	6.6	464.73
(**9b**)	2.34	1.26	1.33	3.23	2.96	2.21	6.9	437.72
(**10b**)	2.71	1.65	1.64	3.76	3.33	2.60	6.9	454.73
(**11b**)	3.23	2.04	1.75	4.29	4.22	2.99	6.9	473.13
(**12b**)	3.59	2.43	1.95	4.81	4.66	3.39	6.9	491.04
^2^ (**13**)	5.50	4.05	3.29	5.73	5.70	5.14	6.9	480.86
^2^ (**14**)	5.50	4.05	3.29	5.73	5.65	5.14	6.9	480.65

^1^ Cmpd. = compound, ^2^ (**13**), (**14**) = both compounds were synthesized and screened in vitro in [7], ^3^ XLOGP3 = lipophilicity parameter (log *P*) calculated through an XLOGP3 atomic/group-based method [24], ^4^ WLOGP = lipophilicity parameter (log *P*) calculated via Wildman and Crippen’s atomic-based method [25], ^5^ MLOGP = lipophilicity parameter (log *P*) calculated through Moriguchi’s method [26], ^6^ CLOGP 4.0 = lipophilicity parameter (log *P*) calculated via a fragmental CLOGP 4.0 method [27], ^7^ MolLogP = lipophilicity parameter (log *P*) calculated through a fragment-based method considering a partial least squares regression approach [14], ^8^ log *P*_pkCSM_ = lipophilicity parameter (log *P*) calculated via a graph-based predictive machine learning approach [11], ^9^ p*K*_a_ = acid–base dissociation constant parameter predicted via the MolGpKa web server [12], ^10^ *MolVol* = molecular volume (in Å^3^ units) generated via the Molsoft L.L.C. applet [14].

The compounds (**I**)–(**IV**) were very slightly soluble [60] in distilled water. Therefore, it was not possible to experimentally determine their log *P* parameters through a classical shake-flask method because this evaluation required complete dissolution of investigated compounds in an aqueous phase [16]. Thus, direct comparison of theoretical log *P*_av_ values related to (**Ib**)–(**IVb**) with those experimentally determined for (**I**)–(**IV**) was not possible.

The nature of *R*^2^ (Figure 1) did not cause differences in the values of p*K*_a_ calculated for particular derivatives (**Ib**)–(**IVb**); p*K*_a_ = 6.2 was generated for all of them. These values indicated less acidic properties of (**Ib**)–(**IVb**) compared to acid–base characteristics of corresponding salts [16] whose experimentally estimated p*K*_a_s ranged from 4.6, as observed for (**II**), to 5.0, connected with (**III**) (Table S2). The differences between calculated and estimated data related to individual non-protonated compounds and corresponding salts were approximately 1.0–1.5 p*H* magnitudes.

The elongation of *R*^1^ in a structure of (**Ib**)–(**IVb**) (Figure 1) increased their values of the *MolVol* parameter that varied from 405.59 Å^3^ to 458.91 Å^3^ (Table 2).

The compounds (**1b**)–(**12b**) differed in values of *MW*, F*sp*^3^, *n*_rotb_, *TSA*, *MolFlex*, *MR* and *MolVol* descriptors due to differences in nature of both *R*^1^- and *R*^2^-substituents (Figure 1). All of these molecules had the same data describing their *n*_ON_ (7) and *n*_OHNH_ (2), as could be expected.

The position of an *F*-substituent in a structure of corresponding positional isomers containing an identical *R*^1^-chain (Figure 1) from the set (**1b**)–(**8b**) was not reflected in values of their F*sp*^3^, *n*_rotb_, *n*_ON_, *n*_OHNN_, *TSA*, *MR* and log *P* parameters, as would be expected (Table 1 and Table 2). The introduction of *R*^2^ = 2′-OCH_3_ (Figure 1) instead of *R*^2^ = 2′-F increased F*sp*^3^s, *n*_rotb_s, *MR*s and *MolVol*s.

The predicted values of a *tPSA* descriptor for these derivatives were the following: 91.34 Å^2^ for the molecules containing the 2′-/4′-*F*-substituent, and 100.57 Å^2^ for the compounds with the 2′-OCH_3_-substitution.

Taking into account particular predictive algorithms, the lipophilic nature of analyzed derivatives (**1b**)–(**12b**) decreased in rank as follows: CLOGP 4.0 > MolLogP > XLOGP3 > pkCSM > WLOGP > MLOGP (Table 2). Their log *P*_av_ data were 3.79 connected with (**4b**), 3.78 related to (**8b**), 3.47 calculated for (**12b**), 3.40 generated for both (**3b**) and (**7b**), 3.09 connected with (**11b**), 2.91 related to (**2b**), 2.90 calculated for (**6b**), 2.62 generated for (**10b**), 2.54 connected with (**1b**), 2.53 related to (**5b**) and, finally, 2.22 calculated for (**9b**). The presence of 2′-OCH_3_ decreased particular log *P* readouts compared to those of identical positional isomers containing a more lipophilic 2′-/4′-*F*-substituent (Table 2).

Objectively, despite the fact that the log *P* values were previously experimentally determined for the compounds (**1**)–(**12**) in an octan-1-ol/phosphate buffer (p*H* = 7.0) partition system [17,18], the validity of obtained experimental data might be rather questionable in this case. The reason for such a critical statement was insufficient solubility of these salts in an aqueous phase used in the chosen partition system which was reflected in relatively low (not accurate) log *P*s determined for some of them. The experimental log *P* data of the salts ranged from 2.31, as observed for (**10**), to 5.07, as determined for (**8**) [17,18]. Therefore, this critical view on estimation of the log *P* parameters prevented their correct comparison to theoretical log *P*_av_ values.

The differences in length of *R*^1^ did not affect p*K*_a_s of particular pairs of compounds containing the same *R*^2^-groups (Figure 1, Table 2). The electron-donating moiety, as the substituent *R*^2^, slightly increased the p*K*_a_ values compared to the effect of an electron-withdrawing atom. The differences between currently calculated p*K*_a_ for (**1b**)–(**12b**) and previously estimated data related to individual salts (p*K*_a_s ≥ 7.0; Table S2) [17,18] were very roughly up to 1.0 p*H* magnitudes.

The 4′-*F*-substitution provided slightly higher *MolVol*s compared to those of the 2′-*F*-substituted molecules. The introduction of 2′-OCH***_3_*** increased the values of this parameter as well (Table 2).

The anti-TB effective compounds (**13**) and (**14**) contained the lowest fraction of *sp*^3^-hybridized *C*-atoms (F*sp*^3^ = 0.29) and their chemical structure indicated the lowest flexibility from an entire analyzed set of molecules. This statement was supported by corresponding *n*_rotb_ = 9, as well as by the values of a *MolFlex* parameter of 0.4244 and 0.4574, respectively. Both derivatives were predicted to be the most lipophilic (Table 1), defined by log *P*_av_ of 4.90 and 4.89, respectively. The values of their calculated p*K*_a_ descriptors were identical to p*K*_a_ of the molecules belonging to the set (**9b**)–(**12b**). The predicted molecular volume of the compound (**13**), *MolVol* = 480.86 Å^3^, was slightly lower compared to that of the derivative (**12b**), characterizing by *MolVol* = 491.04 Å^3^ (Table 2).

### 3.2. In Silico Investigation of Non-Protonated Molecules (***Ib***)–(***IVb***), (***1b***)–(***12b***), (***13***) and (***14***)—Prediction of Some Pharmacokinetic Properties

Considering the evaluation of particular derivatives (**Ib**)–(**IVb**), their intestinal absorption predicted for humans was relatively high and was defined by IA ≥ 90.17% (Table 3). The increased *MW* and lipophilicity (log *P*_av_) slightly decreased the values of IA. However, absolutely favorable intestinal permeability was not predicted when a theoretical Caco-2 model was used (Table 3); the values of the log *P*_app_ descriptor ranged from 0.422, as calculated for (**IIIb**), to 0.652, as generated for (**Ib**).

Practically all of these non-protonated molecules were predicted to poorly cross the BBB by a passive mechanism. Strictly taken, the derivative (**Ib**) could be regarded as an exception because it was borderline with its log BB = −0.962 (Table 3). In fact, more precise in silico evaluation of the ability of compounds to enter the CNS was related to generated values of the log PS parameter. If log PS was <−3.000 then these derivatives were considered unable to enter the CNS. In regard to a given descriptor, the ability of the compounds (**Ib**)–(**IVb**) to passively permeate this biological system could be moderate to notably limited because their log PS values might be found at around −2.830 (Table 3).

When attention was paid to a group of phenylcarbonyloxypiperazinopropanols with *R*^2^ ≠ H (Figure 1), their intestinal absorption predicted for humans (Table 3) ranged from moderate to high. The calculated IA parameter was found to be in the interval from 70.51%, as calculated for (**9b**), to 91.73%, assigned to (**5b**). The introduction of a relatively hydrophilic 2′-OCH_3_-moiety (compared to lipophilicity of the *F*-atom) lowered the extent of passive intestinal absorption.

**Table 3:** The prediction of several parameters related to absorption (intestinal absorption and Caco-2 permeability in humans) and distribution (permeability through the BBB and CNS), as well as prediction of quantitative estimate of drug-likeness (*QED* parameter) of the compounds (**Ib**)–(**IVb**), (**1b**)–(**12b**), (**13**) and (**14**) through the pkCSM [11] and DruglikeFilter ver. 1.0 [32] interactive applets.

^1^ Cmpd.	^3^ IA (%)	^4^ log *P*_app_	^5^ log BB	^6^ log PS	^7^ *QED*
(**Ib**)	91.62	0.652	−0.962	−2.855	0.67
(**IIb**)	91.15	0.589	−1.010	−2.858	0.63
(**IIIb**)	90.49	0.422	−1.040	−2.833	0.58
(**IVb**)	90.17	0.452	−1.071	−2.821	0.42
(**1b**)	91.17	0.639	−1.184	−3.310	0.65
(**2b**)	90.71	0.576	−1.231	−3.293	0.60
(**3b**)	90.05	0.409	−1.262	−3.234	0.56
(**4b**)	89.73	0.439	−1.293	−3.188	0.40
(**5b**)	91.73	0.712	−1.187	−3.330	0.65
(**6b**)	91.27	0.649	−1.235	−3.313	0.60
(**7b**)	90.61	0.482	−1.266	−3.254	0.56
(**8b**)	90.29	0.512	−1.297	−3.208	0.40
(**9b**)	70.51	0.702	−1.176	−3.379	0.60
(**10b**)	71.27	0.638	−1.223	−3.373	0.55
(**11b**)	72.19	0.472	−1.254	−3.316	0.51
(**12b**)	73.09	0.501	−1.285	−3.269	0.37
^2^ (**13**)	96.23	1.665	0.013	−0.708	0.36
^2^ (**14**)	93.89	0.799	0.256	−1.116	0.36

^1^ Cmpd. = compound, ^2^ (**13**), (**14**) = both compounds were synthesized and screened in vitro in [7], ^3^ IA (%) = intestinal absorption in humans (in percentages), ^4^ log *P*_app_ = decadic logarithm of an apparent permeability coefficient (*P*_app_), ^5^ log BB = logarithmic value of blood-to-brain ratio [11], ^6^ log PS = blood–brain barrier permeability–surface area product [11], ^7^ *QED* = quantitative estimate of drug-likeness [32].

Favorable intestinal permeability was not predicted for the compounds (**1b**)–(**12b**) when employing the Caco-2 model (Table 3). The values of their log *P*_app_ parameter ranged from 0.409, as calculated for (**3b**), to 0.712, as generated for (**5b**). The chosen model suggested moderate permeability because the log *P*_app_ data related to all investigated non-protonated derivatives were in the interval of 0.000–0.900 [11]. The 2′-*F*-substitution seemed to be the most convenient in order to increase the log *P*_app_ outputs (Table 3).

All derivatives (**1b**)–(**12b**) were predicted to cross the BBB very poorly because their log PS ≤ −3.188 (Table 3). In fact, their passive permeation through that physiological barrier was theoretically impossible.

The extent of passive intestinal absorption predicted for both (**13**) and (**14**) was slightly higher (Table 3) compared to intestinal absorption of (**1b**)–(**12b**). The naphthalene–NAP hybrid derivatives (Figure 2) provided greater in silico permeability considering the Caco-2 predictive model as indicated by their log *P*_app_ ≥ 0.799. In fact, the ability of the compounds (**13**) and (**14**) to passively cross the BBB (log BB ≥ 0.013), as well as ability to enter the CNS (log PS ≥ −1.116), was notably higher than for the derivatives (**Ib**)–(**IVb**) and (**1b**)–(**12b**).

The web-based GLORYx applet [30] was used to predict the existence of individual metabolites of evaluated molecules (**Ib**)–(**IVb**) and (**1b**)–(**12b**). Their free OH-group (Figure 1) was indicated as a suitable moiety to be *O*-glucuronidated within phase II of biotransformation. The priority score value, theoretically found within the interval of 0.00–1.00, for this phase II metabolic process was practically identical for all investigated derivatives and ranged between 0.59 and 0.60 (Table S4).

A slightly less probable possibility could be the oxidative opening of a heterocyclic piperazin-1,4-diyl portion within a structure of (**Ib**)–(**IVb**) and (**1b**)–(**12b**), *N*^4^-oxidation of this cycle or oxidative hydroxylation at proper positions of their distal phenyl ring (priority score ≤ 0.51 in all cases; Table S4). The calculated values of a priority score index also showed that a carbamate (NHCOO) moiety in the structure of these derivatives could be less susceptible to hydrolysis (priority score ≤ 0.30 for all inspected molecules) than an ester fragment (priority score ≤ 0.50) as its part. All these reactions would be involved in phase I metabolic processes.

Regarding the derivatives (**9b**)–(**12b**), *O*-demethylation, as a reaction belonging to phase I biotransformation, should be taken into consideration (priority score ≤ 0.36; Table S4). The elongation of the *R*^1^-substituent (Figure 1) slightly decreased the probability of such biochemical modification (priority score ≤ 0.30 for all investigated compounds).

Finally, *O*-sulfation of the OH-group in the structure of (**Ib**)–(**IVb**) and (**1b**)–(**12b**), as a phase II biotransformation reaction, would be less probable (priority score ≤ 0.39 for all these derivatives) compared to its *O*-glucuronidation (Table S4).

Overall, the calculated *QED* descriptor (Table 2) of the derivatives (**Ib**)–(**14**) ranged from 0.36, as generated for both (**13**) and (**14**), to 0.67, as calculated for (**Ib**). Therefore, the improvement in structural and physicochemical properties of all these molecules could be achieved via very precise modifications of their structure, taking into account the maintenance of the desired pharmacological activity or even its improvement.

### 3.3. Relationships Between Structural, Physicochemical and Pharmacokinetic Parameters of Non-Protonated Molecules (***Ib***)–(***IVb***) and (***1b***)–(***12b***)

The PCA technique [34,35] was employed to evaluate the relationships between the values of *MW*, F*sp*^3^, *TPSA*, *MolFlex*, *MR* (Table 1), *MolVol* (Table 2), log *P*_av_, IA, log *P*_app_, log BB, log PS and *QED* (Table 3) parameters calculated for the derivatives (**Ib**)–(**IVb**) and (**1b**)–(**12b**). In fact, the values of given descriptors related to the compounds (**13**) and (**14**) were not included in this PCA. The reason was that the majority of these outputs were identical for a given pair of derivatives (Table 1, Table 2 and Table 3). Thus, if involved, the relevance of such an analysis would be reduced.

The PC 1, PC 2 and PC 3 readouts accounted for 93.01% of the total variance and their contributions were the following: PC 1 (62.19%, *λ*_e_ = 7.46), PC 2 (20.48%, *λ*_e_ = 2.49) and PC 3 (10.34%, *λ*_e_ = 1.24). The relationship between PC 1 and PC 2 (accounting for total variance of 82.67%) allowed for the distribution of investigated non-protonated compounds into several subcategories.

A positive feature of the present analysis was that all involved parameters were projected onto a 2D coordinate system based on a selection of PC 1 and PC 2 (PC 1 × PC 2). One group of active observations that was defined by PC 1 < 1.000 (can be found on the left side within Figure 3a) consisted of the compounds containing the 4-phenylpiperazin-1-yl moiety, excluding the derivative (**IVb**) (Figure 1), as well as molecules with *R*^1^ = CH_3_ or C_2_H_5_, and *R*^2^ ≠ H (Figure 1). They were characterized by the lowest values of *MW* ≤ 445.48 g/mol, *TPSA* ≤ 348.27 Å^2^, *MR* ≤ 129.68 m^3^/mol and *MolVol* ≤ 441.00 Å^3^. On the other hand, they would be absorbed to the greatest extent (IA ≥ 90.49% excluding the compound (**9b**)) and most of them were described by relatively higher values of log *P*_app_ (excluding the derivative (**IIIb**)). In addition, these derivatives showed relatively high *QED* ≥ 0.58.

Slightly higher values of *MW* (in the range of 455.55–459.51 g/mol), *TPSA* (354.62–362.03 Å^2^), *MR* (129.64–134.49 m^3^/mol) and *MolVol* (446.83–458.91 Å^3^) could be observed for the compounds (**IVb**), (**3b**), (**7b**) and (**10b**) found in the middle of this 2D biplot. A more positive PC 2 indicated their higher lipophilicity described by corresponding log *P*_av_s.

Another group of active observations represented by the derivatives (**4b**), (**8b**), (**11b**) and (**12b**) was defined by relatively high *MW*, *TPSA* and *MR*. The increased value of *MolVol* moved these compounds to the right side of Figure 3a. These molecules were characterized by log *P*_app_ ≤ 0.512 (*P*_app_ ≤ 3.25 × 10^−6^ cm/s), indicating moderate intestinal permeability [11]. Moreover, their *QED*s ≤ 0.51 showed rather notable room for optimizing their properties compared to those of approved drugs.

The highest values of *MW*, F*sp*^3^, *TPSA*, *MR* as well as *MolVol* were related to the compound (**12b**) that was also defined by a relatively low value of the *QED* parameter. This molecule was characterized by the highest PC 1 (PC 1 = 4.920, PC 2 = −1.045). Thus, PC 1 could be considered a rather suitable factor to distinguish between the compounds showing the highest values of chosen structural and physicochemical parameters, and the molecules defined by relatively high *QED*s.

The distance between individual descriptors (variables) on a plane and constructed circle of correlation (Figure 3b) expressed the quality of their 2D representation on a particular PC. This cycle was defined by a radius of 1.000 (absolute value) and quality of the variables’ presentations was defined by square cosine (cos^2^) parameters expressing the proportion of a variable’s variance that was accounted for by the individual PC [34,35]. The cos^2^ descriptors connected with the most optimally presented variables were the following: 0.989 (*TPSA*; this value was related to PC 1), 0.964 (*MolVol*; PC 1), 0.954 (*MW*; PC 1), 0.952 (F*sp*^3^; PC 1) and 0.950 (*MR*; PC 1). Other variables related to structural and physicochemical parameters were characterized by cos^2^ of 0.713 (log *P*_av_; PC 1) and 0.686 (*MolFlex*; PC 2), respectively. In fact, individual pharmacokinetic parameters provided less convenient cos^2^ data of 0.516 (log *P*_app_; PC 1), 0.495 (log PS; PC 2), 0.454 (IA; PC 2) and 0.388 (log BB; PC 1). The *QED* variable was defined by a satisfactory cos^2^ value of 0.872 (PC 1).

Relatively close positive correlations were observed between particular pairs of descriptors from a set, consisting of *MW*, F*sp*^3^, *TPSA*, *MR* and *MolVol*, as visualized in Figure 3b. These relationships were characterized by particular *r* values of ≥0.912 (Table S5). The log *P*_av_ parameter most positively correlated with both F*sp*^3^ (*r* = 0.825) and *MW* (*r* = 0.800). Interestingly, the strongest negative correlation was calculated between a given lipophilicity descriptor and *QED* (*r* = −0.842), as well as between log *P*_av_ and log *P*_app_ (*r* = −0.841).

### 3.4. In Vitro Screening of the Compounds (***I***)–(***IV***) Against Particular Mycobaterial Strains

The majority of currently in vitro screened derivatives were defined by relatively high values of the *MIC* parameter, excluding 1-(3-{4-[(butoxycarbonyl)amino]benzoyloxy}-2-hydroxypropyl)-4-phenylpiperazin-1-ium chloride (**IV**). This compound showed *MIC* = 62.5 μM against *Mtb* H_37_R_v_ (both 14-d and 21-d incubation), *MK* My 235/80 (7-d incubation) as well as MK_ci_ 6509/96 (7-d incubation). The INH and AMK standard drugs, however, inhibited growth of relevant (susceptible) mycobacterial strains at lower *c*s (Table 4).

**Table 4:** The in vitro activity expressed as the minimum inhibitory concentration values (*MIC*; in μM units) of the compounds (**I**)–(**IV**), as well as isoniazid (INH) and amikacin (AMK) reference drugs against *M. tuberculosis* H_37_R_v_ (*Mtb* H_37_R_v_), *M. kansasii* My 235/80 (*MK* My 235/80), *M. kansasii* 6509/96 clinical isolate (*MK*_ci_ 6509/96) and *M. avium* My 330/88 (*MA* My 330/88), respectively.

^1^ Cmpd.	*MIC* (μM)									
	*Mtb* H_37_R_v_		*MK* My 235/80			*MK*_ci_ 6509/96			*MA* My 330/88	
	^2^ 14 d	^3^ 21 d	^4^ 7 d	14 d	21 d	7 d	14 d	21 d	14 d	21 d
(**I**)	250	500	500	500	500	500	500	500	500	500
(**II**)	125	250	250	250	250	250	250	250	250	250
(**III**)	125	250	125	250	250	125	250	250	250	250
(**IV**)	62.5	62.5	62.5	125	250	62.5	125	250	125	250
INH	0.5	1	>250	>250	>250	2	4	8	>250	>250
AMK	*nd*	*nd*	0.5	1	1	0.5	0.5	0.5	1	2

^1^ Cmpd. = compound, ^2^ 14 d = 14-day cultivation, ^3^ 21 d = 21-day cultivation, ^4^ 7 d = 7-day cultivation. *nd* = not determined (compound was not used as a reference drug in particular in vitro evaluations).

The introduction of *R*^2^ ≠ H at the aromatic ring within the 4-(*R*^2^-substituted phenyl)piperazin-1-yl moiety together with the presence of a non-branched 4-NHCOOC_4_H_9_-chain attached to a phenylcarbonyloxy fragment (Figure 1) maintained or even improved in vitro efficacy (*MIC* = 16–62.5 μM) of such designed compounds [6] against the abovementioned mycobacterial species, showing *MIC* = 62.5 μM and 32 μM, respectively (Table S1). Moreover, the 2′-/4′-*F-*substitution improved in vitro activity against *MA* My 330/88 [6] compared to efficacy of the derivatives (**I**)–(**IV**) or the compounds with 2′-OCH_3_.

In addition, the derivatives (**I**)–(**IV**) and (**1**)–(**12**) showed some more favorable organoleptic and physicochemical characteristics compared to their non-protonated derivatives (**Ib**)–(**IVb**) and (**1b**)–(**12b**), being synthesized as oily yellow-orange-colored compounds (Figure 1). For example, the processing with biologically screened solid salts was more effective in particular in vitro experiments, and, in addition, the substances (**I**)–(**IV**) and (**1**)–(**12**) were more soluble in various aqueous media than their non-protonated forms. The favorable consequence was that the precipitation of evaluated samples (individual salts) was not observed in particular experimental steps of the performed biological screening.

### 3.5. In Silico Investigation of Non-Protonated Molecules (***Ib***)–(***IVb***) and (***1b***)–(***12b***), as Well as Their Salts (***I***)–(***IV***) and (***1***)–(***12***)—Molecular Docking

The LigPrep tool [46] was employed to prepare two series of non-protonated ligands (**Ib**)–(**IVb**) and (**1b**)–(**12b**), as well as two sets of corresponding ligand salts (**I**)–(**IV**) and (**1**)–(**12**) for MD procedures. All these compounds were analyzed in relevant conformations according to their 3D arrangement, considering also the 3D orientation of particular substituents attached to a stereogenic center present in their structure. Thus, they were divided into individual (*R*)- and (*S*)-enantiomers (Tables S6–S11).

The Prime MM-GBSA method [51] was employed for evaluating the binding energies of individual non-protonated derivatives and salts toward a proposed target protein, ATP synthase. The calculated MM-GBSA energies, that is, binding free energies described by the MM-GBSA ΔG Bind parameter (Tables S6 and S7), were used as a criterion to choose the best ligand conformers.

The binding energies of non-protonated compounds varied from −61.96 kcal/mol, as calculated for the (*S*)-enantiomer of the compound (**9b**), to −36.37 kcal/mol, generated for (***S***)-(**IIb**). When attention was specifically paid to the series (**Ib**)–(**IVb**), individual (*R*)-enantiomers provided higher affinity for ATP synthase than corresponding (*S*)-enantiomers, excluding an enantiomeric pair related to the compound (**IVb**). The highest affinity (MM-GBSA ΔG Bind = −56.43 kcal/mol) was calculated for (***R***)-(**IIIb**). The elongation of *R*^1^ (Figure 1) favored (*R*)-enatiomers of particular non-protonated ligands containing *R*^2^ = 4′-F, 2′-F or 2′-OCH_3_ as the molecules with higher affinity for ATP synthase than those with relatively shorter *R*^1^ (Table S6).

In general, the enantiomeric forms of individual salts showed higher affinity for the desired pharmacological target than the (*R*)- or (*S*)-enantiomers of corresponding non-protonated derivatives (Tables S6 and S7). The values of MM-GBSA ΔG Bind for individual enatiomers of (**I**)–(**IV**) and (**1**)–(**12**) were in the interval from −70.09 kcal/mol, as calculated for (***R***)-(**7**), to −42.28 kcal/mol, as generated for (***S***)-(**9**). Furthermore, the (*R*)-enantiomers of almost all presently analyzed salts showed higher affinity for ATP synthase compared to that of the (*S*)-ones (Table S7), excluding enantiomeric pairs of the compounds (**2**), (**6**), (**8**) and (**11**).

The calculated docking score values (Tables S8 and S9) confirmed these trends in regard to the interactions of investigated non-protonated compounds and salts with a suggested pharmacological target. The outputs generated for particular salt ligands were more negative than those of corresponding derivatives from both sets (**Ib**)–(**IVb**) and (**1b**)–(**12b**). The lowest docking score (−6.306 kcal/mol) was related to the compound (***R***)-(**II**), the highest value of this decriptor (−4.377 kcal/mol) was generated for the molecule (***S***)-(**9**). The type of interactions between particular substituents attached to position 2′ and/or 4′ of a distal phenyl ring of the compounds (**I**)–(**IV**) and (**1**)–(**12**), and individual interacting AAs present in an active site (binding pocket) of ATP synthase, can be found in Table S10.

Further discussion might be specifically oriented to the most effective antimycobacterial agent from the set (**I**)–(**IV**), as well as to the most active molecule from the series (**1**)–(**12**) [6]. Notable differences between individual enantiomers of (**IVb**) and (**IV**) were observed regarding particular values of MM-GBSA ΔG Bind (Tables S6 and S7). The compound (***R***)-(**IV**) provided the highest affinity for ATP synthase (MM-GBSA ΔG Bind = −67.04 kcal/mol; Table S7). The *N*-atom of its NHCOO-moiety formed a hydrogen bond with a proline (Pro 2:43) AA residue (Figure 4).

The formation of other hydrogen bonds was also predicted, involving the OH-group of a connecting 2-hydroxypropan-1,3-diyl chain of this ligand (its OH interacted with a serine (Ser *a*:77) AA residue) and a protonated *N*-atom (this atom interacted with a carboxylate moiety of an alanine (Ala *a*:78) AA residue) incorporated in its cyclic piperazin-1,4-diyl system. Furthermore, the *C*-atom of the NHCOO-fragment formed a hydrophobic interaction with a valine (Val 2:39) AA residue (Figure 4).

The *a* letter in the labeling of particular AA residues indicated the *a* subunit in the structure of ATP synthase, and the number 2 in the labeling of particular AA residues indicated an α-helix number 2 within the *c* subunit of this mycobacterial enzyme.

The mode in which the derivative (***R***)-(**IV**) was docked into an active site of ATP synthase in *Mtb* H_37_R_v_ is shown in Figure S1 (Section C; Supplementary Materials).

As in the case of (**IV**), notable differences between individual enantiomers of (**12b**) and (**12**) were observed regarding particular values of MM-GBSA ΔG Bind (Tables S6 and S7). The compound (***R***)-(**12**) showed the highest affinity for ATP synthase (MM-GBSA ΔG Bind = −68.90 kcal/mol). The *N*-atom of its NHCOO-moiety formed a hydrogen bond with the Pro 2:43 AA residue (Figure 5).

The formation of other hydrogen bonds was also predicted, involving the OH-group of a connecting 2-hydroxypropan-1,3-diyl chain (its OH interacted with the Ser *a*:77 AA residue), *O*-atom (this atom interacted with the Ser *a*:77 AA residue as well) of a CO group as a part of a polar NHCOO-chain, and a protonated *N*-atom (this atom interacted with a carboxylate moiety of the Ala *a*:78 AA residue) incorporated in its cyclic piperazin-1,4-diyl system (Table S11). Furthermore, the *C*-atom within the NHCOO-fragment was predicted to form hydrophobic interactions with the Val 2:39 AA residue (Figure 5).

The mode in which the compound (***R***)-(**12**) was docked into an active site of ATP synthase in *Mtb* H_37_R_v_ is shown in Figure S2 (Supplementary Materials).

The reason why individual salts interacted more effectively with ATP synthase of *Mtb* H_37_R_v_ compared to the interactions involving corresponding non-protonated derivatives was that a so-called salt bridge was formed between the salts’ *N*-atom (as being integrated in the piperazin-1,4-diyl heterocycle) and a negatively charged counterpart of the Ala *a*:78 AA residue. In fact, that type of very beneficial non-covalent interaction was not observed for particular non-protonated molecules.

### 3.6. In Vitro Antiproliferative Properties of Chosen Compounds

Considering the results of current and previous [6] in vitro antimycobacterial screenings, the compounds (**IV**) and (**12**), as the most efficient anti-*Mtb* H_37_R_v_ agents from both series (**I**)–(**IV**) and (**1**)–(**12**), as well as INH, were chosen to investigate their in vitro antiproliferative and eventual cytotoxic properties. In normal human dermal fibroblasts, none of these compounds decreased viability below 100.0% at *c*s up to 62.5 µM (Figures S3–S5; Supplementary Materials).

The derivative (**12**) and INH reduced viability up to 70.0% across the entire tested concentration range that was consistent with the definition of in vitro non-cytotoxicity published by the International Organization for Standardization and evidenced in ISO 10993-5 [61]. In this regard, only the compound (**IV**) showed borderline behavior regarding its impact on cell viability at the highest concentration used, *c* = 250 µM (68.4 ± 2.0% cell viability relative to control cells). As shown by microscopic figures (Figures S6–S8; Supplementary Materials), the antiproliferative effect of (**IV**) might account for observed experimental outcome rather than a reduction in cell viability (Figure S7).

### 3.7. In Silico Investigation of Non-Protonated Molecules (***Ib***)–(***IVb***) and (***1b***)–(***12b***)—Inspection of Their Possibilities to Inhibit Growth of Particular Mycobacteria

The AntiBac-Pred applet [57] integrated in the PASS Online Predictor tool [58] was used to evaluate the compounds (**Ib**)–(**IVb**) and (**1b**)–(**12b**) in silico as the agents that could theoretically effectively combat some other mycobacterial strains. The generated outputs indicated that these derivatives could effectively act especially against *M. mageritense*, avirulent *Mtb* H_37_R_a_ and *M. bovis* BCG, a live-attenuated strain of *M. bovis*. However, particular values of the score index did not exceed 0.3000 (Table S12). The derivative (**Ib**) was evaluated as the most promising against *M. mageritense* (score = 0.2731), the molecule (**4b**) showed the most prospective theoretical potential to inhibit growth of *Mtb* H_37_R_a_ (score = 0.2484) and compound (**IIb**) was characterized by the highest value of score (0.1814) that predicted its chance of being effective against *M. bovis* BCG.

In fact, the predictor [57] also provided other mycobacterial strains, namely, resistant *Mtb* H_37_R_v_, resistant *M. ulcerans*, resistant *M. avium* or *M. phlei*, which could be potentially affected by the evaluated non-protonated derivatives. However, corresponding values of the score index did not exceed 0.1000 in most cases or these data were roughly around this arbitrary threshold value (Table S12).

## 4. Discussion

### 4.1. In Silico Investigation of Non-Protonated Molecules (***Ib***)–(***IVb***), (***1b***)–(***12b***), (***13***) and (***14***)—Prediction of Some Structural and Physicochemical Properties

The calculated values of individual structural and physicochemical descriptors for particular derivatives would not only be discussed in terms of ‘simple comparison’ to data that characterized antimycobacterial drugs but also in the context of possibilities to affect some pharmacokinetic characteristics of these molecules, especially their capability of being passively absorbed after administration per os or to passively permeate through the BBB. The *MW* values of individual non-protonated (substituted) phenylcarbonyloxypiperazinopropanol-based compounds, as well as the derivatives (**13**) and (**14**), were <500.00 g/mol (Da; Table 1) and that observation was consistent with a previous analysis aiming at properties of anti-TB drugs [62]. This research concluded that approximately half of synthetic small-molecule therapeutics belonging to a given pharmacodynamic group were characterized by *MW* = 300.00–500.00 g/mol (Da). One of the exceptions, bedaquiline (BDQ; **17**), chemically (1*R*,2*S*)-1-(6-bromo-2-methoxyquinolin-3-yl)-4-(dimethylamino)-2-(naphthalen-1-yl)-1-phenylbutan-2-ol, with *MW* = 555.55 g/mol (Da) will be discussed in the next sections of this paper. The chemical structure of the compound (**17**), also known by its TMC207 and R207910 code names, is provided in Figure 6.

The values of *n*_ON_ calculated for all investigated derivatives ranged between 4 and 7 (Table 1) and such a variability was also observed in a group of clinically used anti-TB agents. Similarly, the *n*_OHNH_ parameters for all presently analyzed non-protonated arylcarbonyloxypiperazinopropanols, and the molecules (**13**) and (**14**) were comparable (Table 1) to *n*_ON_s of anti-TB drugs. When the *n*_OHNH_ data were taken into consideration, a relatively lower count of such moieties could be found in the structure of anti-TB agents. For example, BDQ (**17**) was characterized by both *n*_ON_ = 4 and *n*_OHNH_ = 1 [62]. A relatively high *n*_rotb_ parameter, as observed for the derivatives presently investigated in silico, was also connected with anti-TB molecules approved by official regulatory authorities worldwide, including BDQ (**17**) defined by *n*_rotb_ = 8 [62].

Approximately half of approved anti-TB drugs had values of the calculated log *P* (clog *P*) parameter in the interval of −1.00 < *c*log *P* < 3.00 [62]. The non-protonated forms of the most effective antimycobacterial compounds from both sets (**I**)–(**IV**) and (**1**)–(**12**) were characterized as relatively highly lipophilic. The molecules (**IVb**) and (**12b**) were defined by log *P*_av_ of 3.51 and 3.47, respectively, and their lipophilicity could be comparable to that of pretomanid (*c*log *P* = 3.63), a third-line anti-TB drug belonging to a nitroimidazooxazine class. Lipophilicity of BDQ (**17**) was described by an even higher value of *c*log *P* = 7.13 [62].

### 4.2. In Silico Investigation of Non-Protonated Molecules (***Ib***)–(***IVb***), (***1b***)–(***12b***), (***13***) and (***14***)—Prediction of Some Pharmacokinetic Properties

The biologically active compounds could be passively absorbed if they met several structural and physicochemical criteria defined by values of particular parameters as follows [27,63,64]: *MW* ≤ 500.00 g/mol (Da), *n*_ON_ ≤ 10, *n*_OHNH_ ≤ 5, CLOGP 4.0 ≤ 5.00 (or MLOGP ≤ 4.15) and *tPSA* ≤ 140.0 Å^2^, respectively. Varma et al. (2010) [65] defined the optimal bioavailability of pharmacologically effective molecules after being administered per os with threshold values of *MW* < 500.00 g/mol (Da), sum of (*n*_ON_ + *n*_OHNH_) ≤ 9 and *n*_rotb_ ≤ 12. In addition, relatively lower flexibility (*MolFlex* parameter) could contribute to better oral bioavailability of drugs [66].

The mean p*H* value of gastric juice is 1.92 ± 1.28 in men, 2.59 ± 2.08 in women and 3.27 in children, respectively. A p*H* value varying from 4.00 to 7.00 can be found in the small intestine [67]. Therefore, passive absorption of analyzed non-protonated compounds with theoretical p*K*_a_ readouts of 6.1–6.9 (Table 2) would be very limited from the gut. A more convenient situation would be if estimated p*K*_a_ values of (**I**)–(**IV**) ranging from 4.6 to 5.0 (Table S2) [16] might be taken into consideration. On the other hand, a completely different extent of absorption could be expected for the environment of the small intestine. A lower extent of passive absorption might be predicted within particular compartments of the small intestine with lower p*H*. The increased p*H* would support the increased absorption of evaluated compounds.

Considering the derivatives (**1**)–(**12**) defined by experimental p*K*_a_s ≥ 7.0 (Table S2) [17,18], it should be expected that their passive absorption will be limited. In fact, the increasing p*H* gradient along the gastrointestinal tract (GIT) enhanced their permeability.

Ren and Lien (2000) [68] concluded that eventual connections between passive absorption of pharmacologically active compounds from the GIT to the bloodstream and results of the Caco-2 cell permeability evaluations should be interpreted with caution. This finding was related to the derivatives defined by log *P*_app_ < 5.00 × 10^−6^ cm/s. The current calculations provided moderate to very satisfactory absorption of all analyzed molecules (Table 3). In a series consisting of non-protonated (substituted) phenylcarbonyloxypiperazinopropanols, the log *P*_app_ varied from 2.56 × 10^−6^ cm/s, as predicted for (**3b**), to 5.15 × 10^−6^ cm/s, connected with (**5b**).

The compounds (**13**) and (**14**) were characterized by log *P*_app_ of 4.62 × 10^−5^ cm/s and 6.30 × 10^−6^ cm/s, respectively. Thus, their higher extent of absorption could be assumed. That prediction was reflected in the highest percentage values of intestinal absorption for both molecules (Table 3).

The intestinal absorption of BDQ (**17**) predicted through pkCSM [11] was 94.38%, its log *P*_app_ was of 1.132 (*P*_app_ = 1.36 × 10^−5^ cm/s) according to the same predictor tool. On the other hand, high lipophilicity of BDQ (**17**) set ‘a certain level of uncertainty’ in regard to such predicted high passive absorption of this drug via the pkCSM interactive engine [11]. In fact, poor solubility and insufficient intestinal permeability of BDQ (**17**) were two primary reasons why researchers prepared specific delivery systems—micelles loaded with a given drug—and expected its better solubility, more effective cell permeation via intestinal wall and increased cellular uptake as well. Their assumptions and expectations were fulfilled as reported in [66].

Lipophilicity and sum (number; *n*) of polar atoms, that is, oxygens (*n*_O_) and nitrogens (*n*_N_), incorporated in a structure of molecules affected their ability to passively enter the CNS. Simple relationships of (*n*_N_ + *n*_O_) ≤ 5 and [CLOGP 4.0 − (*n*_O_ + *n*_N_)] > 0.00 indicated a high probability that compounds enter the CNS [69]. In addition, *tPSA* < 60.0–70.0 Å^2^ would also be a suitable indicator to predict whether a molecule might passively cross via the BBB to the CNS [70].

Broccatelli and his research team [71] concluded that, if compounds were characterized by p*K*_a_ < 5.5, their passive permeation through the BBB would be less probable.

Following *n*_N_ and *n*_O_ in the chemical structure of (**Ib**)–(**IVb**) and (**1b**)–(**12b**), as well as values of their *tPSA*, CLOGP 4.0, log BB and log PS parameters (Table 2 and Table 3), it might be predicted that these compounds would not be able to passively enter the CNS. This suggestion was also supported by outputs from the SwissADME applet [72]. An exception was the derivative (**Ib**) with ‘slightly higher virtual’ passive BBB permeability characterized by log BB = −0.962 (Table 3). Therefore, such an observation favored corresponding salts to theoretically be used as antimycobacterial agents in cases where their permeation through the BBB would not be required. This finding could be important especially for the compounds (**1**)–(**12**) described by experimental p*K*_a_ values of ≥7.0 (Table S2) [17,18].

In fact, such a theoretical proposal based on *tPSA*, CLOGP 4.0, log BB and log PS suffered from several limitations. These predictions did not consider some other factors, namely, transporter effects (for example, P-glycoprotein efflux), ionization state or peripheral biotransformation of compounds [73,74,75].

The data listed in Table 2 related to the derivatives (**13**) and (**14**), as well as parameters connected with a highly efficient anti-TB BDQ (**17**) agent, which was characterized by *n*_ON_ = 4, *n*_OHNH_ = 1, *TSA* = 393.23 Å^2^, *tPSA* = 45.59 Å^2^ and *MolFlex* = 0.3683, indicated that all these molecules might be able to passively permeate via the BBB to the CNS. This prediction was supported by generated values of both log BB and log PS parameters (Table 3).

Relatively higher ratios of *TSA* and *n*_OHNH_ [76], as well as currently generated values of log BB = 0.028 and log PS = −0.977, for BDQ (**17**) favored a possibility that this drug would be able to passively enter the CNS. This in silico finding was in agreement with the conclusions [77] about BDQ (**17**) as a synthetic small-molecule agent that was suitable to target TB reservoirs in the CNS.

Molecular volume and *MW* are considered appropriate descriptors to define the size of (not only) antimycobacterial compounds and their transport characteristics [78]. The calculated values of *MolVol* related to particular non-protonated derivatives (Table 2) might be successfully employed within the process of design and development of antimycobacterial agents to accurately predict their topological features [75,79].

Several authors defined specific structural and physicochemical characteristics to be met for compounds to show convenient drug-like properties. In that context, Ghose et al. [80] considered the molecules described by 160.00 Da < *MW* (in Da) < 480.00 Da, −0.4 < log *P* < 5.6, number of atoms < 70, and 40 m^3^/mol < *MR* (in m^3^/mol) < 130 m^3^/mol as those showing a suitable drug-like profile. Oprea [81] defined similar threshold values as follows: *n*_OHNH_ < 2, 2 < *n*_ON_ < 10, 2 < *n*_rotb_ < 8, and 1 < number of rings < 4.

Biotransformation of antimycobacterially active compounds (drugs) could more or less affect their pharmacodynamic and toxicological properties [82,83]. In humans, BDQ (**17**) was reported to be mainly metabolized within phase I biotransformation in the liver to monodesmethylbedaquiline. This metabolite was observed to be several times less effective against *Mtb* H_37_R_v_ than the parent compound. Further oxidative desmethylation provided a didesmethyl metabolite with de facto minimum ability to combat this slow-growing pathogen. Moreover, toxicological features of these products of biotransformation were less convenient compared to the characteristics observed for (**17**) [83]. Interestingly, only phase I biotransformation of BDQ (**17**) has been reported in humans [83].

It might be hypothesized that the most probable biotransformation pathway predicted for all investigated derivatives (**Ib**)–(**IVb**) and (**1b**)–(**12b**), and (indirectly) for the salts (**I**)–(**IV**) and (**1**)–(**12**), that is, their *O*-glucuronidation, would decrease anti-TB activity. This assumption was based on the outputs from current MD analyses; a free and properly oriented OH-group was considered one of the key structural features for retaining this type of pharmacological activity. Similarly, hydrolytic processes which these non-protonated forms and their salts could undergo, or oxidative desmethylation of the compounds (**9b**)–(**12b**) and (**9**)–(**12**) (Table S12), would cause the ineffectiveness of resulting metabolites against *Mtb* H_37_R_v_ as well. However, such a hypothesis should be taken with caution and relevant experiments (at least) under in vitro conditions have to be carried out to provide more comprehensive conclusions.

The *QED* parameter of analyzed derivatives (**Ib**)–(**14**) ranged from 0.36 to 0.67 (Table 3). Paradoxically, the highest *QED* score was connected with (**Ib**) as the least effective antimycobacterial agent. Surprisingly, the value of a given descriptor calculated for BDQ (**17**) was only 0.22. In regard to generated data (Table 1, Table 2 and Table 3), it might be preliminarily stated that there was room to structurally optimize and improve physicochemical as well as pharmacokinetic properties of all presently evaluated compounds including (**13**), (**14**) and even BDQ (**17**).

### 4.3. Relationships Between Structural, Physicochemical and Pharmacokinetic Parameters of Non-Protonated Molecules (***Ib***)–(***IVb***) and (***1b***)–(***12b***)

The increase in lipophilicity of non-protonated derivatives (**Ib**)–(**IVb**) and (**1b**)–(**12b**) negatively correlated with their predicted intestinal permeability when considering log *P*_app_. This relationship was characterized by *r* = −0.841 (Table S5). The increased *MW* values of these compounds more or less decreased their ability to permeate the BBB to the CNS. The relationship of *MW* versus log BB was defined by *r* = −0.771, and *MW* versus log PS was described by *r* = −0.227 (Table S5). This observation coincided with the fact that BBB limits the passive permeation of sterically bulky compounds due to tight junctions formed by lateral transmembrane proteins, lack of fenestrations, negative surface polarity and high occurrence of particular efflux transporters [84].

The increased fraction of *sp*^3^-hybridized *C*-atoms in a structure of analyzed non-protonated molecules as well as increased *TPSA* and *MolVol* moderately negatively contributed to their ability to passively cross the BBB, as particular *r* values of −0.457, −0.593 and −0.465 indicated (Table S5). This observation met conclusions published in [85,86,87].

A positive correlation was observed between log BB, describing the extent of brain penetration, and log PS that defined the rate of brain penetration for the investigated molecules (*r* = 0.666; Table S5). The log PS parameter could be regarded as a more informative index than log BB, especially in relation to the susceptibility of pharmacologically effective compounds (drugs) to active transport systems [88,89].

Optimization of the *MW*, F*sp*^3^, *TPSA* and *MR* parameters of (**Ib**)–(**IVb**) and (**1b**)–(**12b**) would be appreciated in order to increase their overall quantitative estimate of drug-likeness defined by the *QED* parameter. That observation is visualized in Figure 3b, as well as numerically expressed by particular *r* values (Table S5). The relationships of *MW* versus *QED*, F*sp*^3^ versus *QED*, *TPSA* versus *QED*, *MR* versus *QED*, and *MolVol* versus *QED* were defined by corresponding *r* outputs of −0.889, −0.910, −0.926, −0.919 and −0.909.

In addition, lipophilic properties of these non-protonated derivatives were not optimal in relation to *QED* (Table S5) since the relationship between log *P*_av_ and *QED* was defined by *r* = −0.842. If the presently analyzed set of compounds would be more robust then a quasiparabolic dependence between lipophilicity and *QED* could be assumed [31,90].

The current observations are also visualized in Figure 3b and could be interpreted following directions of particular lines related to individual variables. Each pair of these lines representing *MW*, F*sp*^3^, *TPSA*, *MR* and *MolVol* formed relatively small (sharp) angles. This behavior meant that given descriptors correlated each other most positively as proved by corresponding *r* values (Table S5). Conversely, the largest angles were observed between the lines representing *MW*, F*sp*^3^, *TPSA*, *MR*, as well as *MolVol*, and a line related to log *P*_app_. All of these relationships were described by negative *r* values (Table S5). In addition, the extent of predicted intestinal absorption was relatively less dependent on *MW*, F*sp*^3^, *TPSA*, *MR* or log *P*_av_ (Figure 3b).

Considering a group of pharmacokinetic descriptors only, that is, IA, log *P*_app_, log BB and log PS, the increase in log *P*_app_ provided a relatively notable positive impact on *QED* defined by *r* = 0.627 (Table S5).

The evaluation of mutual relationships between structural, physicochemical, pharmacokinetic and biological data would be very questionable in the current analyses. The reasons why biological parameters could not be taken into consideration were that not all compounds (particular salts) within the investigated set showed notable antimycobacterial efficacy and, if effective (especially against *Mtb* H_37_R_v_), a relatively narrow range of the *MIC* values was observed in vitro. In fact, approximately half of the screened molecules were characterized by *MIC* = 16–32 μM and several derivatives had the same *MIC*s determined against *Mtb* H_37_R_v_ (Table 1). Therefore, ‘an indirect approach’ could be chosen to investigate these relationships for the compounds (**IVb**), (**1b**)–(**4b**), (**7b**), (**8b**) and (**10b**)–(**12b**) whose salts showed *MIC*s ≤ 62.5 μM (Table 4 and Table S1). The details connected with such an analysis are provided in Section C (Supplementary Materials) and are visualized in Figure S9 (Supplementary Materials) as well.

### 4.4. In Vitro Screening of the Compounds (***I***)–(***IV***) Against Particular Mycobaterial Strains

Taking into account a relatively high ability of the derivatives (**3**), (**4**), (**7**), (**8**) and (**10**)–(**12**) to inhibit growth of *Mtb* H_37_R_v_ compared to inhibitory potency of (**IV**), the formation of additional beneficial interactions between more efficient ligands and appropriate effector sites (particular AAs) could be assumed. The research [7] allowed for formulation of a hypothesis that antimycobacterial potency of (**12**) might even be enhanced if the 2′-OCH_3_-moiety were shifted to the 3′-position of an aromatic core. This approach might contribute to less restricted geometry of the proposed derivative.

The precise and well-planned introduction of the *F*-atom(s) into a structure of compounds within the process of their design and development might notably contribute to the improvement in pharmacodynamic and pharmacokinetic features, as well as to the optimization of their selectivity profiles considering particular pharmacological targets [91]. The 2′-/4′-*F*-substitution not only modulated conformational or electronic characteristics [92] of respective derivatives but also increased their lipophilicity and chemical/metabolic stability. The incorporation of one or more *F*-atoms was beneficial for antimycobacterial efficiency because more effective permeation of such substituted compounds through lipophilic compartments of mycobacterial pathogens was observed [91,93]. The *F*-atom(s) could be found in the structure of various antimycobacterially effective agents [94] belonging to a class of 6-fluoroquinolones (ciprofloxacin or moxifloxacin could be mentioned as relevant examples), oxazolidinones (linezolid, sutezolid), arylquinolines (BDQ (**17**)), nitroimidazooxazines/nitroimidazoles (pretomanid, delamanid), benzothiazinones (macozinone) or imidazopyridines (telacebec).

Initial research focusing on several sets of synthetic variously substituted salicylanilides as well as corresponding bioisosteric compounds, and their in vitro antimycobacterial activity showed that an increase in anti-*Mtb* H_37_R_v_ efficacy of these derivatives was related to properties of a moiety attached to an aromatic ring within their amide portion. The most effective small-molecule antimycobacterial agents contained the substituent characterized by a relatively high value of the Hammett constant (*σ*), that is, electron-withdrawing features, and hydrophobic constant (*π*). In addition, linearity of these lipophilic molecules was considered a favorable structural property [95,96,97]. The given factors were reasons why the 4′-*F*-substituted derivatives (**1**)–(**4**) were previously prioritized in both antimycobacterial screening [6] and formal labeling (Figure 1) over the compounds (**5**)–(**12**).

The results published in [6] were not fully in agreement with the predictions and observations [7] taking into account the properties of particular substituents in an aromatic ring. The different conclusions might be attributed to different compositions of pharmacophores of the compounds (**1**)–(**8**) and derivatives (**13**)–(**16**) and/or more or less different mechanisms of their antimycobacterial action.

ATP synthase of various mycobacterial species belongs to a group of electrochemical transporter systems and serves as one of the key enzymes, securing proper energy metabolism of these pathogens [98]. Therefore, this ubiquitous enzyme is regarded as a very suitable target for drug candidates to be prospectively used for the treatment of TB [99].

Several suggestions on how to explain differences in antimycobacterial activity in vitro of presently analyzed compounds in a possible connection with their different abilities to interact with a given pharmacological target can be found in the following sections of this paper.

### 4.5. In Silico Investigation of Non-Protonated Molecules (***Ib***)–(***IVb***) and (***1b***)–(***12b***), as Well as Their Salts (***I***)–(***IV***) and (***1***)–(***12***)—Molecular Docking

Various efficient methods of computation-aided drug design, including MD analyses, notably contribute to effective development of pharmacologically active compounds and drugs [100]. The current docking analyses confirmed that particular non-protonated derivatives (**Ib**)–(**IVb**) and (**1b**)–(**12b**), as well as their salts (**I**)–(**IV**) and (**1**)–(**12**), docked into the same areas (binding pocket) of ATP synthase in *Mtb* H_37_R_v_ in a relatively similar fashion. The affinity of individual salts for this enzyme was, in general, higher compared to that of corresponding non-protonated compounds. This statement was based on calculated values of both MM-GBSA ΔG Bind and docking score parameters of these derivatives (Tables S6 and S7).

The advantage of the presently evaluated compounds would be that they could interact with both *a* and *c* subunits within a membrane-embedded polypeptide F_0_ domain of ATP synthase [101] according to the current analysis (Table S10). It might be very important to note that BDQ (**17**) was reported to interact with this enzyme through a ‘slightly’ different mode. This compound was bound within a unique site located at the interface between given subunits, preventing rotation of a so-called rotor ring (composed of several *c* subunits) of this enzyme and stopping the process of ATP synthesis [39,102,103]. In addition, the molecule (**17**) acted as an uncoupler of electron transport in an electron transport chain from ATP synthesis to ATP synthase [104]. In fact, preliminary MD procedures showed that the compounds (**Ib**)–(**IVb**), (**1b**)–(**12b**), (**I**)–(**IV**) and (**1**)–(**12**) interacted through that interface to effectively inhibit both *a* and *c* subunits of ATP synthase.

The mechanism of action based on inhibition of more than one subunit of this enzyme was also observed for compounds belonging to second-generation diarylquinolines. In that context, sorfequiline (TBAJ-876; **18**) [105,106,107], a preclinically evaluated analogue designed and structurally optimized on a platform of BDQ (**17**), would be mentioned as a suitable example. The chemical structure of (**18**), containing bulky 2,3,5-trimethoxypyridin-4-yl and 3,5-dimethoxypyridin-4-yl moieties, is provided in Figure 7.

On the other hand, the ability of BDQ (**17**) to directly interact with more than one subunit of a mycobacterial F_0_ domain still remains a hot topic for scientific debate [104]. The present research could also contribute to that discussion as listed in the last paragraphs of this section.

Theoretically, the most optimal *R*^1^-substituent in the structure of (*R*)-enantiomeric forms of (**I**)–(**IV**) was a linear C_3_H_7_-chain (Figure 1), very slightly preferring a given selection of the substituent over a linear C_4_H_9_-moiety (MM-GBSA ΔG Bind parameter of 68.12 kcal/mol versus 67.04 kcal/mol). The hydrophobic nature of the *R*^1^-group allowed formation of suitable hydrophobic interactions with the Val 2:39 AA residue present in the structure of the *c* subunit of ATP synthase.

Regarding the docking score parameters calculated for particular salts (Table S9), the situation was slightly different. The highest bonding potential to interact with ATP synthase was observed for the 4-NHCOOC_2_H_5_-substituted compounds, that is, (***R***)-(**II**), defined by a docking score value of −5.852 kcal/mol, and (***R***)-(**2**) that was characterized by a docking score output of −6.306 kcal/mol.

The receptor binding site of ATP synthase was stereospecific. The reason why particular (*R*)-enantiomers were more preferred and showed higher affinity for this target was that they had more convenient access to another AA residue, Ser *a*:77, to form a hydrogen bond through their OH-moiety.

The present MD evaluations also confirmed (Table S10) that a properly chosen substituent *R*^2^ (*R*^2^ ≠ H) attached to a 2′-position of the aromatic system was a more favorable alternative to improve anti-TB efficacy in vitro compared to the approach based on the introduction of 4′-F. That observation was in agreement with conclusions published in [7]. The 2′-OCH_3_-fragment in the structure of (**9**)–(**12**) formed particular hydrophobic interactions and hydrogen bonds with individual AA residues within an active site of ATP synthase (Table S10). This group also formed an electrostatic Coulombic interaction with a glutamic acid AA residue (Glu *a*:244); its length was 2.42 Å for the most effective antimycobacterial compound (***R***)-(**12**) (Table S11).

In fact, lower in vitro antimycobacterial activity of the derivatives (**I**)–(**IV**) could be explained by the absence of hydrogen bond, hydrophobic or ionic interactions with relevant AA residues within an active site of ATP synthase (Table S10). Such types of interactions were generated for the molecules (**1**)–(**12**) with 2′-/4′-*R*^2^ ≠ H (Figure 1) attached to their distal phenyl ring.

The structure of BDQ (**17**) contained two stereogenic centers (*C*-atoms; Figure 6), therefore, this compound could exist as the (**1*R***,**2*R***)-, (**1*R***,**2*S***)-, (**1*S***,**2*S***)- or (**1*S***,**2*R***)-stereoisomer. If theoretically accepting the possibility that all given enantiomers would interact with both *a* and *c* subunits of ATP synthase, the compound with the (**1*S***,**2*R***)-arrangement provided the most favorable binding affinity defined by MM-GBSA ΔG Bind = −46.21 kcal/mol (Table S14; Supplementary Materials).

The superior binding of this (**1*S***,**2*R***)-stereoisomer to ATP synthase might be attributed to favorable orientation of its substituted quinoline moiety within a binding pocket of the enzyme (Figure S10; Supplementary Materials). The 2-OCH_3_-substituted quinoline core established particular hydrophobic interactions with Glu 2:44 at distances of 3.12 Å, 3.64 Å and 3.85 Å, while a lipophilic 6-*Br*-atom formed hydrophobic interactions with Glu *a*:76. In addition to these interactions, a protonated amino group of (**1*S***,**2*R***)-(**17**) formed a hydrogen bond with the *O*-atom of a CO-group within Pro 2:43 (distance of 2.34 Å). On the other hand, this interaction was absent in the case of the (**1*R***,**2*S***)-stereoisomer, providing a structural explanation for higher binding affinity of (**1*S***,**2*R***)-(**17**).

The formation of an additional hydrogen bond was observed between the OH-group attached to the (**1*R***)-configured stereocenter and the *O*-atom of a CO-group of Pro 2:43 (distance of 1.97 Å). Finally, a phenyl ring attached to the (**2*S***)-configured stereocenter established hydrophobic contacts with hydrophobic backbones of arginine (Arg 3:48), integrated in an F_1_ complex of ATP synthase [101], and Pro 2:43 (Figure S10), further contributing to stabilization of interactions between the (**1*S***,**2*R***)-(**17**) ligand and protein. The mode in which the compound (**1*S***,**2*R***)-(**17**) was docked into an active site of ATP synthase present in *Mtb* H_37_R_v_ is provided in Figure S11 (Supplementary Materials).

Based on the current MD analyses, the approach to consider direct simultaneous interactions between BDQ (**17**) as well as its stereoisomers, and both *a* and *c* subunits ‘in an even manner’, potentially leading to anti-*Mtb* H_37_R_v_ properties, could not be valid. The real fact is that an officially approved highly efficient orally taken medicine contained (**1*R***,**2*S***)-(**17**) as a salt with fumaric acid [108].

The absolute values of calculated MM-GBSA energies (Table S14), that were lower compared to those of particular enantiomers of (**IV**) and (**12**), also (indirectly) confirmed that such ‘balanced’ direct simultaneous interactions (that is, without ‘evident’ preference for the *c* subunit) between BDQ (**17**), as well as its stereoisomers, and both subunits of ATP synthase would not be probable.

Therefore, the current results allowed us to propose a hypothesis that a properly substituted salt (**12**) could affect different sites within ATP synthase, possibly connected with its anti*-Mtb* H_37_R_v_ activity, compared to those affected by BDQ (**17**) and/or its stereoisomers. However, such a hypothesis has to be verified experimentally.

### 4.6. In Vitro Antiproliferative Properties of Chosen Compounds

The tested NAPs (**IV**) and (**12**), as well as INH, enhanced cellular conversion of MTT to formazan. Although the mechanism of MTT’s reduction to formazan is unclear and affected by confounding factors [54], it likely involves reactions with nicotinamide adenine dinucleotide or similar molecules that enable electron transfer. The formazan color indicated living cells but culture conditions that affect metabolism might influence the reduction rate.

The current research outlined that these derivatives moderately increased the MTT activity at relatively low *c*s and that feature was most evident in the case of INH (Figure S8). In fact, it preliminarily appeared that the proper substitution at the aromatic core in the 4-(substituted phenyl)piperazin-1-yl moiety (selection of *R*^2^ = 2′-OCH_3_, for example) of these NAPs would be a more accurate alternative in an attempt to synthesize non-toxic compounds compared to the introduction of ‘simple’ 4-phenylpiperazin-1-yl. On the other hand, the present in vitro experiments involving normal human dermal fibroblasts could be regarded as indicative and more detailed toxicological characteristics of prospective anti-TB agents should be studied employing other non-cancer and cancer cell lines. The fact is that several first-line anti-TB drugs officially approved for use in human medicine within various treatment regimes [109,110,111], that is, INH and RIF, showed significant cytotoxic and mutagenic responses. This unfavorable potential increased even more markedly when these compounds were involved in various combinations under in vitro experiments [112]. In addition, INH induced cytotoxicity and could cause notable changes in the cell cycle in mammalian cells [113].

### 4.7. In Silico Investigation of Non-Protonated Molecules (***Ib***)–(***IVb***) and (***1b***)–(***12b***)—Inspection of Their Possibilities to Inhibit Growth of Particular Mycobacteria

The Antibac-Pred applet [57] predicted several mycobacterial strains whose growth could be relatively effectively inhibited by the activity of the compounds (**Ib**)–(**IVb**) and (**1b**)–(**12b**). That evaluation could be helpful to roughly predict the perspectives of corresponding salts (**I**)–(**IV**) and (**1**)–(**12**) to act against other mycobacterial species.

The calculated score values (Table S12) indicated that the most susceptible strain would be the rapidly growing non-tuberculous *M. mageritense*. That prediction could be valid especially for the compounds belonging to the series (**Ib**)–(**IVb**), as well as (**9b**)–(**12b**), and (indirectly) for their salts. This pathogen is responsible for skin and soft tissue infections that could be treated with a combination of levofloxacin, a drug belonging to a third-generation 6-fluoroquinolone class, and doxycycline, as a second-generation broad-spectrum antibiotic from a group of tetracyclines. However, the success of this long-term pharmacological therapy was also connected with surgical intervention [114,115]. Therefore, the achievements in research focused on novel therapeutic modalities based on small-molecule drugs that might effectively target *M. mageritense* would be highly beneficial.

The introduction of the *F*-substituent into a structure of compounds slightly favored the possibility that such substituted molecules, excluding (**1b**) and (**5b**), would affect growth of *Mtb* H_37_R_a_ (Table S12). However, all these theoretical assumptions should be taken cautiously because a relatively low score value of < 0.3000 was generated in all cases. Thus, such predictions have to be verified under experimental conditions in vitro.

## 5. Conclusions

This research suggested a structural framework of currently analyzed (substituted) phenylcarbonyloxypiperazinopropanols as a promising starting platform to develop and optimize the NAP derivatives with enhanced activity against some mycobacterial pathogens, especially against virulent *Mtb* H_37_R_v_. Several in-silico-generated structural, physicochemical and pharmacokinetic features of the derivative (**12b**), whose salt (**12**) inhibited growth of *Mtb* H_37_R_v_ most effectively (Table S1), are summarized in Figure 8.

The values of selected descriptors (*MW*, *n*_ON_, *n*_OHNH_ and *n*_rotb_) calculated for (**12b**) were comparable to those of effective anti-TB agents. The predicted lipophilicity for this molecule was similar to calculated lipophilicity of pretomanid [62], a synthetic small-molecule drug containing a nitroimidazooxazine scaffold and acting as a mycolic acid biosynthesis inhibitor. Pretomanid is administered as part of so-called BPaL (consisting of BDQ (**17**), pretomanid and linezolid) and BPaMZ (BDQ (**17**), pretomanid, moxifloxacin and pyrazinamide) therapeutic regimens [116]. The derivative (**12b**) was more flexible than BDQ (**17**), and its lipophilicity was lower than lipophilicity of a given anti-TB agent or sorfequiline (**18**) defined by *c*log *P* = 5.15 [62]. The molecule (**12b**) was predicted to show very notably minimized ability (practically no ability, in fact) to passively permeate the BBB and enter the CNS.

With respect to the fundamental structural arrangement of currently investigated anti-*Mtb* H_37_R_v_ active molecules, for example, (**11**) and (**12**) (Figure 1, Table S1), a center of protonation (*N*-atom), stereospecifically oriented free OH-group attached to the 2-position of a connecting propan-1,3-diyl chain, suitable length of the NHCOO*R*^1^-chain and properly chosen substituent *R*^2^ at the aromatic ring within their 4-(substituted phenyl)piperazin-1-yl motif should be present to interact with ATP synthase. The obtained data allowed for the formulation of a hypothesis that a given enzyme in *Mtb* H_37_R_v_ would be a prospective biological target for such designed compounds. However, a more unambiguous conclusion could not be provided, as this proposal has to be supported by experimental validation data. The MD analyses indicated that a 2′-OCH_3_-group in the structure of (*R*)-(**12**) formed hydrophobic interactions and hydrogen bonds with particular AA residues present in an active site (binding pocket) of ATP synthase. This fragment also formed an electrostatic Coulombic interaction with a glutamic acid AA residue (Glu *a*:244).

Replacement of the 4-(substituted phenyl)piperazin-1-yl moiety by a suitable heteroaromatic fragment simultaneously with a bioisosteric replacement of a COO-group by a metabolically more stable *O*-bridge provided a series **MM** consisting of eight compounds (Figure 9). The in vitro anti-*Mtb* H_37_R_v_ efficacy of two derivatives from this set, containing *R* = C_3_H_7_ or C_4_H_9_ and X = CH, was comparable to anti-TB activity of INH [117]. Another study [118] concluded that an increase in lipophilicity within a series **IM** consisting of 27 structurally similar compounds (Figure 10) positively affected their in vitro anti-*Mtb* H_37_R_v_ efficiency. The modulation of lipophilic properties of individual derivatives was achieved through various combinations of both substituents *R*^1^ and *R*^2^.

The current research employing the compounds (**I**)–(**IV**) and (**1**)–(**12**) could also contribute to the refinement of a hypothesis about a possible mechanism of anti-H_37_R_v_ action of the derivatives belonging to both sets **MM** and **IM**. Thus, it would be proposed that their activity could be, among others, connected with non-covalent interactions with ATP synthase. The attempt to increase the antimycobacterial efficacy of particular compounds belonging to these series solely through ‘simple’ elongation of their alkoxycarbonyl or alkoxy fragments (alkoxy = methoxy to butoxy) in order to disrupt the cell wall integrity of these pathogens would not be regarded as a preferable alternative. The current research indicated that lipophilicity could not be an absolutely decisive factor to increase the activity of compounds inside mycobacteria, in agreement with observations at least in some chemical groups of antimycobacterial agents [119].

The more detailed specification of whether individual enantiomers of the molecules (**IV**) and (**12**), as well as anti-*Mtb* H_37_R_v_ active derivatives from both sets **MM** and **IM**, could interact with particular subunits of ATP synthase would be the goal for further experimental and in silico evaluations including the synthesis and screening of individual enantiomeric forms of these derivatives.

The present in silico analyses also suggested that other mycobacterial species, particularly *M. mageritense* and *M. tuberculosis* H_37_R_a_, might be susceptible to activity of the evaluated compounds.

Nevertheless, all these predictions should be interpreted very cautiously and confirmation employing relevant experimental studies would be required. Although some encouraging results were obtained, the *QED* scores calculated for individual non-protonated derivatives (**Ib**)–(**IVb**) and (**1b**)–(**12b**) of in vitro screened salts (**I**)–(**IV**) and (**1**)–(**12**) indicated that there was room to structurally optimize and improve their physicochemical properties.

## Figures and Tables

**Figure 3:**
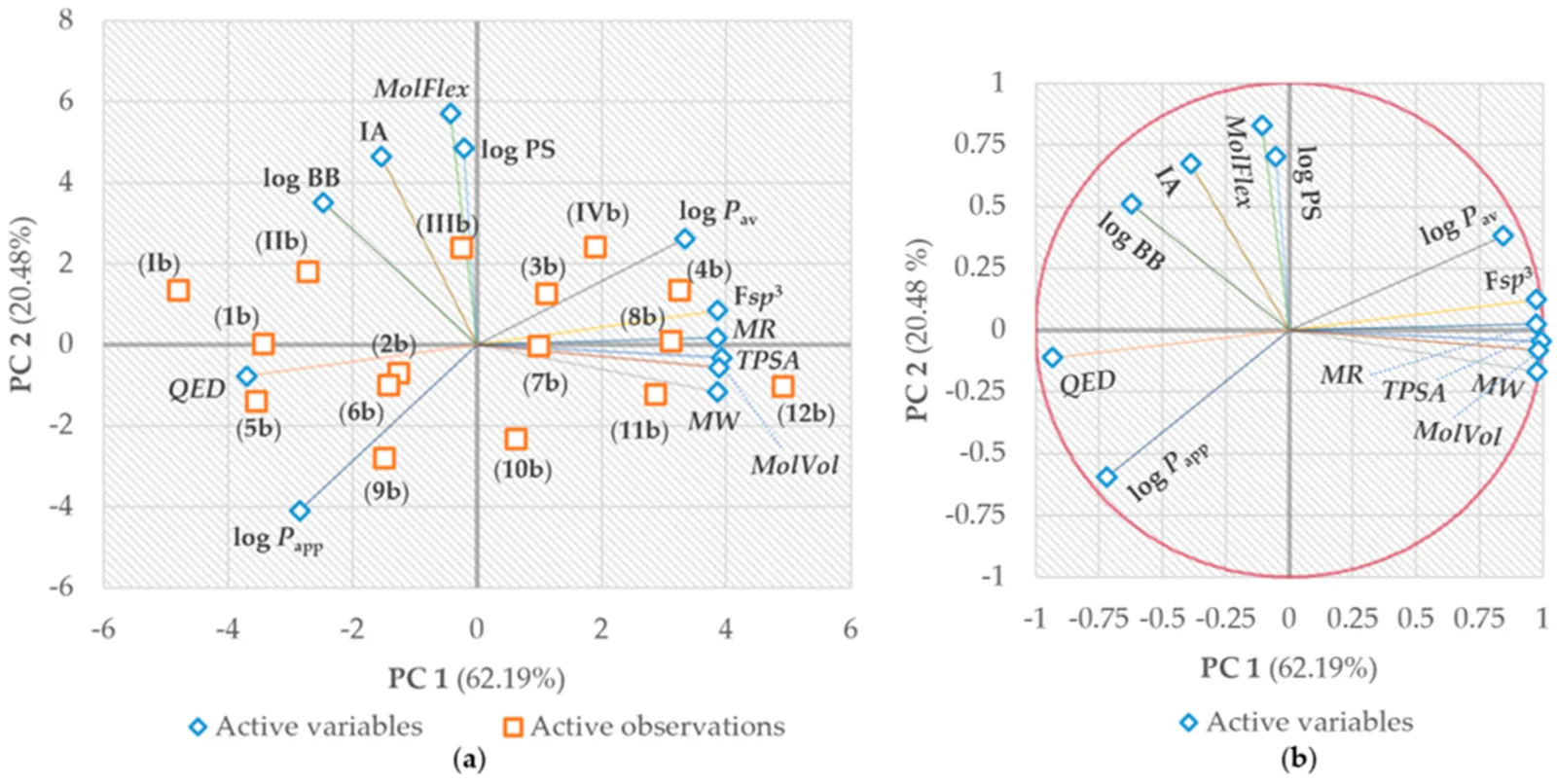
Graphical presentation of the relationships between structural, physicochemical and pharmacokinetic parameters of evaluated non-protonated derivatives (**Ib**)–(**IVb**) and (**1b**)–(**12b**): (**a**) 2D biplot mapping PC 1 and PC 2 scores of these compounds and loadings of particular variables, that is, *MW*, F*sp*^3^, *TPSA*, *MolFlex*, *MR*, *MolVol*, log *P*_av_, IA, log *P*_app_, log BB, log PS and *QED*; (**b**) 2D mapping of loadings of variables indicating their (i) positions within a circle of correlation; (ii) relationships with both PC 1 and PC 2.

**Figure 4:**
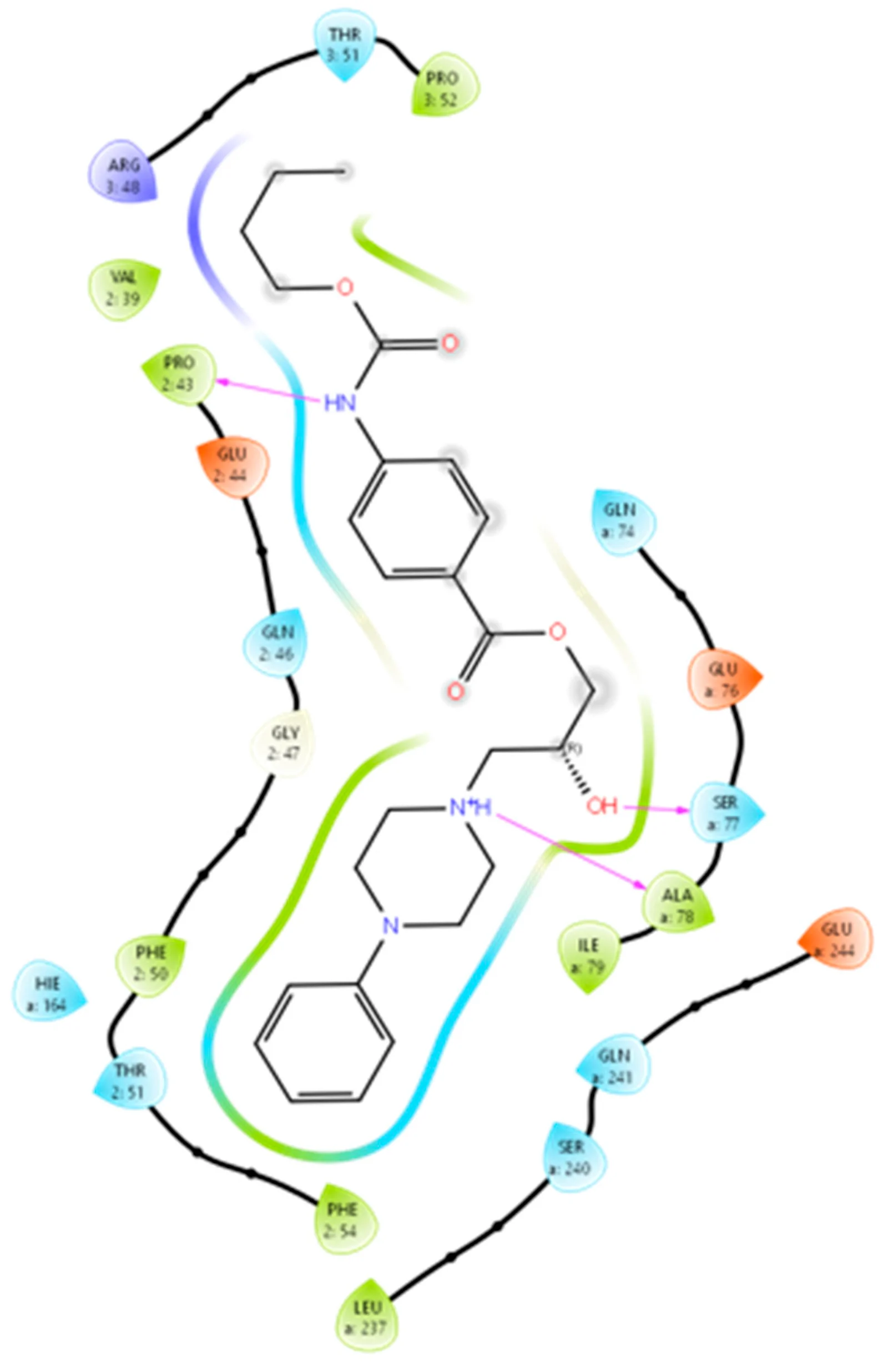
A 2D diagram showing the interactions between the compound (***R***)-(**IV**) and particular AA residues present within an active site (binding pocket) of ATP synthase in *Mtb* H_37_R_v_. Hydrophobic interactions are indicated in grey, purple arrows indicate hydrogen bonds.

**Figure 5:**
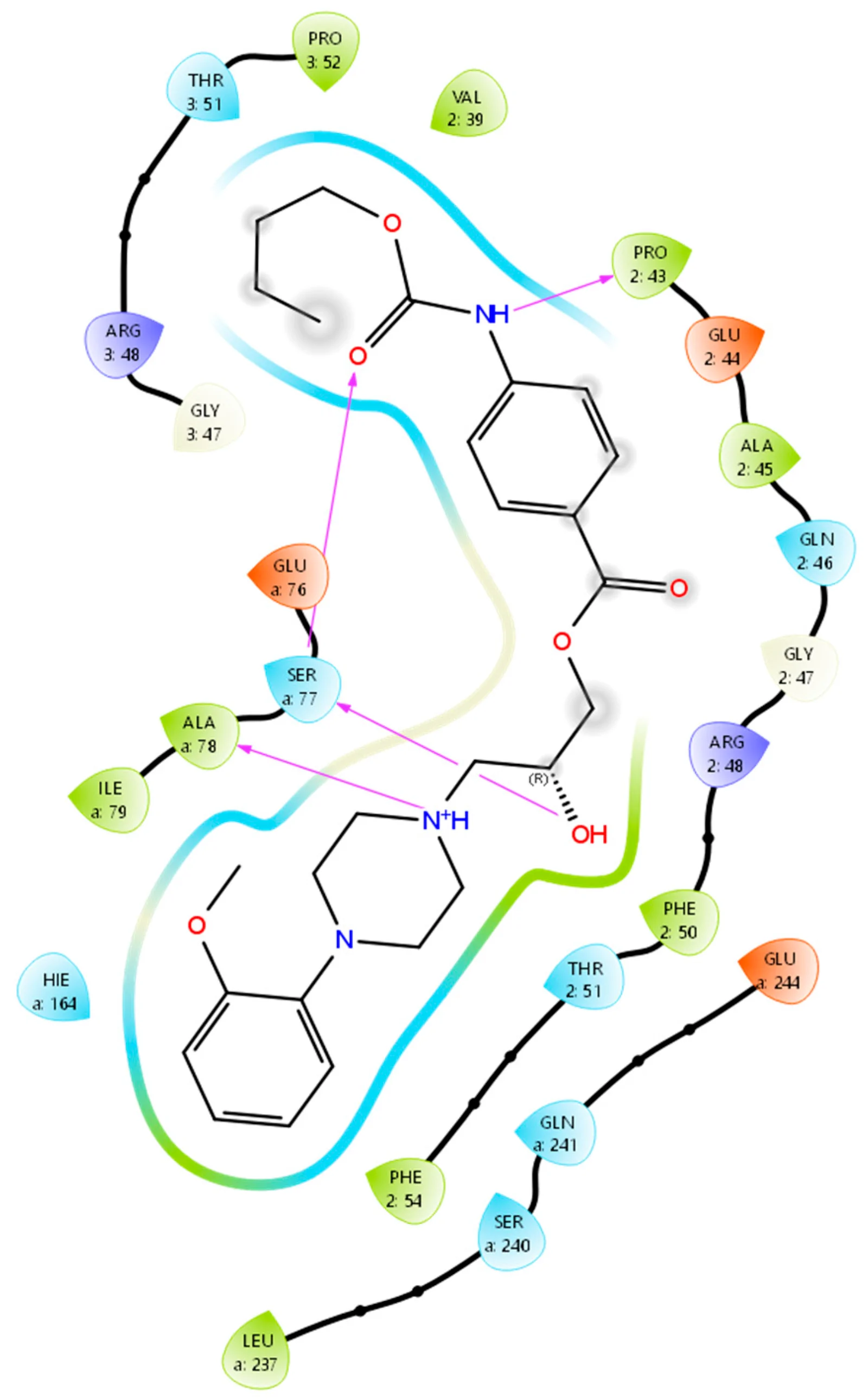
A 2D diagram showing the interactions between the compound (***R***)-(**12**) and particular AA residues present within an active site (binding pocket) of ATP synthase in *Mtb* H_37_R_v_. Hydrophobic interactions are indicated in grey, purple arrows indicate hydrogen bonds.

**Figure 6:**
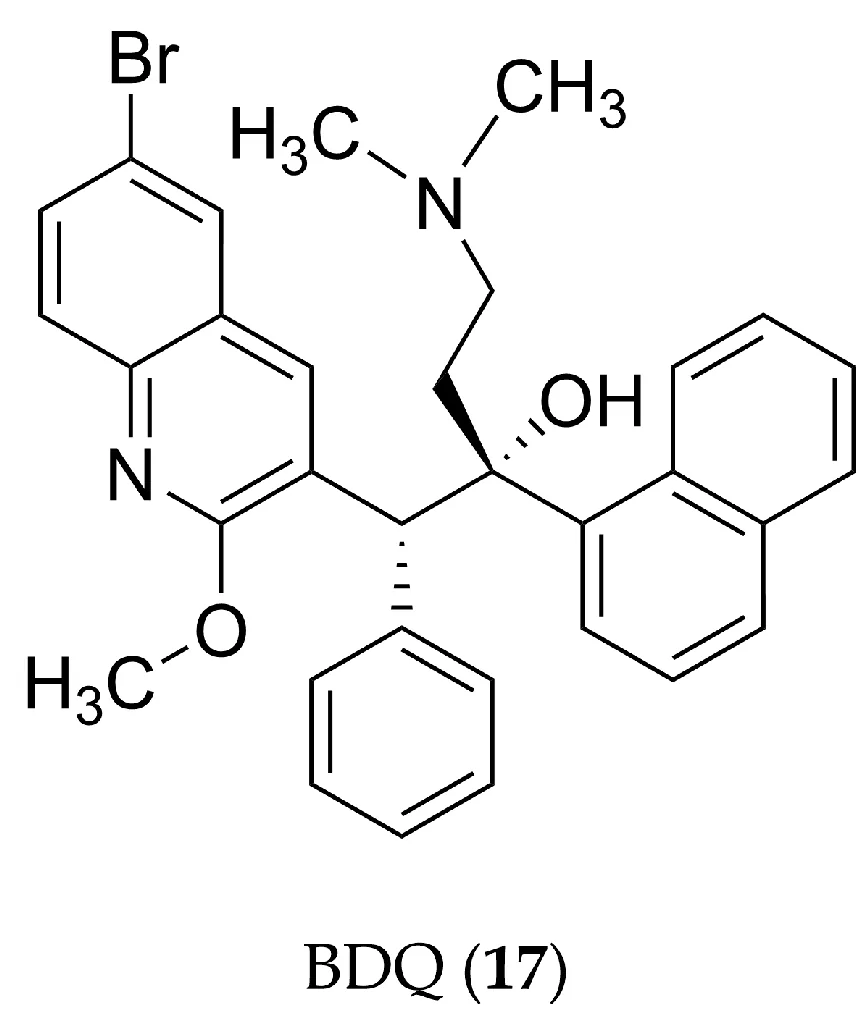
Chemical structure of bedaquiline (BDQ; **17**), an antimycobacterially effective drug [62].

**Figure 7:**
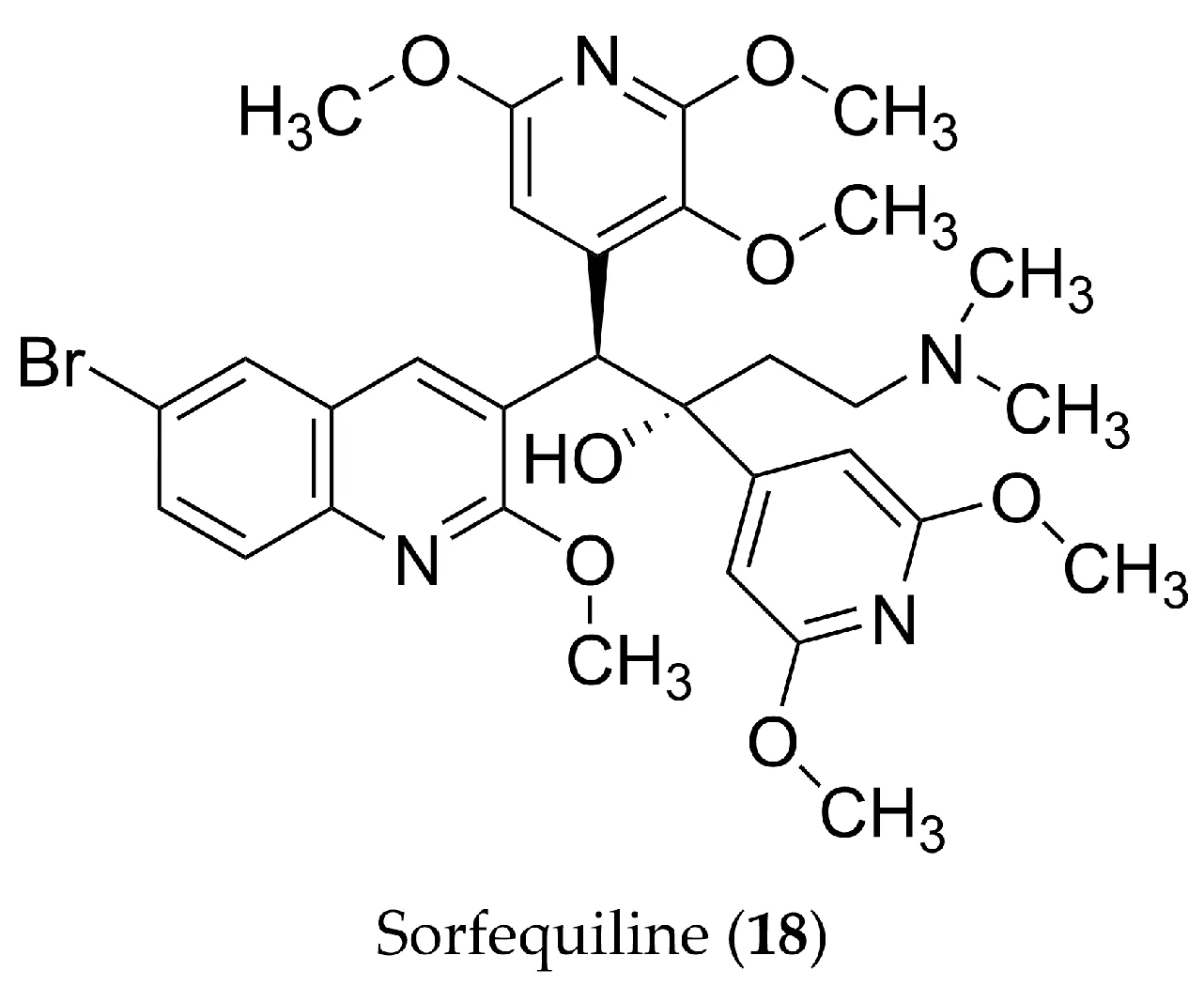
Chemical structure of sorfequiline (**18**), an antimycobacterially effective drug belonging to a second-generation diarylquinoline class [107].

**Figure 8:**
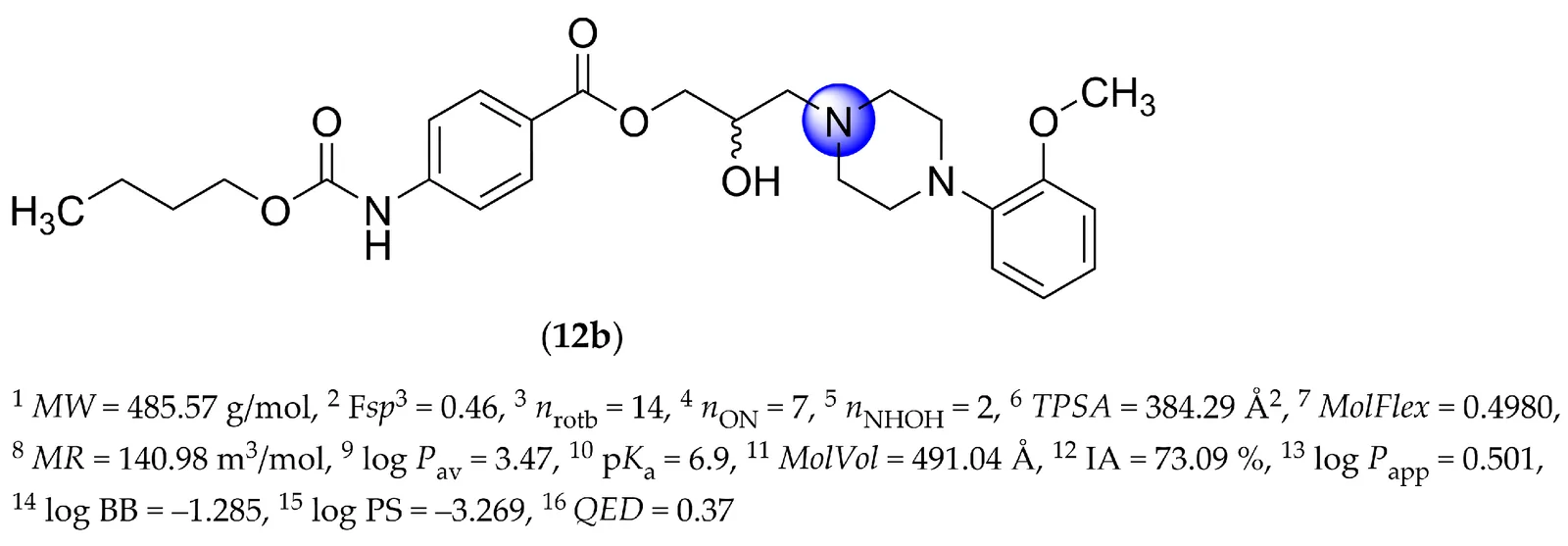
The predicted structural, physicochemical and pharmacokinetic characteristics of the compound (**12b**). ^1^ *MW* = molecular weight (in g/mol units), ^2^ F*sp*^3^ = fraction of *sp*^3^-hybridized *C*-atoms, ^3^ *n*_rotb_ = number of rotatable bonds, ^4^ *n*_ON_ = number of hydrogen-bond acceptors, ^5^ *n*_OHNH_ = number of hydrogen-bond donors, ^6^ *TSA* = total surface area (in Å^2^ units), ^7^ *MolFlex* = molecular flexibility, ^8^ *MR* = molar refractivity (in m^3^/mol units), ^9^ log *P*_av_ = average value of a decadic logarithm of a partition coefficient (*P*) value calculated from log *P* outputs of particular predictive methods for the octan-1-ol/water partition system, ^10^ p*K*_a_ = acid–base dissociation constant parameter predicted via the MolGpKa web server [12], ^11^ *MolVol* = molecular volume (in Å^3^ units) generated via the Molsoft L.L.C. applet [14], ^12^ IA (%) = intestinal absorption in humans (in percentages), ^13^ log *P*_app_ = decadic logarithm of an apparent permeability coefficient (*P*_app_), ^14^ log BB = logarithmic value of blood-to-brain ratio [11], ^15^ log PS = blood–brain barrier permeability–surface area product [11], ^16^ *QED* = quantitative estimate of drug-likeness [32]. The possibility to form respective enantiomers (considering the spatial arrangement of substituents attached to a stereogenic center (*C*-atom)) is indicated by a wavy bond. The *N*-atom that was chosen to be protonated within current in silico analyses is color-coded.

**Figure 9:**
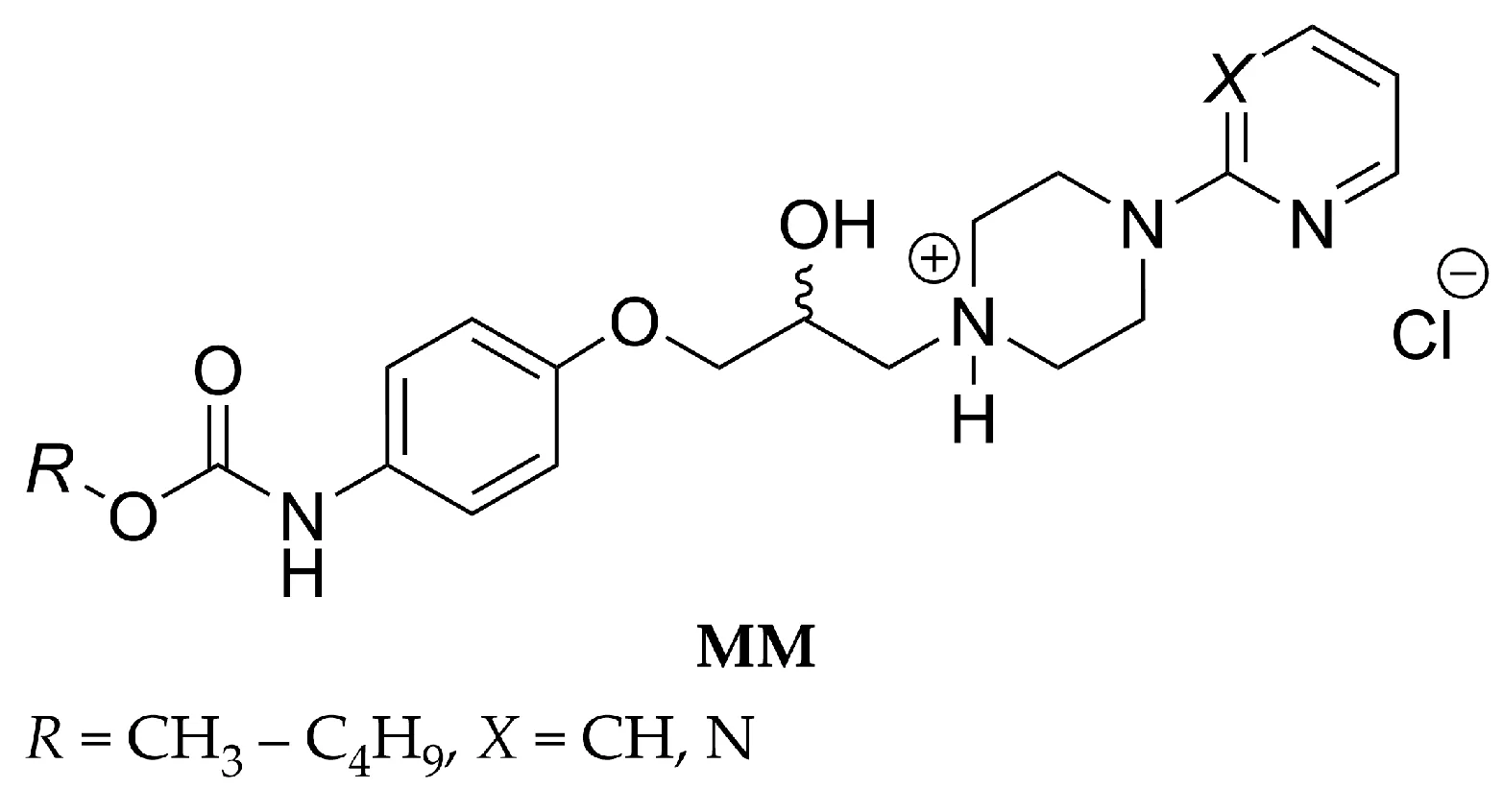
General chemical structure of the compounds from a series **MM** tested in vitro against a virulent *Mtb* H_37_R_v_ strain [117]. The possibility to form respective enantiomers (considering the spatial arrangement of substituents attached to a stereogenic center (*C*-atom)) is indicated by a wavy bond.

**Figure 10:**
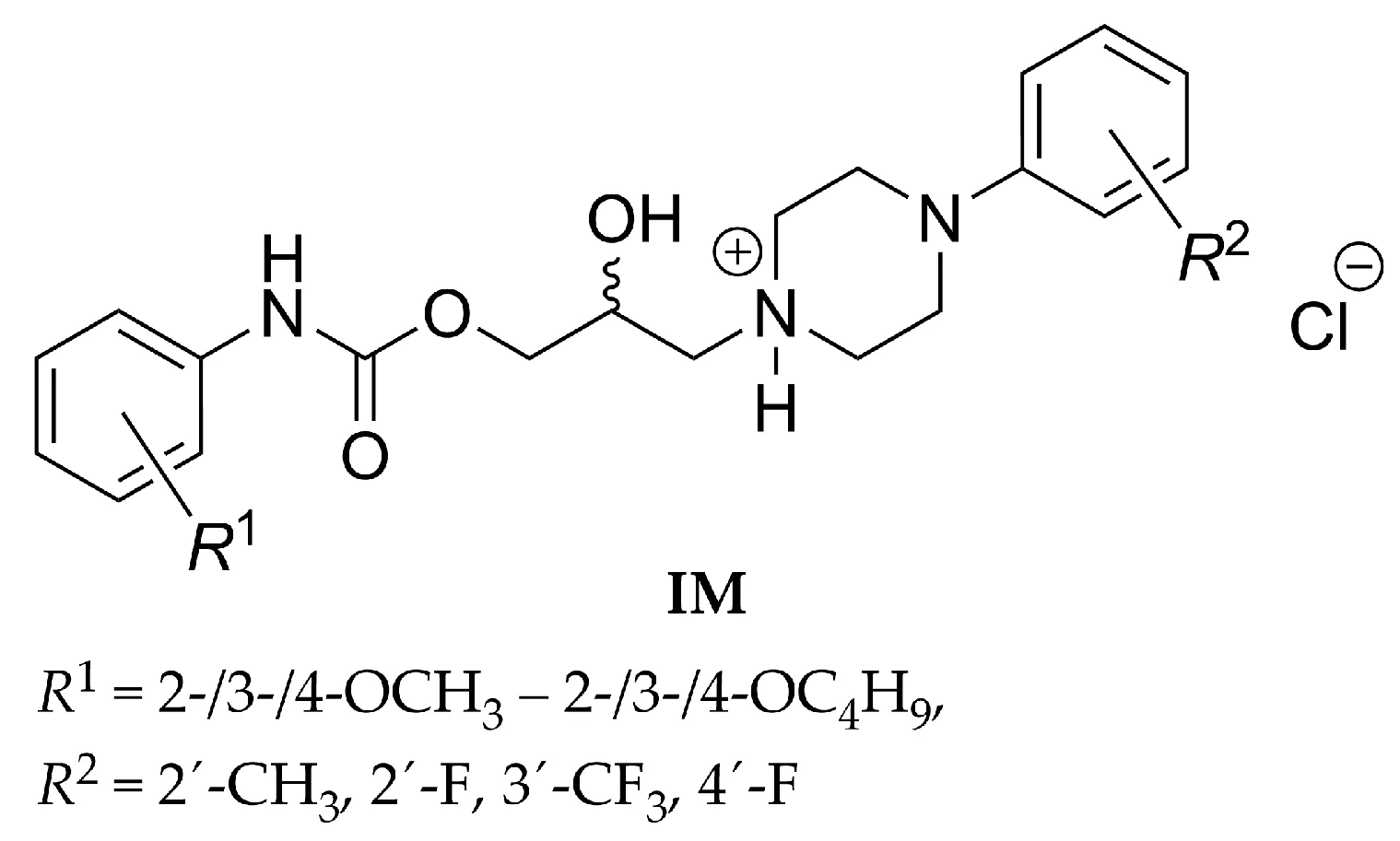
General chemical structure of the compounds from a series **IM** tested in vitro against a virulent *Mtb* H_37_R_v_ strain [118]. The possibility to form respective enantiomers (considering the spatial arrangement of substituents attached to a stereogenic center (*C*-atom)) is indicated by a wavy bond.

## Data Availability

The original contributions presented in this study are included in the article/Supplementary Materials. Further inquiries can be directed to the corresponding authors.

## Supplementary Materials

The following supporting information can be downloaded at: https://www.mdpi.com/article/10.3390/life16101593/s1, Table S1: The in vitro activity [6], expressed as the minimum inhibitory concentration values (*MIC*; in the μM units), of the compounds (**1**)–(**12**), as well as isoniazid (INH) reference drug against *Mycobacterium tuberculosis* H_37_R_v_ (*Mtb* H_37_R_v_), *M. kansasii* My 235/80 (*MK* My 235/80), *M. kansasii* 6509/96 clinical isolate (*MK*_ci_ 6509/96) and *M. avium* My 330/88 (*MA* My 330/88), respectively; Table S2: The formula and values of some fundamental physicochemical parameters of the compounds (**I**)–(**IV**) [16] and (**1**)–(**12**) [17,18]; Table S3: The Simplified Molecular Input Line Entry System (SMILES) codes of the compounds (**Ib**)–(**IVb**), (**1b**)–(**12b**), (**13**), (**14**) and bedaquiline (BDQ; **17**). The codes were generated via the SwissADME interactive tool [10]; Table S4: The prediction of phase I and phase II metabolites, that could be formed in humans, for the compounds (**Ib**)–(**IVb**) and (**1b**)–(**12b**) through the web-based GLORYx applet [30]. The priority score value, theoretically found within an interval of 0.00–1.00, was listed for selected metabolic pathways related to individual molecules; Table S5: The readouts from a chemometric principal component analysis (PCA) method exploring the relationships between structural, physicochemical and pharmacokinetic properties of analyzed compounds (**Ib**)–(**IVb**) and (**1b**)–(**12b**) through the XLSTAT ver. 2019 2.2 statistical software add-in (Addinsoft, New York, NY, USA) for Microsoft Excel (Microsoft 365; Microsoft Corp., Redmont, WA, USA). The relationships are described by positive or negative values of Pearson’s correlation coefficient (*r*); Table S6: The MM-GBSA energies (MM-GBSA ΔG Bind parameter; in kcal/mol units) calculated for particular (*R*)- and (*S*)-enantiomeric forms of non-protonated compounds (**Ib**)–(**IVb**) and (**1b**)–(**12b**) that were docked into ATP synthase; Table S7: The MM-GBSA energies (MM-GBSA ΔG Bind parameter; in kcal/mol units) calculated for particular (*R*)- and (*S*)-enantiomeric forms of in vitro screened salts (**I**)–(**IV**) and (**1**)–(**12**) that were docked into ATP synthase; Table S8: The ligand docking score values (in kcal/mol units) calculated for particular (*R*)- and (*S*)-enantiomeric forms of non-protonated compounds (**Ib**)–(**IVb**) and (**1b**)–(**12b**) that were docked into ATP synthase; Table S9: The ligand docking score values (in kcal/mol units) calculated for particular (*R*)- and (*S*)-enantiomeric forms of in vitro screened salts (**I**)–(**IV**) and (**1**)–(**12**) that were docked into ATP synthase; Table S10: The interactions between substituents (atoms/group) attached to a distal phenyl ring of the compounds (**I**)–(**IV**) and (**1**)–(**12**) in the position 2′ and/or 4′, and particular amino acids (AAs) present in an active site of ATP synthase; Table S11: The individual stereoisomers of the ligands (**IV**) and (**12**), particular groups in their structure interacting with individual amino acids (AAs) present in an active site of ATP synthase and interaction lengths (in Å units); Table S12: The prediction of score for the compounds (**Ib**)–(**IVb**) and (**1b**)–(**12b**), describing their theoretical ability to inhibit growth of selected mycobacterial strains, namely, *Mycobacterium mageritense*, avirulent *M. tuberculosis* H_37_R_a_ (*Mtb* H_37_R_a_), *M. bovis* BCG, resistant *M. tuberculosis* H_37_R_v_ (*Mtb* H_37_R_v-r_), resistant *M. ulcerans* (*M. ulcerans*_r_), resistant *M. avium* (*M. avium*_r_) and *M. phlei*, using the web-based Antibac-Pred applet [57]; Table S13: The readouts from a chemometric principal component analysis (PCA) method used to explore the relationships between structural, physicochemical and pharmacokinetic properties of non-protonated compounds (**IVb**), (**1b**)–(**4b**), (**7b**), (**8b**) and (**10b**)–(**12b**) through the XLSTAT ver. 2019 2.2 statistical software add-in (Addinsoft, New York, NY, USA) for Microsoft Excel (Microsoft 365; Microsoft Corp., Redmont, WA, USA). The relationships are described by positive or negative values of Pearson’s correlation coefficient (*r*); Table S14: The MM-GBSA energies (MM-GBSA ΔG Bind parameter; in kcal/mol units) calculated for bedaquiline (BDQ; **17**) and its stereoisomeric forms that were docked into ATP synthase; Figure S1: The binding mode of the compound (***R***)-(**IV**) within an active site of ATP synthase present in *Mtb* H_37_R_v_; Figure S2: The binding mode of the compound (***R***)-(**12**) within an active site of ATP synthase present in *Mtb* H_37_R_v_; Figure S3: The effect of different concentrations of the compound (**IV**) on normal human dermal fibroblast viability (in percentages); Figure S4: The effect of different concentrations of the compound (**12**) on normal human dermal fibroblast viability (in percentages); Figure S5: The effect of different concentrations of isoniazid (INH) on normal human dermal fibroblast viability (in percentages); Figure S6: The (**a**) non-treated normal human dermal fibroblast cells (control) and normal human dermal fibroblasts treated with the compound (**IV**) at (**b**) *c* = 125 μM and (**c**) *c* = 250 μM and incubated with a 3-(4,5-dimethylthiazol-2-yl)-2,5-diphenyl-2*H*-tetrazolium bromide (MTT) reagent; Figure S7: The (**a**) non-treated normal human dermal fibroblast cells (control) and normal human dermal fibroblasts treated with the compound (**12**) at (**b**) *c* = 125 μM and (**c**) *c* = 250 μM and incubated with a 3-(4,5-dimethylthiazol-2-yl)-2,5-diphenyl-2*H*-tetrazolium bromide (MTT) reagent; Figure S8: The (**a**) non-treated normal human dermal fibroblast cells (control) and normal human dermal fibroblasts treated with isoniazid (INH) at (**b**) *c* = 125 μM and (**c**) *c* = 250 μM and incubated with a 3-(4,5-dimethylthiazol-2-yl)-2,5-diphenyl-2*H*-tetrazolium bromide (MTT) reagent; Figure S9: Graphical presentation of the relationships between structural, physicochemical and pharmacokinetic parameters of selected non-protonated compounds whose salts relatively effectively inhibited growth of *Mtb* H_37_R_v_: (**a**) 2D biplot mapping PC 1 and PC 2 scores of these compounds and loadings of particular variables, that is, *MW*, F*sp*^3^, *TPSA*, *MolFlex*, *MR*, *MolVol*, log *P*_av_, IA, log *P*_app_, log BB, log PS and *QED*; (**b**) 2D mapping of loadings of variables indicating their (i) positions within a circle of correlation; (ii) relationships with both PC 1 and PC 2; Figure S10: The 2D diagram showing particular interactions between the (**1*S***,**2*R***)-stereoisomer of bedaquiline (BDQ; **17**) and individual amino acid (AA) residues present within an active site (binding pocket) of ATP synthase in *Mtb* H_37_R_v_. Hydrophobic interactions are indicated in grey, purple arrows indicate hydrogen bonds; Figure S11: The mode in which the (**1*S***,**2*R***)-stereoisomer of bedaquiline (BDQ; **17**) was docked into an active site of ATP synthase present in *Mtb* H_37_R_v_.

## Abbreviations

The following abbreviations are used in this manuscript:

AA(s)	Amino acid(s)
AMK	Amikacin (second-line antituberculosis drug)
anti-TB	Antituberculosis
ATP synthase	Adenosine 5′-triphosphate synthase
BBB	Blood–brain barrier
BDQ	Bedaquiline (novel-class second-line antituberculosis drug)
CLOGP 4.0	Lipophilicity parameter (log *P*) calculated via a fragmental CLOGP 4.0 method
CNS	Central nervous system
F*sp*^3^	Fraction of *sp*^3^-hybridized *C*-atoms
IA	Intestinal absorption in humans (parameter; in percentages)
INH	Isoniazid (first-line antituberculosis drug)
log BB	Logarithmic value of blood-to-brain ratio
log *P*	Decadic logarithm of a partition coefficient (*P*) calculated for an octan-1-ol/water partition system, or estimated for an octan-1-ol/buffer (p*H* = 7.0) partition system
log *P*_app_	Logarithmic value of an apparent permeability coefficient (*P*_app_)
log PS	Permeability–surface area product
*MA* My 330/80	*Mycobacterium avium* CNCTC My 330/80
MD	Molecular docking
*MIC*	Minimum inhibitory concentration (in μM units)
*MK* My 235/80	*Mycobacterium kansasii* CNCTC My 235/80
*MK*_ci_ 6509/96	*Mycobacterium kansasii* 6509/96 clinical isolate
MLOGP	Lipophilicity parameter (log *P*) calculated via Moriguchi’s method
MM-GBSA	Molecular mechanics–generalized born surface area (method)
*MR*	Molar refractivity (in m^3^/mol units)
*Mtb* H_37_R_a_	*Mycobacterium tuberculosis* H_37_R_a_ (avirulent mycobacterial strain)
*Mtb* H_37_R_v_	*Mycobacterium tuberculosis* H_37_R_v_ (virulent mycobacterial strain)
MTT	3-(4,5-Dimethylthiazol-2-yl)-2,5-diphenyl-2*H*-tetrazolium bromide
*MW*	Molecular weight (in g/mol units or in Da units)
*n*_OHNH_	Number of hydrogen-bond donors
*n*_ON_	Number of hydrogen-bond acceptors
*n*_rotb_	Number of rotatable bonds
NAP(s)	*N*-Arylpiperazine(s)
OD	Optical density
OPLS4	Optimized potentials for liquid simulations 4th generation
p*K*_a_	Acid–base dissociation constant
PCA	Principal component analysis
*QED*	Quantitative estimate of drug-likeness (parameter)
RIF	Rifampicin (first-line antituberculosis drug)
SMILES	Simplified Molecular Input Line Entry System
*tPSA*	Topological polar surface area (in A^2^ units)
TB	Tuberculosis
*TSA*	Total (molecular) surface area (in A^2^ units)
WLOGP	Lipophilicity parameter (log *P*) calculated via Wildman and Crippen’s atomic-based method
XLOGP3	Lipophilicity parameter (log *P*) calculated via an atomic/group-based XLOGP3 method
