# Supplementary material for: Integrated Structural, Physicochemical, Pharmacokinetic and Pharmacodynamic Evaluation of Substituted Phenylcarbonyloxypiperazinopropanols as Antimycobacterial Agents
Source: Life (Basel). 2026 Sep 23;16(10):1593. doi: 10.3390/life16101593 (PMC13705932; doi:10.3390/life16101593)
Supplement: Supplementary file 1 [file life-16-01593-s001.zip › life-4545027-supplementary.pdf]

*Supplementary Materials*

# Integrated Structural, Physicochemical, Pharmacokinetic and Pharmacodynamic Evaluation of Substituted Phenylcarbonyloxypiperazinopropanols as Antimycobacterial Agents


**Dominika Nádaská [1,*], Tomáš Sádecký [1], Lucia Račková [2,3,4], Martina Hrčka Dubničková [5], Fils Andriamainty [1], Petr Mokrý [6], Simona Gover [1], Vladimír Garaj [1], Jozef Csöllei [6] and Ivan Malík [1,*]**

[1] Department of Pharmaceutical Chemistry, Faculty of Pharmacy, Comenius University Bratislava, Odbojárov 10, P.O.BOX 90, SK-820 18 Bratislava 218, Slovakia; sadecky8@uniba.sk (T.S.); andriamainty@fpharm.uniba.sk (F.A.); garaj1@uniba.sk (V.G.)

[2] Institute of Chemistry, Slovak Academy of Sciences, Dúbravská cesta 9, SK-845 38 Bratislava, Slovakia; lucia.rackova@savba.sk (L.R.)

[3] Department of Pharmacognosy and Botany, Faculty of Pharmacy, Comenius University Bratislava, Odbojárov 10, P.O.BOX 90, SK-820 18 Bratislava 218, Slovakia

[4] Centre of Experimental Medicine, Institute of Experimental Pharmacology and Toxicology, Slovak Academy of Sciences, Dúbravská cesta 9, SK-841 04 Bratislava, Slovakia

[5] Department of Cell and Molecular Biology of Drugs, Faculty of Pharmacy, Comenius University Bratislava, Odbojárov 10, P.O.BOX 90, SK-820 18 Bratislava 218, Slovakia; martina.hrcka.dubnickova@uniba.sk (M.H.D.)

[6] Department of Chemical Drugs, Faculty of Pharmacy, Masaryk University in Brno, Palackého třída 1946/1, CZ-612 00 Brno, Czech Republic; mokryp@pharm.muni.cz (P.M.); csolleij@pharm.muni.cz (J.C.)

* Correspondence: nadaska11@uniba.sk (D.N.), malik2@uniba.sk (I.M.)


## CONTENT

## A. TABLES

**Table S1.** The in vitro activity [6], expressed as the minimum inhibitory concentration values (*MIC*; in the μM units), of the compounds (**1**)–(**12**), as well as isoniazid (INH) reference drug against *Mycobacterium tuberculosis* $H_{37}R_v$ (*Mtb* $H_{37}R_v$), *M. kansasii* My 235/80 (*MK* My 235/80), *M. kansasii* 6 509/96 clinical isolate ($MK_{ci}$ 6 509/96) and *M. avium* My 330/88 (*MA* My 330/88), respectively.

**Table S2.** The formula and values of some fundamental physicochemical parameters of the compounds (**I**)–(**IV**) [16] and (**1**)–(**12**) [17,18].

**Table S3.** The Simplified Molecular Input Line Entry System (SMILES) codes of the compounds (**Ib**)–(**IVb**), (**1b**)–(**12b**), (**13**), (**14**) and bedaquiline (BDQ; **17**). The codes were generated via the SwissADME interactive tool [10].

**Table S4.** The prediction of phase I and phase II metabolites, that could be formed in humans, for the compounds (**Ib**)–(**IVb**) and (**1b**)–(**12b**) through the web-based GLORYx applet [30]. The priority score value, theoretically found within an interval of 0.00–1.00, was listed for selected metabolic pathways related to individual molecules.

**Table S5.** The readouts from a chemometric principal component analysis (PCA) method exploring the relationships between structural, physicochemical and pharmacokinetic properties of analyzed compounds (**Ib**)–(**IVb**) and (**1b**)–(**12b**) through the XLSTAT ver. 2019 2.2 statistical software add-in (Addinsoft, New York, NY, USA) for Microsoft Excel (Microsoft 365; Microsoft Corp., Redmont, WA, USA). The relationships are described by positive or negative values of Pearson's correlation coefficient (*r*).

**Table S6.** The MM-GBSA energies (MM-GBSA ΔG Bind parameter; in kcal/mol units) calculated for particular (*R*)- and (*S*)-enantiomeric forms of non-protonated compounds (**Ib**)–(**IVb**) and (**1b**)–(**12b**) that were docked into ATP synthase.

**Table S7.** The MM-GBSA energies (MM-GBSA ΔG Bind parameter; in kcal/mol units) calculated for particular (*R*)- and (*S*)-enantiomeric forms of in vitro screened salts (**I**)–(**IV**) and (**1**)–(**12**) that were docked into ATP synthase.

**Table S8.** The ligand docking score values (in kcal/mol units) calculated for particular (*R*)- and (*S*)-enantiomeric forms of non-protonated compounds (**Ib**)–(**IVb**) and (**1b**)–(**12b**) that were docked into ATP synthase.

**Table S9.** The ligand docking score values (in kcal/mol units) calculated for particular (*R*)- and (*S*)-enantiomeric forms of in vitro screened salts (**I**)–(**IV**) and (**1**)–(**12**) that were docked into ATP synthase.

**Table S10.** The interactions between substituents (atoms / group) attached to a distal phenyl ring of the compounds (**I**)–(**IV**) and (**1**)–(**12**) in the position 2′ and/or 4′, and particular amino acids (AAs) present in an active site of ATP synthase.

**Table S11.** The individual stereoisomers of the ligands (**IV**) and (**12**), particular groups in their structure interacting with individual amino acids (AAs) present in an active site of ATP synthase and interaction lengths (in Å units).

**Table S12.** The prediction of score for the compounds (**Ib**)–(**IVb**) and (**1b**)–(**12b**), describing their theoretical ability to inhibit growth of selected mycobacterial strains, namely, *Mycobacterium mageritense*, avirulent *M. tuberculosis* $H_{37}R_a$ (*Mtb* $H_{37}R_a$), *M. bovis* BCG, resistant *M. tuberculosis* $H_{37}R_v$ (*Mtb* $H_{37}R_{v\text{-}r}$), resistant *M. ulcerans* (*M. ulcerans*$_r$),

resistant *M. avium* (*M. avium*$_r$) and *M. phlei*, using the web-based AntibacPred applet [57].

**Table S13.** The readouts from a chemometric principal component analysis (PCA) method used to explore the relationships between structural, physicochemical and pharmacokinetic properties of non--protonated compounds (**IVb**), (**1b**)–(**4b**), (**7b**), (**8b**) and (**10b**)–(**12b**) through the XLSTAT ver. 2019 2.2 statistical software add-in (Addinsoft, New York, NY, USA) for Microsoft Excel (Microsoft 365; Microsoft Corp., Redmont, WA, USA). The relationships are described by positive or negative values of Pearson's correlation coefficient (*r*).

**Table S14.** The MM-GBSA energies (MM-GBSA ΔG Bind parameter; in kcal/mol units) calculated for bedaquiline (BDQ; **17**) and its stereoisomeric forms that were docked into ATP synthase

## B. NOTES

## C. FIGURES

**Figure S1.** The binding mode of the compound (***R***)-(**IV**) within an active site of ATP synthase present in *Mtb* $H_{37}R_v$.

**Figure S2.** The binding mode of the compound (***R***)-(**12**) within an active site of ATP synthase present in *Mtb* $H_{37}R_v$.

**Figure S3.** The effect of different concentrations of the compound (**IV**) on normal human dermal fibroblast viability (in percentages).

**Figure S4.** The effect of different concentrations of the compound (**12**) on normal human dermal fibroblast viability (in percentages).

**Figure S5.** The effect of different concentrations of isoniazid (INH) on normal human dermal fibroblast viability (in percentages).

**Figure S6.** The (**a**) non-treated normal human dermal fibroblast cells (control) and normal human dermal fibroblasts treated with the compound (**IV**) at (**b**) $c$ = 125 μM and (**c**) $c$ = 250 μM and incubated with a 3-(4,5-dimethylthiazol-2-yl)-2,5-diphenyl-2*H*-tetrazolium bromide (MTT) reagent.

**Figure S7.** The (**a**) non-treated normal human dermal fibroblast cells (control) and normal human dermal fibroblasts treated with the compound (**12**) at (**b**) $c$ = 125 μM and (**c**) $c$ = 250 μM and incubated with a 3-(4,5-dimethylthiazol-2-yl)-2,5-diphenyl-2*H*-tetrazolium bromide (MTT) reagent.

**Figure S8.** The (**a**) non-treated normal human dermal fibroblast cells (control) and normal human dermal fibroblasts treated with isoniazid (INH) at (**b**) $c$ = 125 μM and (**c**) $c$ = 250 μM and incubated with a 3-(4,5-

-dimethylthiazol-2-yl)-2,5-diphenyl-2*H*-tetrazolium bromide (MTT) reagent.

**Figure S9.** Graphical presentation of the relationships between structural, physicochemical and pharmacokinetic parameters of selected non--protonated compounds which salts relatively effectively inhibited growth of *Mtb* $H_{37}R_v$: (**a**) 2D biplot mapping PC 1 and PC 2 scores of these compounds and loadings of particular variables, that is, *MW*, F$sp^3$, *TPSA*, *MolFlex*, *MR*, *MolVol*, log $P_{av}$, IA, log $P_{app}$, log BB, log PS and *QED*; (**b**) 2D mapping of loadings of variables indicating their (i) positions within a circle of correlation; (ii) relationships with both PC 1 and PC 2.

**Figure S10.** The 2D diagram showing particular interactions between the (**1*S***,**2*R***)-stereoisomer of bedaquiline (BDQ; **17**) and individual amino acid (AA) residues present within an active site (binding pocket) of ATP synthase in *Mtb* $H_{37}R_v$. Hydrophobic interactions are indicated in grey, purple arrows indicate hydrogen bonds.

**Figure S11.** The mode in which the (**1*S***,**2*R***)-stereoisomer of bedaquiline (BDQ; **17**) was docked into an active site of ATP synthase present in *Mtb* $H_{37}R_v$.

**A. TABLES**

**Table S1.** The in vitro activity [6], expressed as the minimum inhibitory concentration values (*MIC*; in the µM units), of the compounds (**1**)–(**12**), as well as isoniazid (INH) reference drug against *Mycobacterium tuberculosis* $H_{37}R_v$ (*Mtb* $H_{37}R_v$), *M. kansasii* My 235/80 (*MK* My 235/80), *M. kansasii* 6 509/96 clinical isolate (*MK*ci 6 509/96) and *M. avium* My 330/88 (*MA* My 330/88), respectively.

| [1] **Cmpd.** | ***MIC* (µM)** | | | | | | | |
|---|---|---|---|---|---|---|---|---|
| | ***Mtb* $H_{37}R_v$** | | ***MK* My 235/80** | | ***MK*ci 6 509/96** | | ***MA* My 330/88** | |
| | [2] **14 d** | [3] **21 d** | **14 d** | **21 d** | **14 d** | **21 d** | **14 d** | **21 d** |
| (**1**) | 62.5 | 125 | 250 | 250 | 125 | 250 | 125 | 250 |
| (**2**) | 62.5 | 125 | 125 | 250 | 125 | 250 | 62.5 | 250 |
| (**3**) | 32 | 62.5 | 125 | 125 | 62.5 | 125 | 125 | 250 |
| (**4**) | 32 | 32 | 62.5 | 62.5 | 32 | 62.5 | 32 | 62.5 |
| (**5**) | 125 | 250 | 250 | 500 | 62.5 | 125 | 250 | 500 |
| (**6**) | 125 | 250 | 125 | 125 | 125 | 125 | 125 | 250 |
| (**7**) | 32 | 62.5 | 125 | 125 | 62.5 | 125 | 62.5 | 125 |
| (**8**) | 32 | 32 | 62.5 | 125 | 32 | 62.5 | 62.5 | 125 |
| (**9**) | 125 | 250 | 500 | > 500 | 125 | 250 | 250 | 250 |
| (**10**) | 32 | 62.5 | 500 | > 500 | 62.5 | 125 | 250 | 250 |
| (**11**) | 32 | 62.5 | 125 | 125 | 32 | 62.5 | 125 | 250 |
| (**12**) | 16 | 32 | 62.5 | 125 | 32 | 32 | 125 | 125 |
| INH | 0.5 | 0.5 | > 250 | > 250 | 2 | 4 | > 250 | > 250 |

[1] Cmpd. = compound, [2] 14 d = 14-day cultivation, [3] 21 d = 21-day cultivation.

**Table S2.** The formula and values of some fundamental physicochemical parameters of the compounds (**I**)–(**IV**) [16] and (**1**)–(**12**) [17,18].

| [1] Cmpd. | Formula | [2] *MW* (g/mol) | [3] mp (°C) | [4] log *P* | [5] p$K_a$ |
|---|---|---|---|---|---|
| (**I**) | $C_{22}H_{27}O_5ClN_3$ | 448.93 | 195–198 | [6] *nd* | 4.8 |
| (**II**) | $C_{23}H_{29}O_5ClN_3$ | 462.95 | 192–194 | *nd* | 4.6 |
| (**III**) | $C_{24}H_{31}O_5ClN_3$ | 476.98 | 189–191 | *nd* | 5.0 |
| (**IV**) | $C_{25}H_{33}O_5ClN_3$ | 491.01 | 178–181 | *nd* | 5.0 |
| (**1**) | $C_{22}H_{27}O_5FClN_3$ | 467.92 | 175–178 | 3.52 | 7.1 |
| (**2**) | $C_{23}H_{29}O_5FClN_3$ | 481.95 | 183–185 | 4.09 | 7.0 |
| (**3**) | $C_{24}H_{31}O_5FClN_3$ | 495.98 | 170–171 | 4.42 | 7.3 |
| (**4**) | $C_{25}H_{33}O_5FClN_3$ | 510.00 | 180–182 | 4.54 | 7.4 |
| (**5**) | $C_{22}H_{27}O_5FClN_3$ | 467.92 | 188–190 | 4.23 | 7.5 |
| (**6**) | $C_{23}H_{29}O_5FClN_3$ | 481.95 | 181–183 | 4.55 | 7.6 |
| (**7**) | $C_{24}H_{31}O_5FClN_3$ | 495.98 | 167–169 | 4.88 | 7.4 |
| (**8**) | $C_{25}H_{33}O_5FClN_3$ | 510.00 | 162–164 | 5.07 | 7.3 |
| (**9**) | $C_{23}H_{30}O_6ClN_3$ | 479.96 | 197–199 | 2.33 | 7.9 |
| (**10**) | $C_{24}H_{32}O_6ClN_3$ | 493.99 | 199–201 | 2.31 | 7.5 |
| (**11**) | $C_{25}H_{34}O_6ClN_3$ | 508.01 | 196–198 | 2.57 | 7.8 |
| (**12**) | $C_{26}H_{36}O_6ClN_3$ | 522.04 | 184–186 | 2.91 | 7.9 |

[1] Cmpd. = compound, [2] *MW* = molecular weight (expressed in g/mol units), [3] mp = melting point values (in °C), [4] log *P* = decadic logarithm of a partition coefficient (*P*) estimated for an octan-1-ol/buffer (p*H* = 7.0) partition system, [5] p$K_a$ = acid-base dissociation constant, [6] *nd* = not determined (compound was insufficiently dissolved in an aqueous environment).

**Table S3.** The Simplified Molecular Input Line Entry System (SMILES) codes of the compounds (**Ib**)–(**IVb**), (**1b**)–(**12b**), (**13**), (**14**) and bedaquiline (BDQ; **17**). The codes were generated via the SwissADME interactive tool [10].

| [1] Cmpd. | SMILES Code |
|---|---|
| (**Ib**) | C1C(NC(=O)OC)=CC=C(C(=O)OCC(O)CN2CCN(C3C=CC=CC=3)CC2)C=1 |
| (**IIb**) | C1C(NC(=O)OCC)=CC=C(C(=O)OCC(O)CN2CCN(C3C=CC=CC=3)CC2)C=1 |
| (**IIIb**) | C1C(NC(=O)OCCC)=CC=C(C(=O)OCC(O)CN2CCN(C3C=CC=CC=3)CC2)C=1 |
| (**IVb**) | C1C(NC(=O)OCCCC)=CC=C(C(=O)OCC(O)CN2CCN(C3C=CC=CC=3)CC2)C=1 |
| (**1b**) | C1C(NC(=O)OC)=CC=C(C(=O)OCC(O)CN2CCN(C3C=CC(F)=CC=3)CC2)C=1 |
| (**2b**) | C1C(NC(=O)OCC)=CC=C(C(=O)OCC(O)CN2CCN(C3C=CC(F)=CC=3)CC2)C=1 |
| (**3b**) | C1C(NC(=O)OCCC)=CC=C(C(=O)OCC(O)CN2CCN(C3C=CC(F)=CC=3)CC2)C=1 |
| (**4b**) | C1C(NC(=O)OCCCC)=CC=C(C(=O)OCC(O)CN2CCN(C3C=CC(F)=CC=3)CC2)C=1 |
| (**5b**) | C1C(NC(=O)OC)=CC=C(C(=O)OCC(O)CN2CCN(C3C(F)=CC=CC=3)CC2)C=1 |
| (**6b**) | C1C(NC(=O)OCC)=CC=C(C(=O)OCC(O)CN2CCN(C3C(F)=CC=CC=3)CC2)C=1 |
| (**7b**) | C1C(NC(=O)OCCC)=CC=C(C(=O)OCC(O)CN2CCN(C3C(F)=CC=CC=3)CC2)C=1 |
| (**8b**) | C1C(NC(=O)OCCCC)=CC=C(C(=O)OCC(O)CN2CCN(C3C(F)=CC=CC=3)CC2)C=1 |
| (**9b**) | C1C(NC(=O)OC)=CC=C(C(=O)OCC(O)CN2CCN(C3C(OC)=CC=CC=3)CC2)C=1 |
| (**10b**) | C1C(NC(=O)OCC)=CC=C(C(=O)OCC(O)CN2CCN(C3C(OC)=CC=CC=3)CC2)C=1 |
| (**11b**) | C1C(NC(=O)OCCC)=CC=C(C(=O)OCC(O)CN2CCN(C3C(OC)=CC=CC=3)CC2)C=1 |
| (**12b**) | C1C(NC(=O)OCCCC)=CC=C(C(=O)OCC(O)CN2CCN(C3C(OC)=CC=CC=3)CC2)C=1 |
| [2] (**13**) | C1C=CC(C(OCC(O)CN2CCN(C3C(OC)=CC=CC=3)CC2)C2C=CC=CC=2)=C2C=CC=CC=12 |
| [2] (**14**) | COC1=CC(=CC=C1)N1CCN(CC(O)COC(C2=CC=CC=C2)C2=CC=CC3=C2C=CC=C3)CC1 |
| BDQ (**17**) | CN(C)CC[C@@](C1=CC=CC2=CC=CC=C21)([C@H](C3=CC=CC=C3)C4=C(N=C5C=CC(=CC5=C4)Br)OC)O |

[1] Cmpd. = compound, [2] (**13**), (**14**) = both compounds were synthesized and screened in vitro within the research [7].

**Table S4.** The prediction of phase I and phase II metabolites, that could be formed in humans, for the compounds (**Ib**)–(**IVb**) and (**1b**)–(**12b**) through the web--based GLORYx applet [30]. The priority score value, theoretically found within an interval of 0.00–1.00, was listed for selected metabolic pathways related to individual molecules.

| [1] Cmpd. | *O*-Glucuronidation / *O*-sulfation (of an OH-group) | *N*-Dealkylation (opening of a piperazine ring) | Oxidative hydroxylation (of a distal phenyl ring) | *N*-oxidation (of a $N^4$-atom) | Hydrolysis (of an ester portion within a carbamate moiety) | Hydrolysis (of a carbamate moiety) | *O*-Desmethylation (within a 2′-$OCH_3$ moiety attached to a phenyl ring) |
|---|---|---|---|---|---|---|---|
| (**Ib**) | 0.60 / 0.36 | 0.51 | 0.51 (for both 2′- and 4′-positions) | 0.51 | 0.50 | 0.30 | [2] *np* |
| (**IIb**) | 0.59 / 0.36 | 0.46 | 0.46 (for both 2′- and 4′-positions) | 0.46 | 0.36 | 0.20 | *np* |
| (**IIIb**) | 0.60 / 0.36 | 0.43 | 0.43 (for both 2′- and 4′-positions) | 0.43 | 0.31 | 0.16 | *np* |
| (**IVb**) | 0.59 / 0.39 | 0.41 | 0.41 (for both 2′- and 4′-positions) | 0.41 | 0.26 | 0.12 | *np* |
| (**1b**) | 0.60 / 0.35 | 0.48 | 0.48 (for 2′-position) | 0.48 | 0.50 | 0.30 | *np* |
| (**2b**) | 0.59 / 0.36 | 0.44 | 0.44 (for 2′-position) | 0.44 | 0.36 | 0.20 | *np* |
| (**3b**) | 0.60 / 0.36 | 0.41 | 0.41 (for 2′-position) | 0.41 | 0.31 | 0.16 | *np* |
| (**4b**) | 0.59 / 0.36 | 0.39 | 0.39 (for 2′-position) | 0.39 | 0.26 | 0.12 | *np* |
| (**5b**) | 0.60 / 0.35 | 0.44 | 0.44 (for both 4′- and 6′-positions) | 0.44 | 0.50 | 0.30 | *np* |
| (**6b**) | 0.59 / 0.36 | 0.38 | 0.38 (for both 4′- and 6′-positions) | 0.38 | 0.36 | 0.20 | *np* |
| (**7b**) | 0.60 / 0.36 | 0.37 | 0.37 (for both 4′- and 6′-positions) | 0.37 | 0.31 | 0.16 | *np* |
| (**8b**) | 0.59 / 0.39 | 0.35 | 0.35 (for both 4′- and 6′-positions) | 0.35 | 0.26 | 0.12 | *np* |

[1] Cmpd. = compound, [2] *np* = considered group (2′-$OCH_3$-moiety) was not present in a structure of a compound.

**Table S4.** *Continuation*

| [1] Cmpd. | *O*-Glucuronidation / *O*-sulfation (of an OH-group) | *N*-Dealkylation (opening of a piperazine ring) | Oxidative hydroxylation (of a distal phenyl ring) | *N*-oxidation (of a $N^4$-atom) | Hydrolysis (of an ester portion within a carbamate moiety) | Hydrolysis (of a carbamate moiety) | *O*-Demethylation (within a 2′-$OCH_3$ moiety attached to a phenyl ring) |
|---|---|---|---|---|---|---|---|
| (**9b**) | 0.60 / 0.35 | 0.24 | 0.30 (for both 4′- and 6′-positions) | 0.27 | 0.50 | 0.30 | 0.36 |
| (**10b**) | 0.59 / 0.36 | 0.21 | 0.27 (for both 4′- and 6′-positions) | 0.27 | 0.36 | 0.20 | 0.33 |
| (**11b**) | 0.60 / 0.36 | 0.21 | 0.24 (for both 4′- and 6′-positions) | 0.24 | 0.31 | 0.16 | 0.30 |
| (**12b**) | 0.59 / 0.39 | 0.19 | 0.22 (for both 4′- and 6′-positions) | 0.22 | 0.26 | 0.12 | 0.32 |

[1] Cmpd. = compound, [2] *np* = considered group (2′-$OCH_3$-moiety) was not present in a structure of a compound.

**Table S5.** The readouts from a chemometric principal component analysis (PCA) method exploring the relationships between structural, physicochemical and pharmacokinetic properties of analyzed compounds (**Ib**)–(**IVb**) and (**1b**)–(**12b**) through the XLSTAT ver. 2019 2.2 statistical software add-in (Addinsoft, New York, NY, USA) for Microsoft Excel (Microsoft 365; Microsoft Corp., Redmont, WA, USA). The relationships are described by positive or negative values of Pearson's correlation coefficient (*r*).

| Variables | [1] *MW* | [2] F*sp*³ | [3] *TPSA* (Å²) | [4] *MolFlex* | [5] *MR* (m³/mol) | [6] *MolVol* (Å³) | [7] log $P_{av}$ | [8] IA (%) | [9] log $P_{app}$ | [10] log BB | [11] log PS | [12] *QED* |
|---|---|---|---|---|---|---|---|---|---|---|---|---|
| ***MW*** | 1 | 0.912 | 0.969 | –0.221 | 0.921 | 0.953 | 0.800 | –0.430 | –0.611 | –0.771 | –0.227 | –0.889 |
| **F*sp*³** | 0.912 | 1 | 0.977 | –0.037 | 0.986 | 0.970 | 0.825 | –0.371 | –0.773 | –0.457 | 0.101 | –0.910 |
| ***TPSA* (Å²)** | 0.969 | 0.977 | 1 | –0.152 | 0.988 | 0.996 | 0.792 | –0.466 | –0.677 | –0.593 | –0.043 | –0.926 |
| ***MolFlex*** | –0.221 | –0.037 | –0.152 | 1 | –0.111 | –0.184 | 0.207 | 0.572 | –0.465 | 0.390 | 0.311 | 0.027 |
| ***MR* (m³/mol)** | 0.921 | 0.986 | 0.988 | –0.111 | 1 | 0.992 | 0.757 | –0.482 | –0.692 | –0.465 | 0.069 | –0.919 |
| ***MolVol* (Å³)** | 0.953 | 0.970 | 0.996 | –0.184 | 0.992 | 1 | 0.736 | –0.537 | –0.643 | –0.557 | –0.029 | –0.909 |
| **log $P_{av}$** | 0.800 | 0.825 | 0.792 | 0.207 | 0.757 | 0.736 | 1 | 0.163 | –0.841 | –0.520 | 0.080 | –0.842 |
| **IA (%)** | –0.430 | –0.371 | –0.466 | 0.572 | –0.482 | –0.537 | 0.163 | 1 | –0.120 | 0.284 | 0.253 | 0.259 |
| **log $P_{app}$** | –0.611 | –0.773 | –0.677 | –0.465 | –0.692 | –0.643 | –0.841 | –0.120 | 1 | 0.207 | –0.274 | 0.627 |
| **log BB** | –0.771 | –0.457 | –0.593 | 0.390 | –0.465 | –0.557 | –0.520 | 0.284 | 0.207 | 1 | 0.666 | 0.494 |
| **log PS** | –0.227 | 0.101 | –0.043 | 0.311 | 0.069 | –0.029 | 0.080 | 0.253 | –0.274 | 0.666 | 1 | –0.073 |
| ***QED*** | –0.889 | –0.910 | –0.926 | 0.027 | –0.919 | –0.909 | –0.842 | 0.259 | 0.627 | 0.494 | –0.073 | 1 |

[1] *MW* = molecular weight (in g/mol units), [2] F*sp*³ = fraction of *sp*³-hybridized *C*-atoms, that is, number of *sp*³-hybridized *C*-atoms/total *C*-atoms count, [3] *TSA* = total surface area (in Å² units), [4] *MolFlex* = molecular flexibility, [5] *MR* = molar refractivity (in m³/mol units), [6] *MolVol* = molecular volume (in Å³ units) generated via the Molsoft L.L.C. applet [14], [7] log $P_{av}$ = average value of a decadic logarithm of a partition coefficient (*P*) value calculated from log *P* outputs of particular predictive methods (Table 2 in the main text) for an octan-1-ol/water partition system, [8] IA (%) = intestinal absorption in humans (in percentages), [9] log $P_{app}$ = decadic logarithm of an apparent permeability coefficient ($P_{app}$), [10] log BB = logarithmic value of blood-to-brain ratio [11], [11] log PS = blood–brain barrier permeability-surface area product [11], [12] *QED* = quantitative estimate of drug-likeness [32].

**Table S6.** The MM-GBSA energies (MM-GBSA ΔG Bind parameter; in kcal/mol units) calculated for particular (*R*)- and (*S*)-enantiomeric forms of non-protonated compounds (**Ib**)–(**IVb**) and (**1b**)–(**12b**) that were docked into ATP synthase.

| [1] **Cmpd.** | **MM-GBSA ΔG Bind (kcal/mol)** | **Cmpd.** | **MM-GBSA ΔG Bind (kcal/mol)** |
|---|---|---|---|
| (*R*)-(**Ib**) | –43.83 | (*R*)-(**5b**) | –37.51 |
| (*S*)-(**Ib**) | –42.73 | (*S*)-(**5b**) | –38.24 |
| (*R*)-(**IIb**) | –47.66 | (*R*)-(**6b**) | –53.61 |
| [2] (*S*)-(**IIb**) | –36.37 | (*S*)-(**6b**) | –37.07 |
| (*R*)-(**IIIb**) | –56.43 | (*R*)-(**7b**) | –48.45 |
| (*S*)-(**IIIb**) | –37.24 | (*S*)-(**7b**) | –41.94 |
| (*R*)-(**IVb**) | –46.67 | (*R*)-(**8b**) | –59.42 |
| (*S*)-(**IVb**) | –51.20 | (*S*)-(**8b**) | –57.19 |
| (*R*)-(**1b**) | –43.18 | (*R*)-(**9b**) | –59.79 |
| (*S*)-(**1b**) | –48.39 | [3] (*S*)-(**9b**) | –61.96 |
| (*R*)-(**2b**) | –55.65 | (*R*)-(**10b**) | –56.08 |
| (*S*)-(**2b**) | –61.40 | (*S*)-(**10b**) | –37.96 |
| (*R*)-(**3b**) | –58.09 | (*R*)-(**11b**) | –60.79 |
| (*S*)-(**3b**) | –44.62 | (*S*)-(**11b**) | –39.09 |
| (*R*)-(**4b**) | –47.71 | (*R*)-(**12b**) | –56.22 |
| (*S*)-(**4b**) | –44.07 | (*S*)-(**12b**) | –52.55 |

[1] Cmpd. = compound, [2] (*S*)-(**IIb**) = indication by a light orange colour meant that the compound (*S*)-(**IIb**) had the highest MM-GBSA energy from those related to other presently investigated enantiomers of particular non-protonated derivatives, [3] (*S*)-(**9b**) = indication by a light green colour meant that the compound (*S*)-(**9b**) had the lowest MM-GBSA energy from those related to other presently investigated enantiomers of particular non-protonated derivatives.

**Table S7.** The MM-GBSA energies (MM-GBSA ΔG Bind parameter; in kcal/mol units) calculated for particular (*R*)- and (*S*)-enantiomeric forms of in vitro screened salts (**I**)–(**IV**) and (**1**)–(**12**) that were docked into ATP synthase.

| [1] **Cmpd.** | **MM-GBSA ΔG Bind (kcal/mol)** | **Cmpd.** | **MM-GBSA ΔG Bind (kcal/mol)** |
|---|---|---|---|
| (***R***)-(**I**) | –63.60 | (***R***)-(**5**) | –67.01 |
| (*S*)-(**I**) | –59.63 | (*S*)-(**5**) | –65.37 |
| (***R***)-(**II**) | –66.19 | (***R***)-(**6**) | –62.13 |
| (*S*)-(**II**) | –62.73 | (*S*)-(**6**) | –64.40 |
| (***R***)-(**III**) | –68.12 | [2] (***R***)-(**7**) | –70.09 |
| (*S*)-(**III**) | –61.21 | (*S*)-(**7**) | –60.28 |
| (***R***)-(**IV**) | –67.04 | (***R***)-(**8**) | –66.61 |
| (*S*)-(**IV**) | –61.65 | (*S*)-(**8**) | –69.78 |
| (***R***)-(**1**) | –57.85 | (***R***)-(**9**) | –62.59 |
| (*S*)-(**1**) | –51.65 | [3] (*S*)-(**9**) | –42.28 |
| (***R***)-(**2**) | –64.51 | (***R***)-(**10**) | –68.58 |
| (*S*)-(**2**) | –64.56 | (*S*)-(**10**) | –61.05 |
| (***R***)-(**3**) | –68.42 | (***R***)-(**11**) | –61.28 |
| (*S*)-(**3**) | –59.32 | (*S*)-(**11**) | –67.19 |
| (***R***)-(**4**) | –65.53 | (***R***)-(**12**) | –68.90 |
| (*S*)-(**4**) | –62.07 | (*S*)-(**12**) | –61.50 |

[1] Cmpd. = compound, [2] (*R*)-(**7**) = indication by a light green colour meant that the compound (***R***)-(**7**) had the lowest MM-GBSA energy from those related to other presently investigated enantiomers of particular salts, [3] (*S*)-(**9**) = indication by a light orange colour meant that the compound (*S*)-(**9**) had the highest MM-GBSA energy from those related to other presently investigated enantiomers of particular salts.

**Table S8.** The ligand docking score values (in kcal/mol units) calculated for particular (*R*)- and (*S*)-enantiomeric forms of non-protonated compounds (**Ib**)–(**IVb**) and (**1b**)–(**12b**) that were docked into ATP synthase.

| [1] **Cmpd.** | **Docking score (kcal/mol)** | **Cmpd.** | **Docking score (kcal/mol)** |
|---|---|---|---|
| (*R*)-(**Ib**) | –2.143 | (*R*)-(**5b**) | –1.412 |
| (*S*)-(**Ib**) | –1.696 | (*S*)-(**5b**) | –1.098 |
| (*R*)-(**IIb**) | –2.242 | (*R*)-(**6b**) | –2.597 |
| [2] (*S*)-(**IIb**) | –0.906 | (*S*)-(**6b**) | –1.425 |
| (*R*)-(**IIIb**) | –2.315 | (*R*)-(**7b**) | –2.178 |
| (*S*)-(**IIIb**) | –1.903 | (*S*)-(**7b**) | –1.491 |
| (*R*)-(**IVb**) | –2.160 | (*R*)-(**8b**) | –1.646 |
| (*S*)-(**IVb**) | –1.171 | (*S*)-(**8b**) | –1.719 |
| (*R*)-(**1b**) | –2.593 | (*R*)-(**9b**) | –1.696 |
| (*S*)-(**1b**) | –2.700 | (*S*)-(**9b**) | –1.264 |
| (*R*)-(**2b**) | –2.685 | (*R*)-(**10b**) | –2.853 |
| (*S*)-(**2b**) | –1.548 | (*S*)-(**10b**) | –1.346 |
| (*R*)-(**3b**) | –2.552 | (*R*)-(**11b**) | –2.932 |
| (*S*)-(**3b**) | –1.845 | (*S*)-(**11b**) | –2.261 |
| (*R*)-(**4b**) | –2.779 | (*R*)-(**12b**) | –1.779 |
| (*S*)-(**4b**) | –2.227 | [3] (*S*)-(**12b**) | –2.999 |

[1] Cmpd. = compound, [2] (*S*)-(**IIb**) = indication by a light orange colour meant that the compound (*S*)-(**IIb**) had the highest docking score value from those related to other presently investigated enantiomers of particular non-protonated derivatives, [3] (*S*)-(**12b**) = indication by a light green colour meant that the compound (*S*)-(**12b**) had the lowest docking score value from those related to other presently investigated enantiomers of particular non-protonated derivatives.

**Table S9.** The ligand docking score values (in kcal/mol units) calculated for particular (*R*)- and (*S*)-enantiomeric forms of in vitro screened salts (**I**)–(**IV**) and (**1**)–(**12**) that were docked into ATP synthase.

| [1] Cmpd. | Docking score (kcal/mol) | Cmpd. | Docking score (kcal/mol) |
|---|---|---|---|
| (*R*)-(**I**) | –5.666 | (*R*)-(**5**) | –6.135 |
| (*S*)-(**I**) | –5.777 | (*S*)-(**5**) | –5.769 |
| (*R*)-(**II**) | –5.852 | (*R*)-(**6**) | –6.192 |
| (*S*)-(**II**) | –5.630 | (*S*)-(**6**) | –5.884 |
| (*R*)-(**III**) | –5.681 | (*R*)-(**7**) | –5.989 |
| (*S*)-(**III**) | –5.495 | (*S*)-(**7**) | –5.554 |
| (*R*)-(**IV**) | –5.557 | (*R*)-(**8**) | –5.832 |
| (*S*)-(**IV**) | –5.570 | (*S*)-(**8**) | –5.309 |
| (*R*)-(**1**) | –6.006 | (*R*)-(**9**) | –5.287 |
| (*S*)-(**1**) | –5.663 | [3] (*S*)-(**9**) | –4.377 |
| [2] (*R*)-(**2**) | –6.306 | (*R*)-(**10**) | –5.855 |
| (*S*)-(**2**) | –6.014 | (*S*)-(**10**) | –5.656 |
| (*R*)-(**3**) | –5.863 | (*R*)-(**11**) | –5.053 |
| (*S*)-(**3**) | –5.581 | (*S*)-(**11**) | –5.305 |
| (*R*)-(**4**) | –5.693 | (*R*)-(**12**) | –5.216 |
| (*S*)-(**4**) | –5.464 | (*S*)-(**12**) | –4.760 |

[1] Cmpd. = compound, [2] (*R*)-(**2**) = indication by a light green colour meant that the compound (*R*)-(**2**) had the lowest docking score value from those related to other presently investigated enantiomers of particular salts, [3] (*S*)-(**9**) = indication by a light orange colour meant that the compound (*S*)-(**9**) had the highest docking score value from those related to other presently investigated enantiomers of particular salts.

**Table S10.** The interactions between substituents (atoms / group) attached to a distal phenyl ring of the compounds (**I**)–(**IV**) and (**1**)–(**12**) in the position 2′ and/or 4′, and particular amino acids (AAs) present in an active site of ATP synthase.

| [1] **Cmpds.** | **Substituent** | **Type of interaction** | **Interacting AAs** |
|---|---|---|---|
| (**I**)–(**IV**) | 2′-H, 4′-H (interaction through a phenyl ring) | hydrophobic | [2] Phe 2:53, Phe 2:54, [3] Ile 2:55 |
| (**1**)–(**4**) | 4′-F | hydrophobic | Phe 2:54, [4] Thr 2:51, [5] Pro 2:52 |
| | | hydrogen bond | [6] Gln *a*:241 |
| (**5**)–(**8**) | 2′-F | ionic | [7] Arg 2:48 |
| | | hydrophobic | Phe 2:50, Phe 2:54 |
| | | hydrogen bond | Thr 2:51, Gln *a*:241 |
| (**9**)–(**12**) | 2′-OCH3 | hydrophobic | [8] Val *a*:75, Phe 2:54, Phe 2:50, Gln *a*:74 |
| | | hydrogen bond | Gln *a*:74, [9] Asn *a*:73, Gln *a*: 241, [10] Gly 2:47 |
| | | Coulombic | [11] Glu *a*:244 |

[1] Cmpds. = compounds (their sets), [2] Phe = phenylalanine, [3] Ile = isoleucine, [4] Thr = threonine, [5] Pro = proline, [6] Gln = glutamine, [7] Arg = arginine, [8] Val = valine, [9] Asn = asparagine, [9] Gly = glycine, [11] Glu = glutamic acid. The *a* letter in the labelling of particular amino acid (AA) residues indicated the *a* subunit in a structure of ATP synthase, the number 2 in the labelling of particular AA residues indicated an $\alpha$-helix no. 2 within the *c* subunit of ATP synthase.

**Table S11.** The individual stereoisomers of the ligands (**IV**) and (**12**), particular groups in their structure interacting with individual amino acids (AAs) present in an active site of ATP synthase and interaction lengths (in Å units).

| [1] Cmpd. | Interacting group | Interacting AAs | Interaction lengths (Å) |
|---|---|---|---|
| (*R*)-(**IVb**) | OH | [2] Ser *a*:77 | 1.76 |
| | distal phenyl | Ser *a*:77 | 2.33, 2.34 |
| | NH | [3] Pro 2:43 | 1.88 |
| (*S*)-(**IVb**) | OH | Ser *a*:77 | 1.93, 2.36 |
| | OH | [4] Gly 2:47 | 1.83 |
| | OH | [5] Glu 2:44 | 1.96 |
| (*R*)-(**IV**) | $NH^+$ | [6] Ala *a*:78 | 1.99 |
| | OH | Ser *a*:77 | 1.72 |
| | NH | Pro 2:43 | 1.93 |
| | phenyl | Ser *a*:77 | 2.39 |
| | distal phenyl | [7] Gln *a*:241 | 2.40, 2.00 |
| (*S*)-(**IV**) | $NH^+$ | Ala *a*:78 | 1.83 |
| | NH | Pro 2:43 | 1.86 |
| | phenyl | Gly 2:47 | 2.39 |
| | distal phenyl ($\pi$–$\pi$) | Phe 2:54 | 2.57 |
| (*R*)-(**12b**) | OH | [8] Leu *a*:245 | 2.11 |
| | distal phenyl | [9] Thr 2:51 | 2.40, 2.27 |
| | CO group | Ser *a*:77 | 1.72 |
| (*S*)-(**12b**) | distal phenyl ($\pi$–$\pi$) | [10] Phe *a*:229 | 4.31 |
| | distal phenyl ($\pi$–$\pi$) | Phe *a*:54 | 5.01 |
| (*R*)-(**12**) | $NH^+$ | Ala *a*:78 | 1.98 |
| | OH | Ser *a*:77 | 1.72 |
| | CO | Ser *a*:77 | 1.88 |
| | NH | Pro 2:43 | 1.88 |
| | $OCH_3$ | Glu *a*:244 | 2.42 |
| (*S*)-(**12**) | OH | Ser *a*:77 | 1.72 |
| | $NH^+$ | Ala *a*:78 | 1.92 |
| | NH | Pro 2:43 | 2.05 |
| | $OCH_3$ | Glu *a*:244 | 2.81 |

[1] Cmpd. = compound, [2] Ser = serine, [3] Pro = proline, [4] Gly = glycine, [5] Glu = glutamic acid, [6] Ala = alanine, [7] Gln = glutamine, [8] Leu = leucine, [9] Thr = threonine, [10] Phe = phenylalanine. The term 'distal phenyl' represented a phenyl moiety within a 4-(substituted phenyl)piperazin-1-yl fragment. The *a* letter in the labelling of particular amino acid (AA) residues indicated the *a* subunit in a structure of ATP synthase, the number 2 in the labelling of particular AA residues indicated an $\alpha$-helix no. 2 within the *c* subunit of ATP synthase.

***Note to Table S11***

In Table S10, only the most notable interactions between particular groups present in a structure of individual stereoisomeric forms of the compounds (**IV**) and (**12**), and individual AA residues present in an active site of ATP synthase were provided.

**Table S12.** The prediction of [1] score for the compounds (**Ib**)–(**IVb**) and (**1b**)–(**12b**), describing their theoretical ability to inhibit growth of selected mycobacterial strains, namely, *Mycobacterium mageritense*, avirulent *M. tuberculosis* $H_{37}R_a$ (*Mtb* $H_{37}R_a$), *M. bovis* BCG, resistant *M. tuberculosis* $H_{37}R_v$ (*Mtb* $H_{37}R_{v-r}$), resistant *M. ulcerans* (*M. ulcerans*$_r$), resistant *M. avium* (*M. avium*$_r$) and *M. phlei*, using the web-based Antibac-Pred applet [57].

| [2] **Cmpd.** | ***M. mageritense*** | ***Mtb* $H_{37}R_a$** | ***M. bovis* BCG** | ***Mtb* $H_{37}R_{v-r}$** | ***M. ulcerans*$_r$** | ***M. avium*$_r$** | ***M. phlei*** |
|---|---|---|---|---|---|---|---|
| (**Ib**) | 0.2731 | 0.1288 | 0.1219 | < 0.1000 | < 0.1000 | < 0.1000 | < 0.1000 |
| (**IIb**) | 0.2119 | 0.1979 | 0.1776 | 0.1008 | < 0.1000 | < 0.1000 | < 0.1000 |
| (**IIIb**) | 0.2246 | 0.1568 | 0.1363 | < 0.1000 | < 0.1000 | < 0.1000 | < 0.1000 |
| (**IVb**) | 0.2078 | 0.2063 | 0.1814 | < 0.1000 | < 0.1000 | < 0.1000 | < 0.1000 |
| (**1b**) | 0.1898 | 0.1728 | < 0.1000 | < 0.1000 | < 0.1000 | < 0.1000 | < 0.1000 |
| (**2b**) | 0.1135 | 0.2392 | 0.1462 | 0.1071 | < 0.1000 | < 0.1000 | < 0.1000 |
| (**3b**) | 0.1354 | 0.2026 | 0.1058 | < 0.1000 | < 0.1000 | < 0.1000 | < 0.1000 |
| (**4b**) | 0.1163 | 0.2484 | 0.1522 | < 0.1000 | < 0.1000 | < 0.1000 | < 0.1000 |
| (**5b**) | 0.1879 | 0.1428 | < 0.1000 | < 0.1000 | 0.1255 | < 0.1000 | < 0.1000 |
| (**6b**) | 0.1132 | 0.2133 | 0.1363 | < 0.1000 | < 0.1000 | < 0.1000 | < 0.1000 |
| (**7b**) | 0.1345 | 0.1754 | < 0.1000 | < 0.1000 | < 0.1000 | < 0.1000 | < 0.1000 |
| (**8b**) | 0.1159 | 0.2216 | 0.1431 | < 0.1000 | < 0.1000 | < 0.1000 | < 0.1000 |
| (**9b**) | 0.2453 | < 0.1000 | < 0.1000 | < 0.1000 | < 0.1000 | < 0.1000 | < 0.1000 |
| (**10b**) | 0.2304 | 0.1187 | < 0.1000 | < 0.1000 | < 0.1000 | < 0.1000 | < 0.1000 |
| (**11b**) | 0.2429 | 0.1008 | < 0.1000 | < 0.1000 | < 0.1000 | < 0.1000 | < 0.1000 |
| (**12b**) | 0.2287 | 0.1241 | < 0.1000 | < 0.1000 | < 0.1000 | < 0.1000 | < 0.1000 |

[1] score = score parameter theoretically ranging from 0.0000 to 1.0000. The higher the score, the greater the likelihood that a derivative would efficiently act against particular mycobacterial species under conditions in vitro, [2] Cmpd. = compound.

**Table S13.** The readouts from a chemometric principal component analysis (PCA) method used to explore the relationships between structural, physicochemical and pharmacokinetic properties of non-protonated compounds (**IVb**), (**1b**)–(**4b**), (**7b**), (**8b**) and (**10b**)–(**12b**) through the XLSTAT ver. 2019 2.2 statistical software add-in (Addinsoft, New York, NY, USA) for Microsoft Excel (Microsoft 365; Microsoft Corp., Redmont, WA, USA). The relationships are described by positive or negative values of Pearson's correlation coefficient (*r*).

| Variables | [1] ***MW*** | [2] **F*sp*$^3$** | [3] ***TPSA*** **(Å$^2$)** | [4] ***MolFlex*** | [5] ***MR*** **(m$^3$/mol)** | [6] ***MolVol*** **(Å$^3$)** | [7] **log $P_{av}$** | [8] **IA (%)** | [9] **log $P_{app}$** | [10] **log BB** | [11] **log PS** | [12] ***QED*** |
|---|---|---|---|---|---|---|---|---|---|---|---|---|
| ***MW*** | 1 | 0.935 | 0.974 | –0.431 | 0.940 | 0.958 | 0.700 | –0.460 | –0.555 | –0.562 | 0.281 | –0.841 |
| **F*sp*$^3$** | 0.935 | 1 | 0.980 | –0.383 | 0.983 | 0.969 | 0.730 | –0.420 | –0.638 | –0.261 | 0.477 | –0.888 |
| ***TPSA*** **(Å$^2$)** | 0.974 | 0.980 | 1 | –0.434 | 0.993 | 0.995 | 0.672 | –0.512 | –0.557 | –0.364 | 0.392 | –0.884 |
| ***MolFlex*** | –0.431 | –0.383 | –0.434 | 1 | –0.433 | -0.475 | 0.099 | 0.688 | –0.254 | 0.306 | -0.023 | 0.170 |
| ***MR*** **(m$^3$/mol)** | 0.940 | 0.983 | 0.993 | –0.433 | 1 | 0.995 | 0.636 | –0.537 | –0.541 | –0.255 | 0.437 | –0.884 |
| ***MolVol*** **(Å$^3$)** | 0.958 | 0.969 | 0.995 | –0.475 | 0.995 | 1 | 0.601 | –0.587 | –0.507 | –0.331 | 0.384 | –0.857 |
| **log $P_{av}$** | 0.700 | 0.730 | 0.672 | 0.099 | 0.636 | 0.601 | 1 | 0.287 | –0.818 | –0.308 | 0.487 | –0.798 |
| **IA (%)** | –0.460 | –0.420 | –0.512 | 0.688 | –0.537 | –0.587 | 0.287 | 1 | –0.201 | 0.176 | 0.059 | 0.188 |
| **log $P_{app}$** | –0.555 | –0.638 | –0.557 | –0.254 | –0.541 | –0.507 | –0.818 | –0.201 | 1 | 0.156 | -0.488 | 0.523 |
| **log BB** | –0.562 | –0.261 | –0.364 | 0.306 | -0.255 | –0.331 | –0.308 | 0.176 | 0.156 | 1 | 0.352 | 0.175 |
| **log PS** | 0.281 | 0.477 | 0.392 | –0.023 | 0.437 | 0.384 | 0.487 | 0.059 | –0.488 | 0.352 | 1 | –0.524 |
| ***QED*** | –0.841 | –0.888 | –0.884 | 0.170 | –0.884 | –0.857 | –0.798 | 0.188 | 0.523 | 0.175 | –0.524 | 1 |

[1] *MW* = molecular weight (in g/mol units), [2] F*sp*$^3$ = fraction of *sp*$^3$-hybridized *C*-atoms, that is, number of *sp*$^3$-hybridized *C*-atoms/total *C*-atoms count, [3] *TSA* = total surface area (in Å$^2$ units), [4] *MolFlex* = molecular flexibility, [5] *MR* = molar refractivity (in m$^3$/mol units), [6] *MolVol* = molecular volume (in Å$^3$ units) generated via the Molsoft L.L.C. applet [14], [7] log $P_{av}$ = average value of a decadic logarithm of a partition coefficient (*P*) value calculated from log *P* outputs from particular predictive methods (Table 2 in the main text) for an octan-1-ol/water partition system, [8] IA (%) = intestinal absorption in humans (in percentages), [9] log $P_{app}$ = decadic logarithm of an apparent permeability coefficient ($P_{app}$), [10] log BB = logarithmic value of blood-to-brain ratio [11], [11] log PS = blood–brain barrier permeability-surface area product [11], [12] *QED* = quantitative estimate of drug-likeness [32].

**Table S14.** The MM-GBSA energies (MM-GBSA ΔG Bind parameter; in kcal/mol units) calculated for bedaquiline (BDQ; **17**) and its stereoisomeric forms that were docked into ATP synthase.

| [1] **Cmpd.** | **MM-GBSA ΔG Bind (kcal/mol)** |
|---|---|
| [2] (**1*R*,2*R***)-(**17**) | –34.45 |
| BDQ (**17**) with the (**1*R*,2*S***)-arrangement | –41.90 |
| (**1*S*,2*S***)-(**17**) | –35.54 |
| [3] (**1*S*,2*R***)-(**17**) | –46.21 |

[1] Cmpd. = compound, [2] (***1R*,*2R***)-(**17**) = indication by a light orange colour meant that the compound (***1R*,*2R***)-(**17**) had the highest MM-GBSA energy from from those related to other presently investigated stereoisomers of BDQ (**17**), [3] (***1S*,*2R***)-(**17**) = indication by a light green colour meant that the compound (***1S*,*2R***)-(**17**) had the lowest MM-GBSA energy from those related to other presently investigated stereoisomers of BDQ (**17**).

## B. NOTES

### *General Note*

All references, that were provided within a current Supplementary Materials document, were properly listed in the main text of a manuscript. The numbering of these references listed in brackets within the Supplementary Materials document corresponded to the numbering of the references listed in the main text (Section References).

### ***Synthesis of the Compounds (1)–(12) and (I)–(IV) – More Detailed Information***

*Reagents and Conditions – Extended Information*

(**i**) Particular commercially available alkyl chloroformates ($n$ = 0.10 mol), that is, methyl chloroformate (CAS: 79-22-1, $m$ = 9.45 g), ethyl chloroformate (CAS: 541-41-3, $m$ = 10.85 g), propyl chloroformate (CAS: 109-61-5, $m$ = 12.26 g) and butyl chloroformate (CAS: 592-34-7, $m$ = 13.66 g), were added drop-wise to a solution of 4-aminobenzoic acid (**1´**; CAS: 150-13-0, $n$ = 0.10 mol) and pyridine ($n$ = 0.1 mol) in acetone ($V$ = 100 mL). After the 1.5 h reflux, the cooled reaction mixture was evaporated to dryness. The residue of evaporation was dissolved in chloroform and a resulting by-product (pyridinium-chloride) of this reaction was removed by washing with distilled water. The organic phase was properly dried by anhydrous $MgSO_4$, evaporated and crude intermediates, that is, 4-[(alkoxycarbonyl)amino]benzoic acids (**2´a**)–(**2´d**) (alkoxy = methoxy to butoxy), were crystallized from ethanol 96%; (**ii**) particular alkyl 4-[(chlorocarbonyl)phenyl]carbamates (**3´a**)–(**3´d**) (alkyl = methyl to butyl) were obtained after the intermediates (**2´a**)–(**2´d**) were suspensed in toluene ($V$ = 120 mL), properly stirred and treated by gradually added thionyl chloride (CAS: 7719-09-7, $n$ = 0.20 mol). The particular mixtures were heated 2 h under reflux and isolated under reduced pressure. The crude products, particular acyl chlorides (**3´a**)–(**3´d**), were crystallized from toluene; (**iii**) (±)-(oxiran-2-yl)methyl 4-[(alkoxycarbonyl)amino]-benzoates (**4´a**)–(**4´d**) (alkoxy = methoxy to butoxy) were synthesized from the intermediates (**3´a**)–(**3´d**) ($n$ = 0.25 mol) that were dissolved in tetrahydrofuran (THF; $V$ = 250 mL) and cooled to –5 °C. These solutions were added dropwise at –5 °C to particular mixtures, consisting of a commercially available (±)-(oxiran-2-yl)methanol reagent (CAS: 556-52-5, $n$ = 0.25 mol) and triethylamine (CAS: 121-44-8, $n$ = 0.05 mol) dissolved in THF ($V$ = 50 mL). The resulting solutions were continuously stirred at laboratory temperature for 3 h, the precipitate that was formed was filtered and crude intermediates were isolated under reduced pressure. These crude compounds were crystallized from propan-2-ol. Reaction of (**3´a**)–(**3´d**) with (±)-(oxiran-2-yl)methanol was regioselective; (**iv**) individual mixtures, consisting of the intermediates (**4´a**)–(**4´d**) ($n$ = 0.25 mol) and commercially available basic amines ($n$ = 0.25 mol), that is, 1-(4-fluorophenyl)piperazine (CAS: 2252-63-3), 1-(2-fluorophenyl)piperazine (CAS: 1011-15-0), 1-(2--methoxyphenyl)piperazine (CAS: 35386-24-4) and 1-phenylpiperazine (CAS: 92-54-6), in propan-2-ol ($V$ = 150 mL) were heated for 4 h at 80 °C. The solvent was evaporated under reduced pressure and oily 3-[4-(4-fluoro-/2-fluoro-/2-methoxyphenyl)piperazin-1-yl]-2-hydroxypropyl 4-[(alkoxycarbonyl)amino]benzoates (**1b**)–(**12b**) and 2-hydroxy-3-(4-phenylpiperazin-1-yl)propyl 4-[(alkoxycarbonyl)amino]-benzoates (**Ib**)–(**IVb**) (alkoxy = methoxy to butoxy in both series) were isolated; (**v**) individual oily compounds (**1b**)–(**12b**) and (**Ib**)–(**IVb**) were dissolved in diethyl ether and such solutions of particular non-protonated compounds were converted to their chloride salts by the addition of an etheric solution of hydrochloric acid. The individual crude amine salts, that is, 1-(3-{4-[(alkoxycarbonyl)amino]-benzoyloxy}-2-hydroxypropyl)-4-(4-fluoro-/2-fluoro-/2-methoxyphenyl)piperazin-1-ium chlorides (**1**)–(**12**) and 1-(3-{4-[(alkoxycarbonyl)amino]benzoyloxy}-2-hydroxypropyl)-4-phenylpiperazin-1-ium chlorides (**I**)–(**IV**) (alkoxy = methoxy to butoxy in both series), were collected by filtration and recrystallized from particular alcohols to obtain white solids. The compounds (**1**)–(**5**), (**9**) and (**12**) were crystallized from ethanol 96%, the derivatives (**6**)–(**8**) and (**I**)–(**IV**) were crystallized from propan-2-ol, and the compounds (**10**) and (**11**) were crystallized from methanol.

*Spectral Characterization of the Compounds (**1**)–(**12**) and (**I**)–(**IV**)*

Equipment

The $^{1}H$ nuclear magnetic resonance ($^{1}H$ NMR) and $^{13}C$ nuclear magnetic resonance ($^{13}C$ NMR) spectra of the derivatives (**1**)–(**12**) and (**I**)–(**IV**) were recorded on the Gemini-2000 FT-NMR spectrometer (200 MHz for $^{1}H$ nucleus and 50 MHz for $^{13}C$ nucleus; Varian Inc., Palo Alto, CA, USA). The NMR spectra were measured at 25 °C and DMSO-$d_6$ was used as a solvent; the resulting spectra were referenced to its signals ($^{1}H$ NMR: $\delta_H$ = 2.5 ppm; $^{13}C$ NMR: $\delta_C$ = 39.5 ppm). Results were presented in the following format: chemical shift $\delta_H$ (ppm) / $\delta_C$ (ppm), multiplicity, coupling constant (*J*; in Hz units), number of protons and protons' position. Multiplicities were described by particular abbreviations: s (singlet), br s (broad singlet), d (doublet), t (triplet), q (quartet) and m (multiplet). Other abbreviation and indications that were used: pip (piperazine), Ar–F (data related to protons of an aromatic ring within a 4-fluoro-/2-fluorophenyl moiety) Ar–$OCH_3$ (data related to protons of an aromatic ring within a 2-methoxyphenyl moiety), Ar–$N_{pip}$ (data related to protons of a distal aromatic ring attached to a piperazin-1,4-diyl moiety).

The infrared (IR) spectra of the compounds (**1**)–(**12**) and (**I**)–(**IV**) were measured on the Nicolet Impact 410 FTIR spectrometer (Thermo Scientific, West Palm Beach, FL, USA) using a single-reflection attenuated total reflectance (ATR) technique using a zinc selenide crystal. The spectra were recorded by the accumulation of 256 scans in the wavenumber range of 4000–650 $cm^{-1}$ at a spectral resolution of 2 $cm^{-1}$. Abbreviations that were used: al (aliphatic), arom (aromatic), carb (carbonyl), es (ester).

Spectral data

*1-(2-Hydroxypropyl-3-{4-[(methoxycarbonyl)amino]benzoyloxy})-4-(4-fluorophenyl)piperazin-1-ium chloride (**1**).* $^{1}H$ NMR (200 MHz, DMSO-$d_6$), $\delta_H$ (ppm): 10.76 (s, 1H, $N\underline{H}^+$), 10.17 (s, 1H, N$\underline{H}$COO), 7.98 (d, *J* = 8.8 Hz, 2H, Ar–$\underline{H}$), 7.63 (d, *J* = 8.8 Hz, 2H, Ar–$\underline{H}$), 7.15–6.98 (m, 4H, Ar–F), 4.55–4.42 (m, 1H, C$\underline{H}$OH), 4.22 (d, *J* = 4.6 Hz, 2H, $COOC\underline{H}_2$), 3.76–3.09 (m, 10H, $\underline{H}_{pip}$ + $C\underline{H}_2N_{pip}$), 3.69 (s, 3H, $C\underline{H}_3$); $^{13}C$ NMR (50 MHz, DMSO-$d_6$), $\delta_C$ (ppm): 165.0, 156.5, 153.7, 146.3, 143.8, 130.5, 122.9, 117.7, 117.3, 115.4, 66.0, 63.3, 58.2, 52.0, 51.8, 50.8, 45.9, 45.8; FT-IR (ZnSe ATR, $cm^{-1}$): 3305 ($\nu$ N–H), 3270 ($\nu$ O–H), 2971 ($\nu$ C–H), 2566 ($\nu$ $NH^+$), 1723 ($\nu_{as}$ C=O), 1687 ($\nu$ NHC=O), 1595 ($\nu$ $C=C_{arom}$).

*1-(3-{4-[(Ethoxycarbonyl)amino]benzoyloxy}-2-hydroxypropyl)-4-(4-fluorophenyl)piperazin-1-ium chloride (**2**).* $^{1}H$ NMR (200 MHz, DMSO-$d_6$), $\delta_H$ (ppm): 10.79 (s, 1H, $N\underline{H}^+$), 10.11 (s, 1H, N$\underline{H}$COO), 7.98 (d, *J* = 8.8 Hz, 2H, Ar–$\underline{H}$), 7.63 (d, *J* = 8.8 Hz, 2H, Ar–$\underline{H}$), 7.15–6.98 (m, 4H, Ar–F), 6.06 (s, 1H, O$\underline{H}$), 4.56–4.42 (m, 1H, C$\underline{H}$OH), 4.22 (d, *J* = 4.9 Hz, 2H, $COOC\underline{H}_2$), 4.15 (q, *J* = 7.2 Hz, 2H, $C\underline{H}_2CH_3$), 3.80–3.09 (m, 10H, $H_{pip}$ + $C\underline{H}_2N_{pip}$), 1.25 (t, *J* = 7.1 Hz, 3H, $CH_2C\underline{H}_3$); $^{13}C$ NMR (50 MHz, DMSO-$d_6$), $\delta_C$ (ppm): 165.0, 156.5, 153.2, 146.3, 143.9, 130.5, 122.8, 117.7, 117.3, 115.3, 66.0, 63.3, 60.4, 58.2, 52.0, 50.9, 45.8, 14.3; FT-IR (ZnSe ATR, $cm^{-1}$): 3302 ($\nu$ N–H), 3266 ($\nu$ O–H), 2970 ($\nu$ C–H), 2563 ($\nu$ $NH^+$), 1718 ($\nu_{as}$ C=O), 1688 ($\nu$ NHC=O), 1592 ($\nu$ $C=C_{arom}$).

*1-(2-Hydroxypropyl-3-{4-[(propoxycarbonyl)amino]benzoyloxy})-4-(4-fluorophenyl)piperazin-1-ium chloride (**3**).* $^{1}H$ NMR (200 MHz, DMSO-$d_6$), $\delta_H$ (ppm): 10.82 (s, 1H, $N\underline{H}^+$), 10.09 (s, 1H, N$\underline{H}$COO), 7.97 (d, *J* = 8.8 Hz, 2H, Ar–$\underline{H}$), 7.63 (d, *J* = 8.8 Hz, 2H, Ar–$\underline{H}$), 7.15–6.98 m, 4H, Ar–F), 4.55–4.42 (m, 1H, C$\underline{H}$OH), 4.23 (d, *J* = 4.7 Hz, 2H, $COOC\underline{H}_2$), 4.06 (t, *J* = 6.7 Hz, 2H, $C\underline{H}_2CH_2CH_3$), 3.80–3.09 (m, 10H, $H_{pip}$ + $C\underline{H}_2N_{pip}$), 1.73–1.56 (m, 2H, $CH_2C\underline{H}_2CH_3$), 0.93 (t, *J* = 7.4 Hz, 3H, $CH_2CH_2C\underline{H}_3$); $^{13}C$ NMR (50 MHz, DMSO-$d_6$), $\delta_C$ (ppm): 165.1, 156.5, 153.1, 146.3, 143.9, 130.5, 122.8, 117.7, 117.3, 115.4, 65.9, 63.3, 58.2, 52.0, 50.8, 45.9, 45.8, 21.7, 10.1; FT-IR (ZnSe ATR, $cm^{-1}$): 3352 ($\nu$ N–H), 3270 ($\nu$ O–H), 2973 ($\nu$ C–H), 2568 ($\nu$ $NH^+$), 1721 ($\nu_{as}$ C=O), 1690 ($\nu$ NHC=O), 1591 ($\nu$ $C=C_{arom}$).

*1-(3-{4-[(Butoxycarbonyl)amino]benzoyloxy}-2-hydroxypropyl)-4-(4-fluorophenyl)piperazin-1-ium chloride (**4**).* $^{1}H$ NMR (200 MHz, DMSO-$d_6$), $\delta_H$ (ppm): 10.78 (s, 1H, $N\underline{H}^+$), 10.12 (s, 1H, N$\underline{H}$COO), 7.98 (d, *J* = 8.8 Hz, 2H, Ar–$\underline{H}$), 7.63 (d, *J* = 8.9 Hz, 2H, Ar–$\underline{H}$), 7.14–6.98 (m, 4H, Ar–F), 6.08 (s, 1H, O$\underline{H}$), 4.55–4.41 (m, 1H, C$\underline{H}$OH), 4.22 (d, *J* = 4.6 Hz, 2H, $COOC\underline{H}_2$), 4.10 (t, *J* = 6.5 Hz, 2H, $C\underline{H}_2CH_2CH_2CH_3$), 3.80–3.09 (m, 10H, $\underline{H}_{pip}$ + $C\underline{H}_2N_{pip}$), 1.67–1.54 (m, 2H, $CH_2C\underline{H}_2CH_2CH_3$), 1.46–1.28 (m, 2H, $CH_2CH_2C\underline{H}_2CH_3$), 0.90 (t, *J* = 7.2 Hz, 3H, $CH_2CH_2CH_2C\underline{H}_3$); $^{13}C$ NMR (50 MHz, DMSO-$d_6$), $\delta_C$ (ppm): 165.1, 156.5, 153.4, 146.3, 144.0,

130.6, 122.8, 117.7, 117.3, 115.3, 66.0, 64.2, 63.3, 58.2, 52.1, 50.9, 45.9, 30.4, 18.5, 13.5; FT-IR (ZnSe ATR, cm$^{-1}$): 3314 ($\nu$ N–H), 3264 ($\nu$ O–H), 2974 ($\nu$ C–H), 2565 ($\nu$ NH$^+$), 1720 ($\nu_{as}$ C=O), 1682 ($\nu$ NHC=O), 1598 ($\nu$ C=C$_{arom}$).

*1-(2-Hydroxypropyl-3-{4-[(methoxycarbonyl)amino]benzoyloxy})-4-(2-fluorophenyl)piperazin-1-ium chloride (**5**).* $^1$H NMR (200 MHz, DMSO-$d_6$), $\delta_H$ (ppm): 10.81 (s, 1H, N$\underline{H}^+$), 10.16 (s, 1H, N$\underline{H}$COO), 7.99 (d, $J$ = 8.7 Hz, 2H, Ar–$\underline{H}$), 7.63 (d, $J$ = 8.7 Hz, 2H, Ar–$\underline{H}$), 7.23–6.98 (m, 4H, Ar–F), 6.07 (s, 1H, O$\underline{H}$), 4.55–4.42 (m, 1H, C$\underline{H}$OH), 4.23 (d, $J$ = 4.8 Hz, 2H, COOC$\underline{H}_2$), 3.80–3.17 (m, 10H, $\underline{H}_{pip}$ + C$\underline{H}_2$N$_{pip}$), 3.69 (s, 3H, C$\underline{H}_3$); $^{13}$C NMR (50 MHz, DMSO-$d_6$), $\delta_C$ (ppm): 165.0, 154.8, 153.7, 143.8, 138.2, 130.5, 124.8, 123.2, 122.9, 119.5, 117.3, 116.0, 66.0, 63.3, 58.4, 52.3, 51.8, 51.1, 46.7; FT-IR (ZnSe ATR, cm$^{-1}$): 3239 ($\nu$ N–H), 3236 ($\nu$ O–H), 2969 ($\nu$ C–H), 2560 ($\nu$ NH$^+$), 1712 ($\nu_{as}$ C=O), 1681 ($\nu$ NHC=O), 1595 ($\nu$ C=C$_{arom}$).

*1-(3-{4-[(Ethoxycarbonyl)amino]benzoyloxy}-2-hydroxypropyl)-4-(2-fluorophenyl)piperazin-1-ium chloride (**6**).* $^1$H NMR (200 MHz, DMSO-$d_6$), $\delta_H$ (ppm): 10.82 (s, 1H, N$\underline{H}^+$), 10.11 (s, 1H, N$\underline{H}$COO), 7.98 (d, $J$ = 8.8 Hz, 2H, Ar–$\underline{H}$), 7.63 (d, $J$ = 8.8 Hz, 2H, Ar–$\underline{H}$), 7.23–6.98 (m, 4H, Ar–F), 6.07 (s, 1H, O$\underline{H}$), 4.55–4.42 (m, 1H, C$\underline{H}$OH), 4.23 (d, $J$ = 4.8 Hz, 2H, COOC$\underline{H}_2$), 4.15 (q, $J$ = 7.2 Hz, 2H, C$\underline{H}_2$CH$_3$), 3.80–3.15 (m, 10H, $\underline{H}_{pip}$ + C$\underline{H}_2$N$_{pip}$), 1.25 (t, $J$ = 7.1 Hz, 3H, CH$_2$C$\underline{H}_3$); $^{13}$C NMR (50 MHz, DMSO-$d_6$), $\delta_C$ (ppm): 165.1, 154.8, 153.2, 143.9, 138.2, 130.5, 124.8, 123.2, 122.8, 119.5, 117.3, 116.0, 66.0, 63.3, 60.4, 58.4, 52.3, 51.1, 46.7, 14.2; FT-IR (ZnSe ATR, cm$^{-1}$): 3318 ($\nu$ N–H), 3269 ($\nu$ O–H), 2975 ($\nu$ C–H), 2564 ($\nu$ NH$^+$), 1718 ($\nu_{as}$ C=O), 1686 ($\nu$ NHC=O), 1590 ($\nu$ C=C$_{arom}$).

*1-(2-Hydroxypropyl-3-{4-[(propoxycarbonyl)amino]benzoyloxy})-4-(2-fluorophenyl)piperazin-1-ium chloride (**7**).* $^1$H NMR (200 MHz, DMSO-$d_6$), $\delta_H$ (ppm): 10.87 (s, 1H, N$\underline{H}^+$), 10.13 (s, 1H, N$\underline{H}$COO), 7.98 (d, $J$ = 8.8 Hz, 2H, Ar–$\underline{H}$), 7.63 (d, $J$ = 8.8 Hz, 2H, Ar–$\underline{H}$), 7.23–6.98 (m, 4H, Ar–F), 6.08 (s, 1H, O$\underline{H}$), 4.55–4.42 (m, 1H, C$\underline{H}$OH), 4.22 (d, $J$ = 4.6 Hz, 2H, COOC$\underline{H}_2$), 4.06 (t, $J$ = 6.7 Hz, 2H, C$\underline{H}_2$CH$_2$CH$_3$), 3.80–3.15 (m, 10H, $\underline{H}_{pip}$ + C$\underline{H}_2$N$_{pip}$), 1.73–1.55 (m, 2H, CH$_2$C$\underline{H}_2$CH$_3$), 0.93 (t, $J$ = 7.4 Hz, 3H, CH$_2$CH$_2$C$\underline{H}_3$); $^{13}$C NMR (50 MHz, DMSO-$d_6$), $\delta_C$ (ppm): 165.0, 154.8, 153.3, 143.9, 138.2, 130.5, 124.8, 123.2, 122.8, 119.5, 117.3, 116.0, 65.9, 63.3, 58.4, 52.3, 51.1, 46.7, 21.7, 10.1; FT-IR (ZnSe ATR, cm$^{-1}$): 3326 ($\nu$ N–H), 3270 ($\nu$ O–H), 2970 ($\nu$ C–H), 2565 ($\nu$ NH$^+$), 1722 ($\nu_{as}$ C=O), 1688 ($\nu$ NHC=O), 1592 ($\nu$ C=C$_{arom}$).

*1-(3-{4-[(Butoxycarbonyl)amino]benzoyloxy}-2-hydroxypropyl)-4-(2-fluorophenyl)piperazin-1-ium chloride (**8**).* $^1$H NMR (200 MHz, DMSO-$d_6$), $\delta_H$ (ppm): 10.80 (s, 1H, N$\underline{H}^+$), 10.12 (s, 1H, N$\underline{H}$COO), 7.98 (d, $J$ = 8.8 Hz, 2H, Ar–$\underline{H}$), 7.63 (d, $J$ = 8.8 Hz, 2H, Ar–$\underline{H}$), 7.25–6.98 (m, 4H, Ar–F), 6.07 (s, 1H, O$\underline{H}$), 4.55–4.41 (m, 1H, C$\underline{H}$OH), 4.22 (d, $J$ = 4.6 Hz, 2H, COOC$\underline{H}_2$), 4.10 (t, $J$ = 6.5 Hz, 2H, C$\underline{H}_2$CH$_2$CH$_2$CH$_3$), 3.80–3.15 (m, 10H, $\underline{H}_{pip}$ + C$\underline{H}_2$N$_{pip}$), 1.68–1.54 (m, 2H, CH$_2$C$\underline{H}_2$CH$_2$CH$_3$), 1.47–1.28 (m, 2H, CH$_2$CH$_2$C$\underline{H}_2$CH$_3$), 0.91 (t, $J$ = 7.2 Hz, 3H, CH$_2$CH$_2$CH$_2$C$\underline{H}_3$); $^{13}$C NMR (50 MHz, DMSO-$d_6$), $\delta_C$ (ppm): 165.1, 154.8, 153.4, 143.9, 138.2, 130.6, 124.9, 123.3, 122.8, 119.5, 117.2, 116.0, 66.0, 64.1, 63.3, 58.3, 52.3, 51.0, 46.8, 30.4, 18.5, 13.5; FT-IR (ZnSe ATR, cm$^{-1}$): 3311 ($\nu$ N–H), 3262 ($\nu$ O–H), 2971 ($\nu$ C–H), 2561 ($\nu$ NH$^+$), 1720 ($\nu_{as}$ C=O), 1684 ($\nu$ NHC=O), 1591 ($\nu$ C=C$_{arom}$).

*1-(2-Hydroxypropyl-3-{4-[(methoxycarbonyl)amino]benzoyloxy})-4-(2-methoxyphenyl)piperazin-1-ium chloride (**9**).* $^1$H NMR (200 MHz, DMSO-$d_6$), $\delta_H$ (ppm): 10.73 (s, 1H, N$\underline{H}^+$), 10.17 (s, 1H, N$\underline{H}$COO), 7.99 (d, $J$ = 8.8 Hz, 2H, Ar–$\underline{H}$), 7.63 (d, $J$ = 8.9 Hz, 2H, Ar–$\underline{H}$), 7.06–6.85 (m, 4H, Ar–OCH$_3$), 6.05 (s, 1H, O$\underline{H}$), 4.55–4.41 (m, 1H, C$\underline{H}$OH), 4.22 (d, $J$ = 4.7 Hz, 2H, COOC$\underline{H}_2$), 3.82–3.04 (m, 10H, $\underline{H}_{pip}$ + C$\underline{H}_2$N$_{pip}$), 3.79 (s, 3H, OC$\underline{H}_3$), 3.69 (s, 3H, C$\underline{H}_3$); $^{13}$C NMR (50 MHz, DMSO-$d_6$), $\delta_C$ (ppm): 165.0, 153.7, 151.8, 143.8, 139.4, 130.5, 123.3, 122.9, 120.7, 118.1, 117.3, 112.0, 66.0, 63.3, 58.4, 55.3, 52.6, 51.8, 51.3, 46.7, 46.5; FT-IR (ZnSe ATR, cm$^{-1}$): 3232 ($\nu$ N–H), 3225 ($\nu$ O–H), 2963 ($\nu$ C–H), 2560 ($\nu$ NH$^+$), 1714 ($\nu_{as}$ C=O), 1682 ($\nu$ NHC=O), 1590 ($\nu$ C=C$_{arom}$).

*1-(3-{4-[(Ethoxycarbonyl)amino]benzoyloxy}-2-hydroxypropyl)-4-(2-methoxyphenyl)piperazin-1-ium chloride (**10**).* $^1$H NMR (200 MHz, DMSO-$d_6$), $\delta_H$ (ppm): 10.83 (s, 1H, N$\underline{H}^+$), 10.13 (s, 1H, N$\underline{H}$COO), 7.98 (d, $J$ = 8.7 Hz, 2H, Ar–$\underline{H}$), 7.62 (d, $J$ = 8.8 Hz, 2H, Ar–$\underline{H}$), 5.34 (s, 1H, O$\underline{H}$), 7.09–6.86 (m, 4H, Ar–OCH$_3$), 4.55–4.42 (m, 1H, C$\underline{H}$OH), 4.21 (d, $J$ = 5.6 Hz, 2H, COOC$\underline{H}_2$), 4.14 (q, $J$ = 7.2 Hz, 2H, C$\underline{H}_2$CH$_3$), 3.85–3.10 (m, 10H, $\underline{H}_{pip}$ + C$\underline{H}_2$N$_{pip}$), 3.79 (s, 3H, OC$\underline{H}_3$), 1.24 (t, $J$ = 7.1 Hz, 3H, CH$_2$C$\underline{H}_3$); $^{13}$C NMR (50 MHz, DMSO-$d_6$), $\delta_C$ (ppm): 165.1, 153.3, 151.8, 144.0, 139.0, 130.6, 123.7, 122.8, 120.8, 118.4, 117.3, 112.1, 66.0, 63.3, 60.5,

58.4, 55.4, 52.5, 51.3, 46.8, 46.7, 14.4; FT-IR (ZnSe ATR, $cm^{-1}$): 3257 ($\nu$ N–H), 3248 ($\nu$ O–H), 2970 ($\nu$ C–H), 2559 ($\nu$ $NH^+$), 1710 ($\nu_{as}$ C=O), 1681 ($\nu$ NHC=O), 1588 ($\nu$ $C{=}C_{arom}$).

*1-(2-Hydroxypropyl-3-{4-[(propoxycarbonyl)amino]benzoyloxy})-4-(2-methoxyphenyl)piperazin-1-ium chloride (**11**).* $^1$H NMR (200 MHz, DMSO-$d_6$), $\delta_H$ (ppm): 10.78 (s, 1H, $N\underline{H}^+$), 10.12 (s, 1H, $N\underline{H}COO$), 7.98 (d, $J$ = 8.8 Hz, 2H, Ar–$\underline{H}$), 7.63 (d, $J$ = 8.8 Hz, 2H, Ar–$\underline{H}$), 7.08–6.86 (m, 4H, Ar–$OCH_3$), 5.87 (s, 1H, $O\underline{H}$), 4.55–4.41 (m, 1H, $C\underline{H}OH$), 4.22 (d, $J$ = 4.7 Hz, 2H, $COOC\underline{H}_2$), 4.06 (t, $J$ = 6.7 Hz, 2H, $C\underline{H}_2CH_2CH_3$), 3.83–3.08 (m, 10H, $\underline{H}_{pip}$ + $C\underline{H}_2N_{pip}$), 3.79 (s, 3H, $OC\underline{H}_3$), 1.73–1.55 (m, 2H, $CH_2C\underline{H}_2CH_3$), 0.93 (t, $J$ = 7.4 Hz, 3H, $CH_2CH_2C\underline{H}_3$); $^{13}$C NMR (50 MHz, DMSO-$d_6$), $\delta_C$ (ppm): 165.1, 153.4, 151.8, 144.0, 139.1, 130.6, 123.5, 122.8, 120.8, 118.3, 117.3, 112.0, 66.0, 63.3, 58.4, 55.4, 52.5, 51.2, 46.8, 46.6, 21.7, 10.1; FT-IR (ZnSe ATR, $cm^{-1}$): 3310 ($\nu$ N–H), 3270 ($\nu$ O–H), 2974 ($\nu$ C–H), 2560 ($\nu$ $NH^+$), 1717 ($\nu_{as}$ C=O), 1686 ($\nu$ NHC=O), 1590 ($\nu$ $C{=}C_{arom}$).

*1-(3-{4-[(Butoxycarbonyl)amino]benzoyloxy}-2-hydroxypropyl)-4-(2-methoxyphenyl)piperazin-1-ium chloride (**12**).* $^1$H NMR (200 MHz, DMSO-$d_6$), $\delta_H$ (ppm): 10.65 (s, 1H, $N\underline{H}^+$), 10.12 (s, 1H, $N\underline{H}COO$), 7.98 (d, $J$ = 8.7 Hz, 2H, Ar–$\underline{H}$), 7.63 (d, $J$ = 8.8 Hz, 2H, Ar–$\underline{H}$), 7.07–6.85 (m, 4H, Ar–$OCH_3$), 4.53–4.40 (m, 1H, $C\underline{H}OH$), 4.22 (d, $J$ = 4.3 Hz, 2H, $COOC\underline{H}_2$), 4.10 (t, $J$ = 6.5 Hz, 2H, $C\underline{H}_2CH_2CH_2CH_3$), 3.79 (s, 3H, $OC\underline{H}_3$), 3.73–3.03 (m, 10H, $\underline{H}_{pip}$ + $C\underline{H}_2N_{pip}$), 1.68–1.54 (m, 2H, $CH_2C\underline{H}_2CH_2CH_3$), 1.47–1.28 (m, 2H, $CH_2CH_2C\underline{H}_2CH_3$), 0.91 (t, $J$ = 7.2 Hz, 3H, $CH_2CH_2CH_2C\underline{H}_3$); $^{13}$C NMR (50 MHz, DMSO-$d_6$), $\delta_C$ (ppm): 165.1, 153.4, 151.8, 144.0, 139.4, 130.6, 123.3, 122.8, 120.8, 118.2, 117.3, 112.0, 66.0, 64.2, 63.3, 58.4, 55.3, 52.6, 51.3, 46.7, 46.6, 30.4, 18.5, 13.5; FT-IR (ZnSe ATR, $cm^{-1}$): 3314 ($\nu$ N–H), 3274 ($\nu$ O–H), 2971 ($\nu$ C–H), 2562 ($\nu$ $NH^+$), 1720 ($\nu_{as}$ C=O), 1688 ($\nu$ NHC=O), 1594 ($\nu$ $C{=}C_{arom}$).

*1-(2-Hydroxypropyl-3-{4-[(methoxycarbonyl)amino]benzoyloxy})-4-phenylpiperazin-1-ium chloride (**I**).* $^1$H NMR (200 MHz, DMSO-$d_6$), $\delta_H$ (ppm): 10.62 (br s, 1H, $N\underline{H}^+$), 10.16 (s, 1H, $N\underline{H}COO$), 7.98 (d, $J$ = 8.8 Hz, 2H, Ar–$\underline{H}^{2,6}$), 7.62 (d, $J$ = 8.8 Hz, 2H, Ar–$\underline{H}^{3,5}$), 7.30–7.22 (m, 2H, Ar–$N_{pip}$), 7.02–6.98 (m, 2H, Ar–$N_{pip}$), 6.89–6.82 (m, 1H, Ar–$N_{pip}$), 6.07 (br s, 1H, $O\underline{H}$), 4.46 (br s, 1H, $C\underline{H}(OH)$), 4.23–4.21 (m, 2H, $COOC\underline{H}_2$), 3.83–3.60 (m, 4H, $\underline{H}_{pip}$), 3.69 (s, 3H, $C\underline{H}_3$), 3.29–3.09 (m, 6H, $\underline{H}_{pip}$ + $C\underline{H}_2N_{pip}$); $^{13}$C NMR (50 MHz, DMSO-$d_6$), $\delta_C$ (ppm): 165.1, 153.7, 149.5, 143.9, 130.6, 129.0, 122.9, 119.8, 117.3, 115.8, 66.0, 63.3, 58.2, 52.1, 51.8, 50.7, 45.2; FT-IR (ZnSe ATR, $cm^{-1}$): 3381 ($\nu$ N–H), 3272 ($\nu$ O–H), 2970 ($\nu_{al}$ C–H), 2567 ($\nu$ $NH^+$), 1740 ($\nu_{as}$ C=O), 1701 ($\nu$ NHC=O), 1601 ($\nu$ $C{=}C_{arom}$).

*1-(3-{4-[(Ethoxycarbonyl)amino]benzoyloxy}-2-hydroxypropyl)-4-phenylpiperazin-1-ium chloride (**II**).* $^1$H NMR (200 MHz, DMSO-$d_6$), $\delta_H$ (ppm): 10.75 (s, 1H, $N\underline{H}^+$), 10.12 (s, 1H, $N\underline{H}COO$), 7.98 (d, $J$ = 8.6 Hz, 2H, Ar–$\underline{H}^{2,6}$), 7.63 (d, $J$ = 8.6 Hz, 2H, Ar–$\underline{H}^{3,5}$), 7.30–7.22 (m, 2H, Ar–$N_{pip}$), 7.02–6.98 (m, 2H, Ar–$N_{pip}$); 6.89–6.82 (m, 1H, Ar–$N_{pip}$), 6.07 (br s, 1H, $O\underline{H}$), 4.47 (br s, 1H, $C\underline{H}(OH)$), 4.23–4.10 (m, 4H, $COOC\underline{H}_2$ + $C\underline{H}_2CH_3$), 3.83–3.61 (m, 4H, $\underline{H}_{pip}$), 3.39–3.10 (m, 6H, $\underline{H}_{pip}$ + $C\underline{H}_2N_{pip}$), 1.25 (t, $J$ = 7.1 Hz, 3H, $C\underline{H}_3$); $^{13}$C NMR (50 MHz, DMSO-$d_6$), $\delta_C$ (ppm): 165.1, 153.3, 149.5, 144.0, 130.6, 129.0, 122.8, 119.8, 117.3, 115.8, 66.0, 63.3, 60.5, 58.2, 52.1, 50.8, 45.2, 14.4; FT-IR (ZnSe ATR, $cm^{-1}$): 3375 ($\nu$ N–H), 3260 ($\nu$ O–H), 2978 ($\nu$ C–H), 2567 ($\nu$ $NH^+$), 1736 ($\nu_{as}$ C=O), 1702 ($\nu$ NHC=O), 1597 ($\nu$ $C{=}C_{arom}$).

*1-(2-Hydroxypropyl-3-{4-[(propoxycarbonyl)amino]benzoyloxy})-4-phenylpiperazin-1-ium chloride (**III**).* $^1$H NMR (200 MHz, DMSO-$d_6$), $\delta_H$ (ppm): 10.68 (s, 1H, $N\underline{H}^+$), 10.13 (s, 1H, $N\underline{H}COO$), 7.98 (d, $J$ = 8.8 Hz, 2H, Ar–$\underline{H}^{2,6}$), 7.63 (d, $J$ = 8.8 Hz, 2H, Ar–$\underline{H}^{3,5}$), 7.30–7.22 (m, 2H, Ar–$N_{pip}$), 7.02–6.98 (m, 2H, Ar–$N_{pip}$), 6.89–6.82 (m, 1H, Ar–$N_{pip}$), 6.07 (br s, 1H, $O\underline{H}$), 4.46 (br s, 1H, $C\underline{H}(OH)$), 4.23–4.21 (m, 2H, $COOC\underline{H}_2$), 4.06 (t, $J$ = 6.7 Hz, 2H, $C\underline{H}_2CH_2CH_3$), 3.83–3.61 (m, 4H, $\underline{H}_{pip}$), 3.29–3.10 (m, 6H, $\underline{H}_{pip}$ + $C\underline{H}_2N_{pip}$), 1.73–1.56 (m, 2H, $CH_2C\underline{H}_2CH_3$), 0.93 (t, $J$ = 7.4 Hz, 3H, $CH_2CH_2C\underline{H}_3$); $^{13}$C NMR (50 MHz, DMSO-$d_6$), $\delta_C$ (ppm): 165.1, 153.4, 149.5, 143.9, 130.5, 129.0, 122.8, 119.8, 117.3, 115.8, 66.0, 65.9, 63.3, 58.2, 52.1, 50.7, 45.1, 21.7, 10.1; FT-IR (ZnSe ATR, $cm^{-1}$): 3375 ($\nu$ N–H), 3256 ($\nu$ O–H), 2969 ($\nu$ C–H), 2565 ($\nu$ $NH^+$), 1734 ($\nu_{as}$ C=O), 1702 ($\nu$ NHC=O), 1598 ($\nu$ $C{=}C_{arom}$).

*1-(3-{4-[(Butoxycarbonyl)amino]benzoyloxy}-2-hydroxypropyl)-4-phenylpiperazin-1-ium chloride (**IV**).* $^1$H NMR (200 MHz, DMSO-$d_6$), $\delta_H$ (ppm): 10.71 (s, 1H, $N\underline{H}^+$), 10.11 (s, 1H, $N\underline{H}COO$), 7.98 (d, $J$ = 8.8 Hz, 2H, Ar–$\underline{H}^{2,6}$), 7.63 (d, $J$ = 8.8 Hz, 2H, Ar–$\underline{H}^{3,5}$), 7.30–7.22 (m, 2H, Ar–$N_{pip}$), 7.02–6.98 (m, 2H, Ar–$N_{pip}$), 6.89–6.82 (m, 1H, Ar–$N_{pip}$), 6.07 (br s, 1H, $O\underline{H}$), 4.47 (br s, 1H, $C\underline{H}(OH)$), 4.23–4.21 (m, 2H, $COOC\underline{H}_2$), 4.11 (t, $J$ = 6.5, 2H, $C\underline{H}_2CH_2CH_2CH_3$), 3.83–3.61 (m, 4H, $\underline{H}_{pip}$), 3.39–3.21 (m, 6H, $\underline{H}_{pip}$ + $C\underline{H}_2N_{pip}$), 1.68–1.54

(m, 2H, $CH_2C\underline{H}_2CH_2CH_3$), 1.47–1.29 (m, 2H, $CH_2CH_2C\underline{H}_2CH_3$), 0.91 (t, $J$ = 7.3 Hz, 3H, $CH_2CH_2CH_2C\underline{H}_3$); $^{13}$C NMR (50 MHz, DMSO-$d_6$), $\delta_C$ (ppm): 165.1, 153.4, 149.5, 144.0, 130.6, 129.0, 122.8, 119.8, 117.3, 115.8, 66.0, 64.2, 63.3, 58.2, 52.1, 50.8, 45.2, 30.4, 18.5, 13.5; FT-IR (ZnSe ATR, cm$^{-1}$): 3375 ($\nu$ N–H), 3264 ($\nu$ O–H), 2956 ($\nu$ C–H), 2567 ($\nu$ $NH^+$), 1735 ($\nu_{as}$ C=O), 1701 ($\nu$ NHC=O), 1598 ($\nu$ C=$C_{arom}$).

The $^1$H NMR, $^{13}$C NMR and IR spectral values were consistent with the proposed structure of the compounds (**1**)–(**12**) and (**I**)–(**IV**).

*Notes to Labeling of the Compounds (**1**)–(**12**)*

Initial research focusing on several sets of variously substituted salicylanilides as well as corresponding bioisosteric compounds, and their in vitro antimycobacterial activity showed that an increase in anti- -*Mtb* $H_{37}R_v$ efficacy of these derivatives was related to the presence of a moiety attached to an aromatic ring (within their amide portion). This group was characterized by a relatively higher value of a Hammett constant ($\sigma$), that is, electron-withdrawing properties, and hydrophobic constant ($\pi$). In addition, linearity of these lipophilic molecules was considered a favorable structural feature [95,96,97]. This was a reason why the 4′-*F*-substituted derivatives (**1**)–(**4**) were prioritized in antimycobacterial screening [6] and formal labelling (Figure S1) over the compounds (**5**)–(**12**).

*Notes to Labeling of the Compounds (**I**)–(**IV**)*

The purpose to use different numeral types for indication of particular arylcarbonyloxypiperazinopropanol-based compounds, that is, the series (**1**)–(**12**) versus (**I**)–(**IV**), was to distinguish the derivatives already antimycobacterially in vitro screened (Arabic numerals were used for that indication) and the molecules that were antimycobacterially in vitro evaluated currently (Latin / Roman numerals were employed for that indication). Thus, the evaluations of particular structural, physicochemical, pharmacokinetic and pharmacodynamic properties of non-protonated compounds (**Ib**)–(**IVb**) and their salts (**I**)–(**IV**) were listed in the current paper before those of (**1b**)–(**12b**) and (**1**)–(**12**).

***Relationships Between Structural, Physicochemical and Pharmacokinetic Properties of Non--Protonated Compounds (IVb), (1b)–(4b), (7b), (8b) and (10b)–(12b) Which Salts Showed MICs ≤ 62.5 μM – More Detailed Information***

*Principal Component Analysis*

In regard to such a selection of the derivatives, that is, (**IVb**), (**1b**)–(**4b**), (**7b**), (**8b**) and (**10b**)–(**12b**), suitable PC 1, PC 2 and PC 3 accounted for 91.16% of the total variance in a respective PCA and their contributions were the following: PC 1 (60.51%, $\lambda_e$ = 7.26), PC 2 (19.79%, $\lambda_e$ = 2.37) and PC 3 (10.86%, $\lambda_e$ = 1.30). The relationship between PC 1 and PC 2 (accounted for total variance of 80.30%) allowed for the distribution of these non-protonated compounds into several subcategories.

All involved active variables (parameters) were projected onto a selected 2D coordinate system of PC 1 × PC 2. The compounds (**1b**)–(**3b**), (**7b**) and (**10b**), that is, active observations, could be found on a left side within Figure S9(**a**) (Supplementary Materials) and were defined by PC 1 < 1.000, *TPSA* ≤ 356.77 Å$^2$, *MR* ≤ 131.36 m$^3$/mol, log $P_{av}$ ≤ 3.40 and *MolVol* ≤ 454.73 Å$^3$, as well as by relatively higher *QED* ≥ 0.55. Slightly higher values of particular structural and physicochemical parameters were related to another series of active observations, that is, the derivatives (**IVb**), (**4b**), (**8b**), (**11b**) and (**12b**). These molecules showed lower *QED* data compared to those being included in a previous set (Tables 4 and S1).

The cos$^2$ descriptors for the most optimally presented active variables within a circle of correlation (Figure S9(**b**); Supplementary Materials) were as follows: 0.983 (*TPSA*; this value was related to PC 1), 0.975 (F*sp*$^3$; PC 1), 0.962 (*MR*; PC 1), 0.955 (*MolVol*; PC 1) and 0.941 (*MW*; PC 1). The individual pharmacokinetic parameters provided lower cos$^2$ values of 0.660 (IA; PC 2), 0.395 (log $P_{app}$; PC 1), 0.221 (log PS; PC 2) and 0.129 (log BB; PC 1). The *QED* variable was defined by cos$^2$ = 0.815 (PC 1).

A relatively close positive correlation was observed between particular pairs of descriptors from a set of active variables consisting of *MW*, F*sp*$^3$, *TPSA*, *MR* and *MolVol*, as visualized in Figure S9(**b**). These relationships were characterized by the *r* data of ≥ 0.935. The log $P_{av}$ parameter relatively most positively correlated with F*sp*$^3$ (*r* = 0.730) and *MW* (*r* = 0.700). The strongest negative correlation was calculated between a given lipophilicity descriptor and log $P_{app}$ (*r* = –0.818), as well as between log $P_{av}$ and *QED* (*r* = –0.798) (Table S13; Supplementary Materials).

In addition, the increase in *MW*, F*sp*$^3$, *TPSA*, *MR* and *MolVol* strongly negatively affected the *QED* index generated for these compounds. This observation was supported by the *r* values of –0.841, –0.888, –0.884, –0.884 and –0.857 (Table S13).

## C. FIGURES

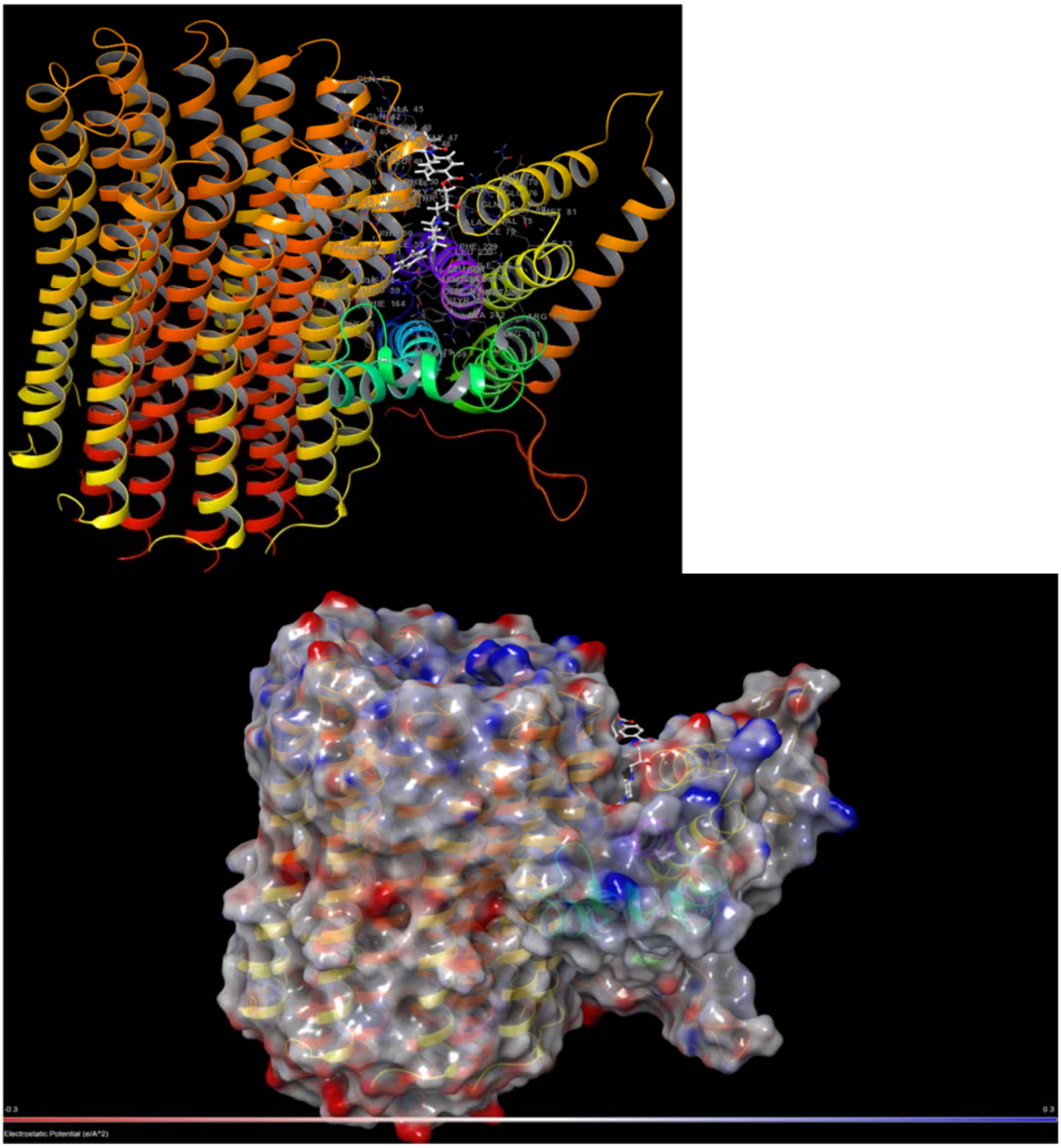


**Figure S1.** The binding mode of the compound (***R***)-(**IV**) within an active site of ATP synthase present in *Mtb* $H_{37}R_v$.

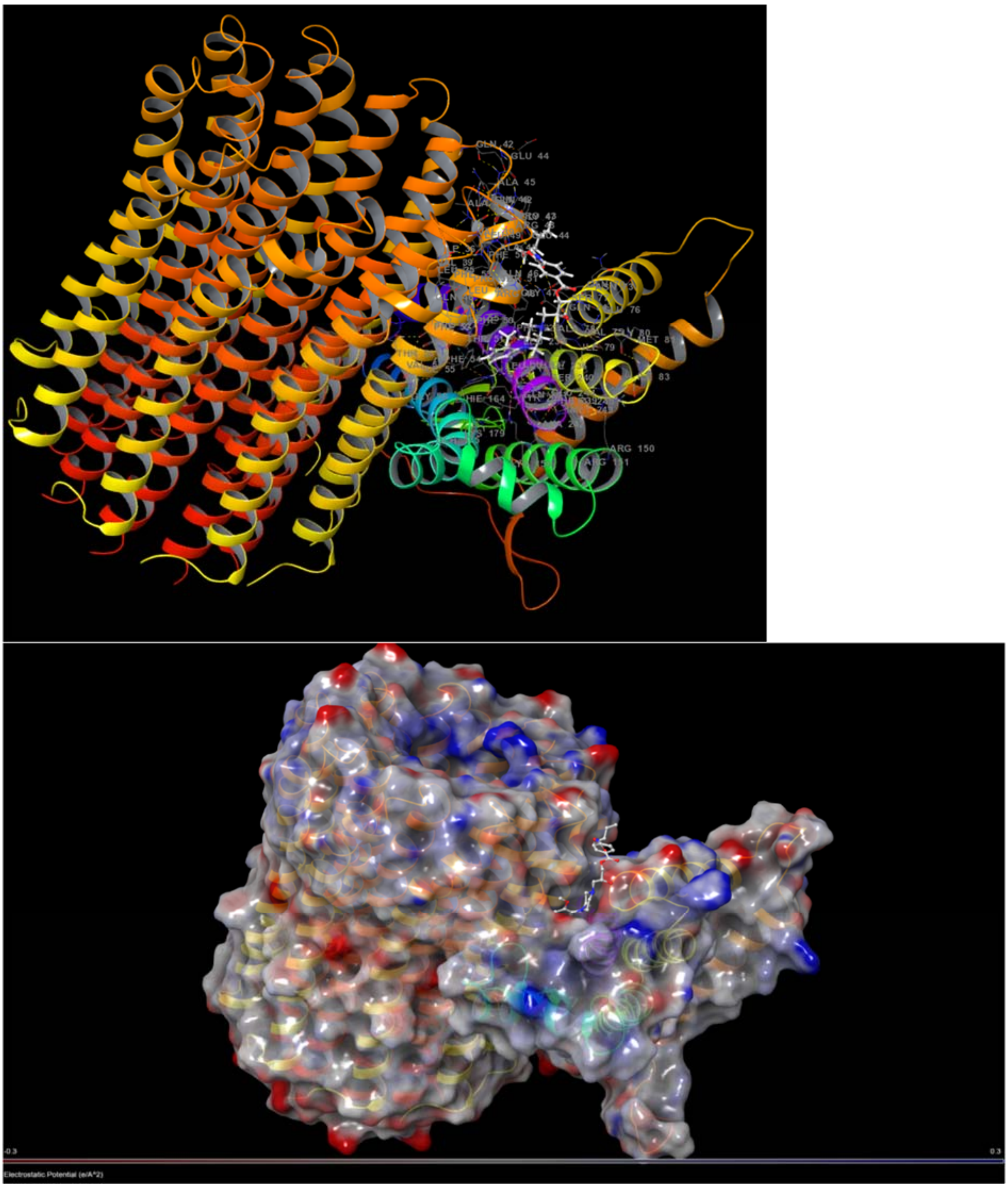


**Figure S2.** The binding mode of the compound (***R***)-(**12**) within an active site of ATP synthase present in *Mtb* $H_{37}R_v$.

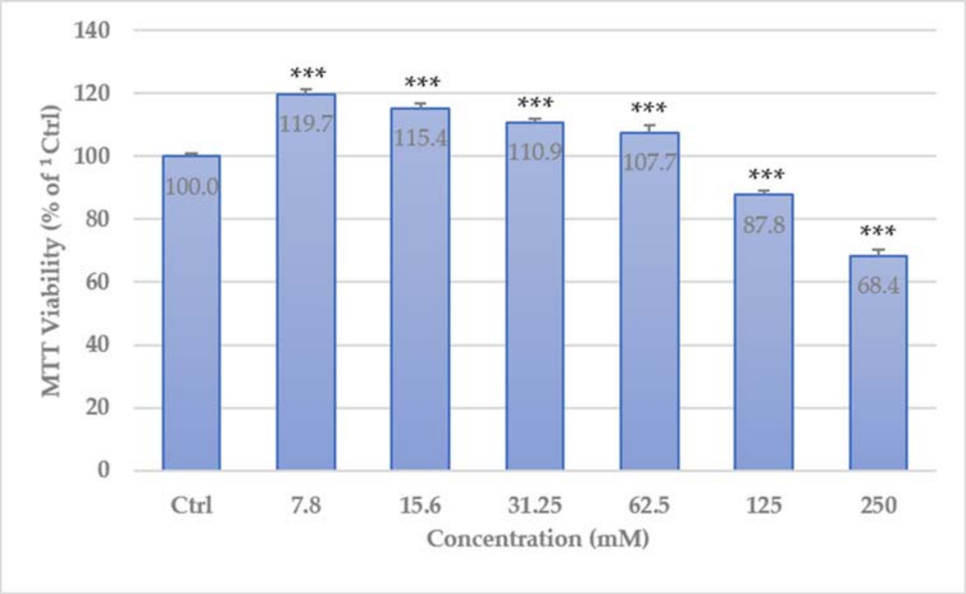


**Figure S3.** The effect of different concentrations of the compound (**IV**) on normal human dermal fibroblast viability (in percentages).

***Notes to Figure S3***

[1] Ctrl = control. The indication of a significance level ($p$ value) was as follows: *** (three stars) = extremely significant.

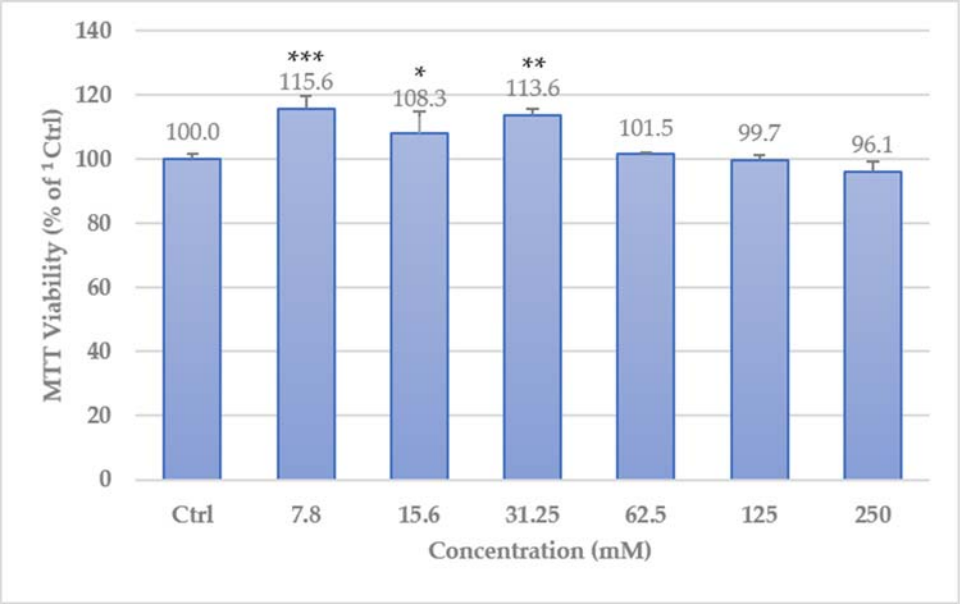


**Figure S4.** The effect of different concentrations of the compound (**12**) on normal human dermal fibroblast viability (in percentages).

***Notes to Figure S4***

[1] Ctrl = control. The indication of a significance level (*p* value) was as follows: * (one star) = significant, ** (two stars) = very significant, *** (three stars) = extremely significant.

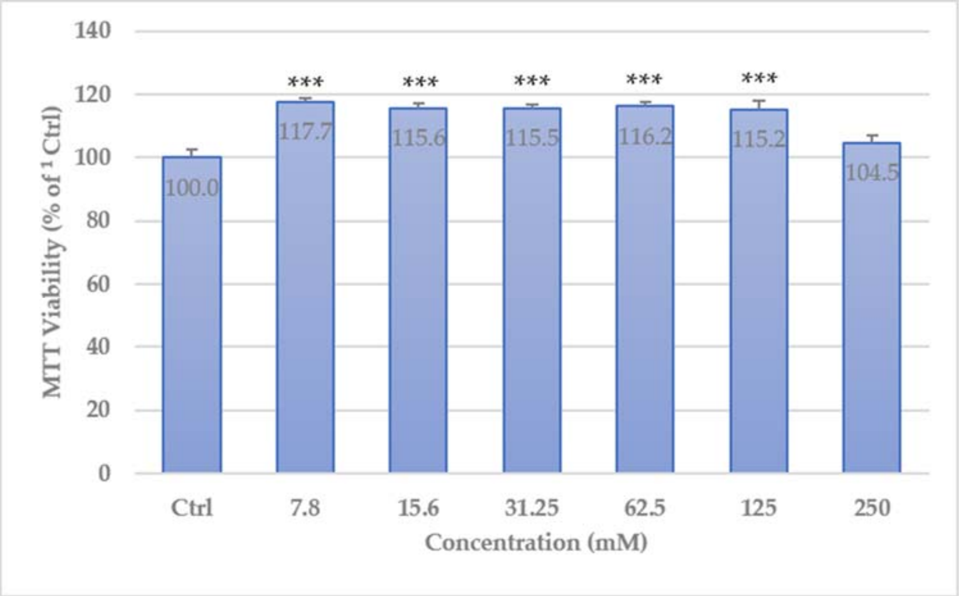


**Figure S5.** The effect of different concentrations of isoniazid (INH) on normal human dermal fibroblast viability (in percentages).

***Notes to Figure S5***

[1] Ctrl = control. The indication of a significance level (*p* value) was as follows: *** (three stars) = extremely significant.

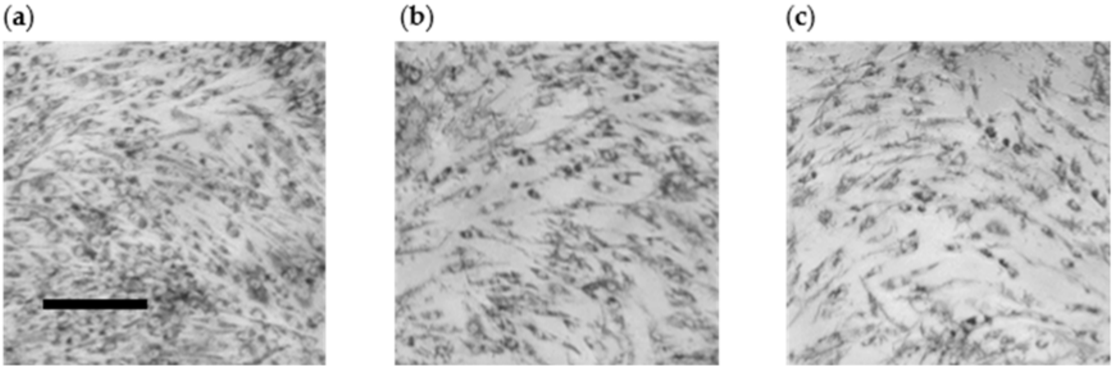

**Figure S6.** The (**a**) non-treated normal human dermal fibroblast cells (control) and normal human dermal fibroblasts treated with the compound (**IV**) at (**b**) $c$ = 125 μM and (**c**) $c$ = 250 μM and incubated with a 3-(4,5-dimethylthiazol-2-yl)-2,5-diphenyl-2*H*-tetrazolium bromide (MTT) reagent.

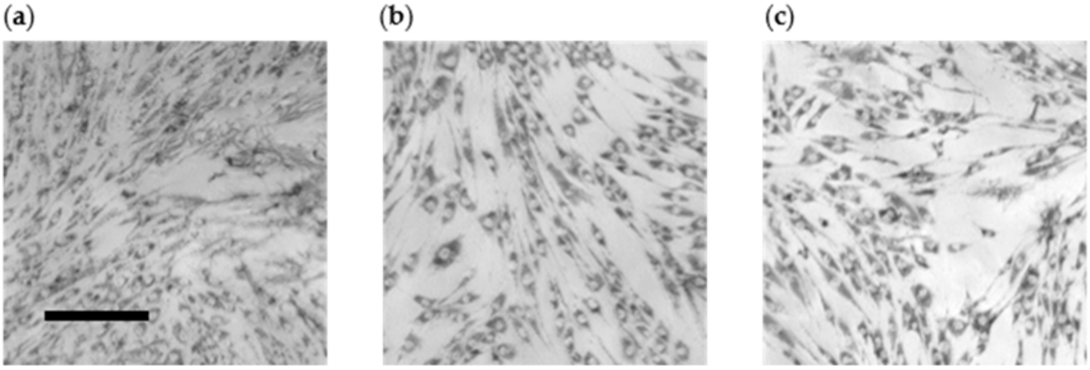

**Figure S7.** The (**a**) non-treated normal human dermal fibroblast cells (control) and normal human dermal fibroblasts treated with the compound (**12**) at (**b**) $c$ = 125 μM and (**c**) $c$ = 250 μM and incubated with a 3-(4,5-dimethylthiazol-2-yl)-2,5-diphenyl-2*H*-tetrazolium bromide (MTT) reagent.

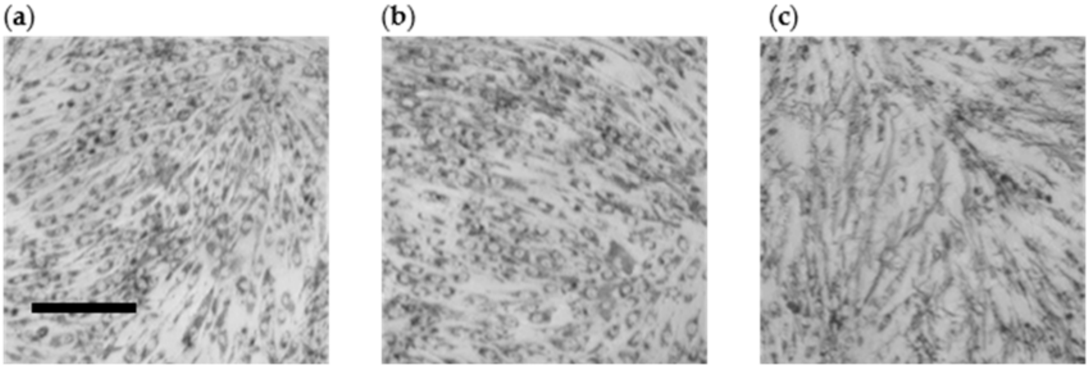

**Figure S8.** The (**a**) non-treated normal human dermal fibroblast cells (control) and normal human dermal fibroblasts treated with isoniazid (INH) at (**b**) $c$ = 125 μM and (**c**) $c$ = 250 μM and incubated with a 3-(4,5-dimethylthiazol-2-yl)-2,5-diphenyl-2*H*-tetrazolium bromide (MTT) reagent.

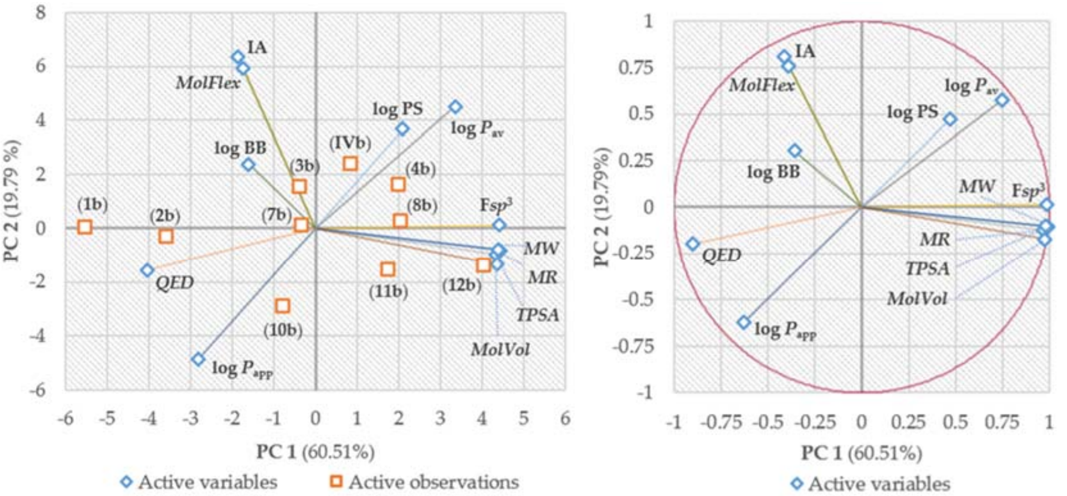


(**a**) (**b**)

**Figure S9.** Graphical presentation of the relationships between structural, physicochemical and pharmacokinetic parameters of selected non-protonated compounds which salts relatively effectively inhibited growth of *Mtb* $H_{37}R_v$: (**a**) 2D biplot mapping PC 1 and PC 2 scores of these compounds and loadings of particular variables, that is, *MW*, F*sp*$^3$, *TPSA*, *MolFlex*, *MR*, *MolVol*, log $P_{av}$, IA, log $P_{app}$, log BB, log PS and *QED*; (**b**) 2D mapping of loadings of variables indicating their (i) positions within a circle of correlation; (ii) relationships with both PC 1 and PC 2.

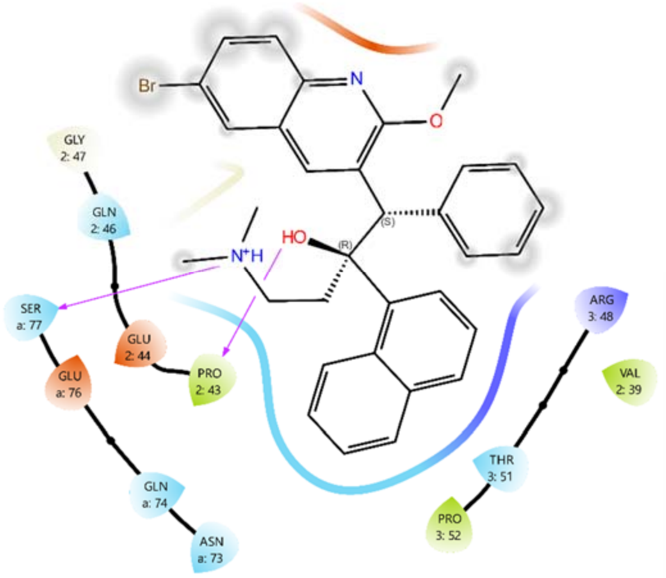


**Figure S10.** The 2D diagram showing particular interactions between the (**1***S*,**2***R*)-stereoisomer of bedaquiline (BDQ; **17**) and individual amino acid (AA) residues present within an active site (binding pocket) of ATP synthase in *Mtb* $H_{37}R_v$. Hydrophobic interactions are indicated in grey, purple arrows indicate hydrogen bonds.

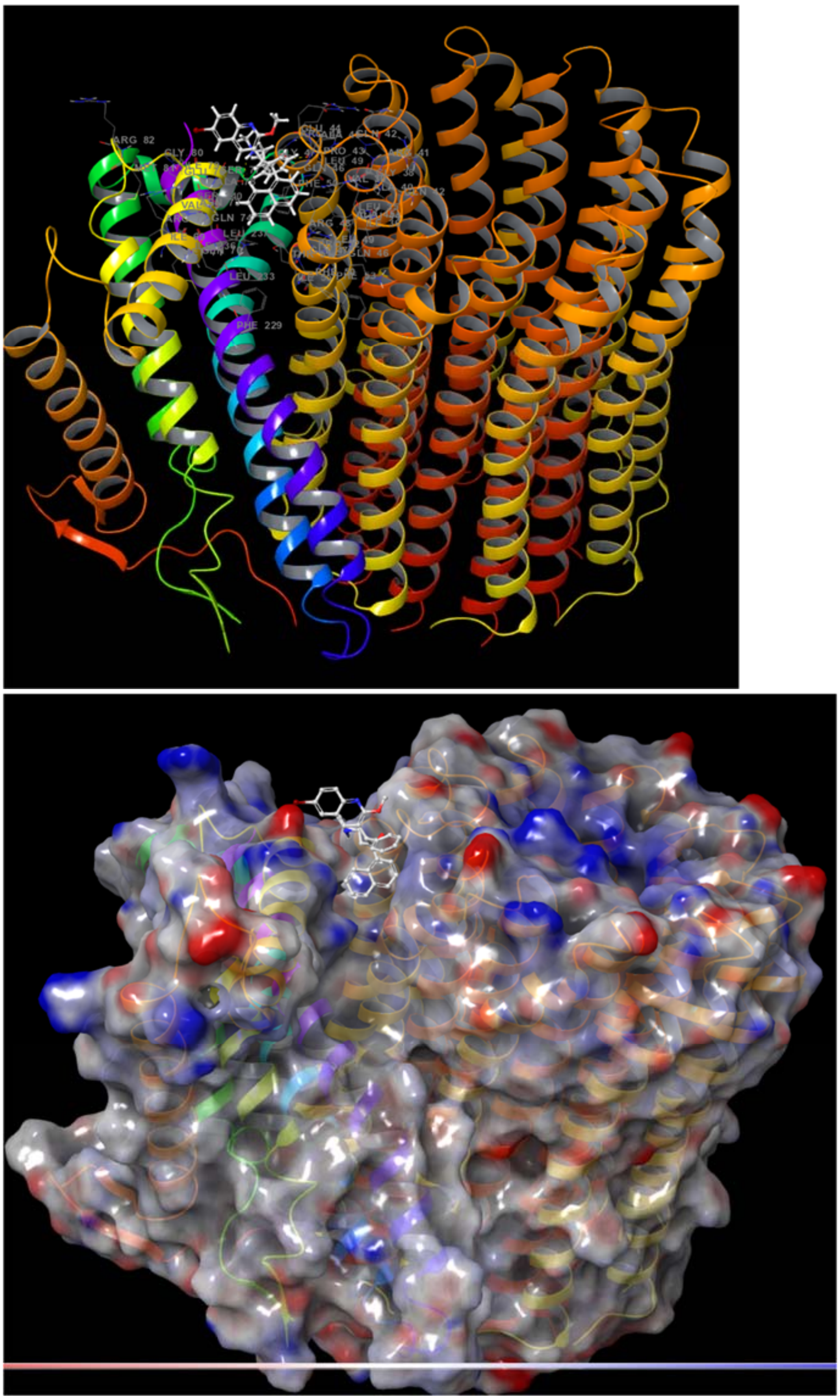


**Figure S11.** The mode in which the (1*S*,2*R*)-stereoisomer of bedaquiline (BDQ; **17**) was docked into an active site of ATP synthase present in *Mtb* $H_{37}R_v$.
